# Equine trypanosomiasis, a systematic review and meta‐analyses: Prevalence, morbidity and mortality

**DOI:** 10.1111/evj.70101

**Published:** 2025-10-23

**Authors:** Alexandra G. Raftery, Lauren Gummery, Karelhia Garcia, Dinesh Mohite, Paul Capewell, David G. M. Sutton

**Affiliations:** ^1^ School of Biodiversity, One Health and Veterinary Medicine, College of Medical, Veterinary and Life Sciences, University of Glasgow Glasgow UK; ^2^ Three Counties Equine Hospital, Stratford Bridge Gloucestershire UK; ^3^ Faculty of Veterinary Medicine, Veterinary Medical Sciences University of Calgary Calgary Canada; ^4^ Federation of Indian Animal Protection Organisations New Dehli India; ^5^ School of Molecular Biosciences, College of Medical, Veterinary and Life Sciences University of Glasgow Glasgow UK

**Keywords:** donkey, dourine, horse, Nagana, Surra, trypanosomiasis

## Abstract

**Background:**

Equine trypanosomiasis is a neglected protozoal disease.

**Objectives:**

To perform a systematic search of literature to explore: (1) In equines what is the global geographical distribution and prevalence of trypanosomiasis? In low and middle‐income countries (LMICs) is trypanosomiasis more prevalent than in higher‐income countries (HICs)? (2) Is trypanosomiasis infection a significant contributor to global morbidity and mortality?

**Study Design:**

Systematic review and meta‐analyses.

**Methods:**

Studies were identified that described naturally occurring equine trypanosomiasis worldwide following ‘Preferred Reporting Items for Systematic Reviews and Meta‐Analyses’ using eight international databases (1980–2022). Equine population data for each country were extracted. Meta‐analyses were used to estimate point prevalence and disease characteristics. Country exposure risk to equines (negligible/low/medium/high) and clinical data (*Trypanosoma* sp.; outbreak (O) vs. endemic (E) disease) were categorised.

**Results:**

Study quality was assessed (Question 1 prevalence: *n* = 147 manuscripts, median grade ‘medium’ (4/8 (range 2–6)); Question 2 morbidity and mortality: *n* = 46 ‘moderate’ (*n* = 1), ‘low’ (*n* = 20) or ‘very low’ (*n* = 25)). Heterogeneity was high. LMICs were more likely to report disease (41/125; 33% vs. 7/80, 9%; (*p* < 0.001; OR 5.1 (2.1–14.2))). Fifty‐six percent of the world's equines reside in a ‘medium’/‘high’ risk country (61,507,601). Disease characteristics were summated. For *Trypanosoma evansi*: (O) Infection rate (IR) (42%; 95% CI 14–76), morbidity (47%; (13–85)), mortality (23%; 7–54) and death to case ratio (DCR) (45%; 20–73). *Trypanosoma equiperdum*: (O) IR 12% (7–18), morbidity 25% (9–49). *Tsetse transmitted trypanosomiasis* (O): IR 46% (29–63), morbidity 46% (29%–63%), mortality 6% (1–19), DCR 12% (2–38). (E) IR 50% (20–60), morbidity (no data), mortality 11% (7–14), DCR 9% (5–16). *Trypanosoma vivax* (O) IR 43% (10–83), morbidity 43% (10–83), mortality 15% (0–100), DCR 32% (0–100).

**Main Limitations:**

Publication bias, heterogeneity, descriptive data, missing data.

**Conclusions:**

Equine trypanosomiasis predominates in LMICs. Conservatively, globally more than eight million equines are estimated to be affected, with substantial morbidity and mortality.

## 1 INTRODUCTION

For thousands of years, the health and productivity of horses, donkeys, and mules on the African continent has been opposed by an invisible barrier, the tsetse belt. Harbouring the tsetse fly, this 10 million km^2^ ^1^ expanse of land, covering 37 countries,^2^ runs from West to East Africa. Within this region, the blood‐sucking tsetse fly (*Glossina* sp.) is the predominant vector for trypanosomes. Trypanosomes are capable of causing severe disease in many mammals, including humans, cattle, and equines. Subsequent adaptations (dys‐ or akinetoplasty) of certain *Trypanosoma* species to alternative transmission routes have considerably increased the geographically at‐risk equine population, particularly in Asia and Central and South America.

Equine trypanosomiasis (affecting horses, donkeys and mules) results in three disease syndromes with overlapping clinical signs. Colloquially, the syndromes are known as Nagana, Surra, and Dourine, and are clinically defined by the mode of transmission that also determines their geographical distribution. Disease results from infection with one or multiple of three species of trypanosome (*Trypanosoma vivax*, *Trypanosoma congolense* and *Trypanosoma brucei* derived species).^3^, ^4^ Clinical signs reflect haemolymphatic pathology (*T. congolense*, *T. vivax*, *T. brucei* species) with presenting anaemia, pyrexia, tachycardia, lethargy, and weight loss and neuropathology (*T. brucei* derived species) resulting in ataxia, weakness, recumbency, and frequent progression to death.^3^, ^4^, ^5^, ^6^ The impact on welfare and productivity for the infected equine is therefore significant.

The tsetse fly is an extremely effective vector of trypanosomiasis. This is exemplified by the high burden of livestock disease encountered within the tsetse belt (Nagana).^3^, ^4^, ^7^, ^8^, ^9^, ^10^ The wide distribution of disease beyond these geographical boundaries is attributed to adaptations of three *Trypanosoma* sp. to no longer rely upon the tsetse fly as a vector. These trypanosomes are responsible for Dourine (*Trypanosoma equiperdum*) which is venereally transmitted^11^ and Surra (*Trypanosoma evansi*) which is mechanically transmitted by biting flies^12^ (and vampire bats in South America). A distinct strain of *T. vivax*, also mechanically transmitted, is prevalent in South America and is thought to originate from the import of West African Zebu cattle that followed the routes of the African slave trade.^13^, ^14^ The negative contribution of livestock trypanosomiasis on welfare and productivity of cattle and small ruminants has been increasingly recognised.^9^, ^15^, ^16^

Globally, working equines have a continued and growing socioeconomic role in supporting the livelihoods of between 300 and 600 million people, often within the most vulnerable communities.^17^ Working equines contribute significantly to household income,^18^, ^19^, ^20^ are used for transport and traction,^21^ and create opportunities for women and children^19^, ^22^ (Sustainable Development Goals 1, 2, 5^23^). The world equine population (mules, donkeys and horses) is estimated to be just over 127 million (FAO, 2014^24^) and approximately 85% are working equines in low income countries.^25^ The positive impact of working equines upon poverty reduction, gender equality and environmental stability^26^, ^27^ provides impetus to tackle the myriad of issues hindering their welfare and productivity.

Trypanosomiasis has been suggested to be a significant contributor to global equine morbidity and mortality^27^ but the data to confirm this hypothesis have not been objectively assessed. We hypothesised that objective analysis would broadly support this statement but that factors relating to study quality and gaps in available knowledge would lead to problems in having high confidence in conclusions.

The aim of the study was therefore to consolidate existing knowledge and identify areas where future challenge led research is required. To assimilate the current knowledge and understanding of equine trypanosomiasis, a systematic review of peer‐reviewed and grey literature (scientific data not published through academic channels but presented within documents such as policy documents, theses and dissertations; included to reduce publication bias) was performed following the Preferred Reporting Items for Systematic Reviews and Meta‐analyses (PRISMA) guidelines^28^ and Synthesis without meta‐analysis (SWiM) in systematic reviews reporting guidelines.^29^

The objectives of this study were to answer the following two questions:

Study question 1: What is the global geographical distribution and prevalence of equine trypanosomiasis? In equines residing in low‐ and middle‐income countries (*Population*) is trypanosomiasis (*Intervention*) more prevalent (*Outcome*) than in those in higher‐income countries (*Comparison*)?

Study question 2: In equines (*Population*) is trypanosomiasis infection (*Intervention*) a significant contributor to global morbidity and mortality (*Outcome*)?

## 2 METHODS

### 2.1 General methods

**FIGURE 1:**
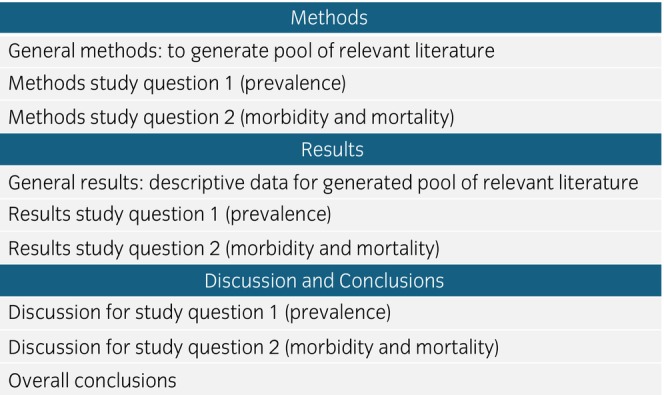
Diagram illustrating the methodological approach and structure of this systematic review of the prevalence, morbidity and mortality of equine trypanosomiasis. Each study question uses specific methodology that is applied to the pool of selected relevant literature generated from the ‘General methods’.

The detailed study protocol is available (Data S1) following PRISMA^28^ and SWiM guidelines.^29^ The structure of this systematic review is outlined in Figure 1.

#### 2.1.1 Amendments

Post‐hoc amendments to the study protocol (Data S1) are evident by comparison with the original pre‐registered protocol (available at osf.io/r6fsw).

#### 2.1.2 Search strategy

References for this review were identified through searches of eight international databases and grey literature (search strategies summarised in Table 1, detailed in study protocol (Data S1)). Broad search terms were chosen to capture studies on any aspect of equine trypanosomiasis. Publication year was restricted to between January 1980 and July 2022 to improve the relevance of the data to current times whilst aiming to represent data from the maximum number of countries, as published surveillance was anticipated to be sporadic.

**TABLE 1:** Search strategy for systematic review of equine trypanosomiasis publications: Full search criteria for database searches.

Search engine	Terms
Africa‐index Medicus (WHO Global Health Library)	equine trypanosomiasis
CAB International: CAB Abstracts and Global Health	(((“equine”) OR (“equid*”) OR (“horse”) OR (“donkey”) OR (“mule”)) AND ((“trypanosom*”) OR (“surra”) OR (“nagana”) OR (“dourine”) OR (“vivax”) OR (“congolense”) OR (“brucei”) OR (“equiperdum”)) Publication year: 1980‐ present
CABI VetMed Resource	(((“equine”) OR (“equid*”) OR (“horse”) OR (“donkey”) OR (“mule”)) AND ((“trypanosom*”) OR (“surra”) OR (“nagana”) OR (“dourine”) OR (“vivax”) OR (“congolense”) OR (“brucei”) OR (“equiperdum”) OR (“evansi”) OR (“mal de caderas”))) AND yr.: [1980 TO 2020]
Pubmed	(“trypanosomiasis”[MeSH Terms] OR “trypanosomiasis”[All Fields]) OR “trypanosomes”[All Fields] OR “trypanosomoses”[All Fields] OR “Surra”[All Fields] OR “Nagana”[All Fields] OR “brucei”[All fields] OR “congolense”[All fields] OR “vivax”[All fields] AND (“horses”[MeSH Terms] OR “horses”[All Fields] OR “equine”[All Fields] OR “donkey”[All Fields] OR “ass”[All Fields] OR “mule”[All Fields] OR “equidae”[All Fields]) AND (“1980/01/01”[PDAT]: “3000/12/31”[PDAT])
Web of science	TS = (horse OR equine OR donkey OR ass OR equid* OR mule) OR TI = (horse OR equine OR donkey OR ass OR equid* OR mule) Language = Auto Timespan = 1980–2020 Databases = WOS, BCI, BIOSIS, CABI, CCC, DRCI, DIIDW, KJD, MEDLINE, RSCI, SCIELO, ZOOREC Search #2 TS = (trypanosom* OR Surra OR Nagana OR brucei OR congolense OR vivax OR evansi OR equiperdum OR mal de caderas OR dourine) OR TI = (trypanosom* OR Surra OR Nagana OR brucei OR congolense OR vivax OR evansi OR equiperdum OR mal de cadaras OR dourine) Language = Auto Timespan = 1980–2020 Databases = WOS, BCI, BIOSIS, CABI, CCC, DRCI, DIIDW, KJD, MEDLINE, RSCI, SCIELO, ZOOREC Search #1 AND #2
EMBASE (Ovid)	Search 1: (equus or equid or equine or horse or donkey or ass or mule or equidae).af. Timespan = 1980–2020 Search 2: (trypanosom* or brucei or congolense or vivax or nagana or surra or dourine or evansi or equiperdum or mal de caderas or african animal trypanosomiasis).af. Timespan = 1980–2020 Search #1 and #2
Zetoc	equine trypanosomiasis
Scopus	TITLE‐ABS‐KEY ((horse OR donkey OR mule OR equine OR equus OR equidae OR equid OR ass) AND (trypanosomiasis OR trypanosoma OR trypanosomoses OR brucei OR congolense OR vivax OR surra OR nagana OR dourine OR equiperdum OR evansi OR “mal de caderas”)) Timespan = 1980–2020

##### Study selection and criteria

The generated pool of relevant literature was then used to apply more specific inclusion and exclusion criteria for this systematic review which contains ‘Study question 1: prevalence’ and ‘Study question 2: Morbidity and mortality’. The abstracts and titles were downloaded and collated (.csv or .xls). The records were adapted into a standardised format to allow consolidation in Excel. This allowed searching for duplicity (performed manually) to remove items listed more than once.

Each title (step one—initial screening for topic relevance) and then title and abstract (step two—abstract review) were reviewed independently by two reviewers (AGR, LG) who were blinded to the decision of the other reviewer. The articles were selected based upon predetermined, standardised inclusion and exclusion criteria. Where reviewers differed in opinion, a third reviewer (DGMS) had the deciding vote.

**FIGURE 2:**
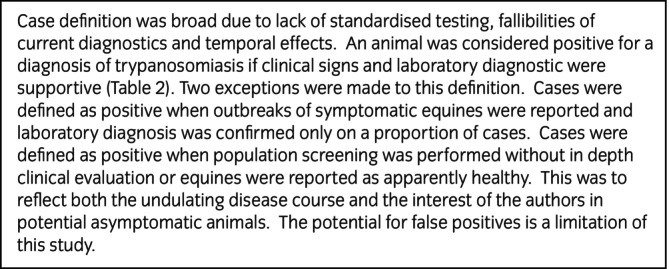
Case definition for diagnosis of equine trypanosomiasis.

*Inclusion criteria for titles and abstracts*:

1. Contained original data on horse, donkey, or mule *Trypanosoma* spp. infection from any country in the world
2. Data complied with case definition (Figure 2; Table 2). Case definition was agreed by AGR, LG and DGMS.
3. All types of study design including case reports were retained to try and obtain the greatest geographical coverage of information since data were anticipated to be sparse/absent in some areas. It was anticipated that the level of evidence would be generally low since randomised controlled trials (RCT) are rare in veterinary medicine and therefore all types of study design were maintained initially to gain the benefits of repeated observations.
4. Abstracts written in English, French, Spanish or Portuguese due to skillset available to the authors.

**TABLE 2:** *Case definition for clinical signs consistent with equine trypanosomiasis and laboratory diagnostics for inclusion of manuscripts*.

	**Clinical signs** consistent with trypanosomiasis:	
		Haemolymphatic
	AND/OR	Neuropathological
AND	**Laboratory diagnostic**	
		Molecular: PCR or LAMP
	OR	Antibody: ELISA, LFA, CATT
	OR	Microscopy: Positive identification of *Trypanosoma* sp. on examination of wet blood smear, buffy coat or Giemsa/Diff quik stained blood smear

*Exclusion criteria for titles and abstracts*:

1. Studies reporting experimental infections.
2. Studies with insufficient information to assess case definition.
3. Studies where full text was not available.
4. Nonsystematic review articles, book chapters, newspaper articles, and other documents that did not contain original data.
5. Grey literature with peer‐reviewed manuscripts containing the same data.

##### Full text review

Articles meeting these criteria then underwent a review of the full text. This included particular attention that the case definition (Figure 2; Table 2) was still fulfilled when the full text had been reviewed. The included articles were collated into Zotero^30^ reference manager to facilitate the review process. Articles requiring full text review in French, Spanish, or Portuguese were translated. Articles were excluded after full text review based on the following exclusion criteria. This review was performed by AR and LG, with DGMS as a deciding vote for differing opinions.

*Exclusion criteria for full text*:

Study did not reach criteria (inclusion/ exclusion) outlined in title and abstract review.

#### 2.1.3 Data extraction and synthesis

A flow chart demonstrating the number of included studies and points of exclusion was made using the PRISMA template^31^ as the study was performed (Figure S1). General information was extracted for each study, including study design and sources of funding (specific details within Study protocol (Data S1), outline database pre‐registration on OSF osf.io/ky8th/ and Table S1, Results). For each study question topic, inclusion and exclusion criteria were generated (study protocol, Data S1; within study question methodology in manuscript) to apply to this pool of relevant collated literature. Data extraction was performed by AGR into a standard form (Table S1, Results); studies included within specific study questions were later re‐reviewed for more detailed descriptive data. Specific details of the data extracted for the general methods and each study question are also provided within the study protocol (Data S1).

##### Extraction of specific data and data analysis

Study questions were devised following discussion with the authors in order to extract information which was most practically useful and could be assimilated and thus achieve the study objectives.

For each of the two questions (Study question 1: Prevalence, Study question 2: Morbidity and mortality), inclusion and exclusion criteria were generated (documented in question specific methods) to select relevant data within the generated dataset. Papers could be included in more than one question and there were some data that were used for multiple questions.

A predominance of exploratory rather than confirmatory studies was expected. Thus, data compilation was planned to be predominantly descriptive through visualisation and consolidation.

*Critical assessment of methodological quality*:

Critical assessment of methodological quality (including risk of bias) was performed at the individual manuscript and the outcome level for each question (details within specific question methods). Heterogeneity of manuscripts for each question was assessed qualitatively and/or quantitatively (where meta‐analyses were used). For each of the two questions, the level of confidence in answering the question was assessed using GRADE^32^ to rate recommendations as strong or weak evidence, in favour of or against. Certainty was rated down for risk of bias, imprecision, inconsistency, indirectness, and publication bias, and certainty was rated up for a large magnitude of effect, dose–response gradient, and residual confounding factors that would increase effect.

*Descriptors of included studies*:

Descriptive statistics of the study designs, publication year and whether the affiliation of the primary author was from the country that the data was collected were presented. For the papers containing a hypothesis, the number of studies reporting positive results was calculated. Sources of funding where reported were recorded for included studies.

### 2.2 Methods for study question 1: Prevalence

This aimed to answer the questions ‘What is the global geographical distribution and prevalence of equine trypanosomiasis?’ and ‘In equines residing in low and middle income countries (LMICs) is trypanosomiasis more prevalent than those in higher income countries?’

Studies were included that reported a population of equines (donkey, horse or mule) screened for *Trypanosoma* spp. using any diagnostic method. Studies were excluded if the number of equines positive or negative by the diagnostic method could not be extracted.

#### 2.2.1 Critical assessment of methodological quality

A quality assessment checklist for prevalence studies was used to assess risk of bias (0–8) (Table S2, adapted^33^). For each country, the quality of evidence was then categorised as low (1 high risk of bias/moderate risk of bias quality source), medium (1 low risk of bias quality source OR 2 or more moderate/high risk of bias quality source) or high (2 or more low risk of bias sources OR 5 or more moderate risk of bias quality sources) based on the number and graded quality of contributing studies. Heterogeneity (clinical, methodological, statistical) was assessed qualitatively for each country and quantitatively for countries where data permitted meta‐analysis.

#### 2.2.2 Assessment of the world equine population at risk from trypanosomiasis infection

Data were extracted pertaining to the tested population and diagnostic methods used and categorised as ‘surveillance’, ‘outbreak’ or ‘clinic’. Sampling methods were recorded. The selected outcome variable was the number of equines positive for 1 or more *Trypanosoma* spp. from the total number of tested equines using any diagnostic method.

The World Organisation of Animal Health, World Animal Health Information System (WAHIS) was used as an additional source of information for outbreak information and disease status.^34^ Country level open source equine disease reporting portals were also screened for evidence of equine trypanosomiasis.^35^, ^36^, ^37^, ^38^

Equine population data for each country (*n* = 206) was extracted from the FAO open source dataset.^39^ Countries were categorised as higher (HIC) or as lower or middle income (LMIC).^40^ Data were used to evaluate five outcomes:

1. Geographical distribution of reported *Trypanosoma* sp. in equines.
2. Comparison of HIC and LMIC risk of exposure: The proportion of LMIC countries reporting equine trypanosomiasis was compared with the proportion in HICs (Chi squared test).
3. Country level prevalence estimation: Country level point prevalence was estimated by meta‐analysis of prevalence of all surveillance and clinic studies (i.e., excluding outbreak reports, case reports and case series) within a country. To combine these data from multiple studies in an individual country two assumptions were made. These were that first, no animal was sampled twice and that second, samples were random. Meta‐analyses using extracted proportion data were performed at the country level using an inverse variance method with a random effects model (with Hartung‐Knapp adjustment) and logit transformation. For individual studies Clopper‐Pearson confidence intervals were calculated. *τ* ^2^ (using a DerSimonian‐Laird estimator) and *I* ^2^ (based on Q) were calculated as quantitative measures of heterogeneity. The results were visualised in forest plots. Analyses were performed using ‘metafor’^41^ and ‘meta’^42^ in R studio.^43^ R script is available in supplementary data (Data S2). The prevalence estimates and 95% confidence intervals were combined with the country equine population data^24^ for an estimate of the number of affected equines within the country. The limitations of using meta‐analysis for country prevalence include the regional variation in prevalence and variation in diagnostic methods. Where more than one diagnostic method was reported the results of serological testing were recorded since the aim was to assess exposure risk (including subclinical infections).
4. Summated prevalence and risk assessment for equines in countries with data available: To estimate the total number of affected equines within the countries with available data, direct standardisation using population size weight^44^ was used. Risk of exposure for equines in countries with data available was estimated using prevalence, outbreak and WOAH, WAHIS disease status. Risk of exposure was categorised as no data, negligible, low, medium and high (Table 3) and the number of equines at each risk level was calculated using available country equine population data.^24^
5. Global risk assessment for equine population: A risk assessment for countries with missing data was performed. For countries for which no data were available, a risk assessment for country‐level risk of exposure for equines was performed using a standardised qualitative process. This consisted of consideration of the following questions:
  - Are equine populations reported in this country? (FAOSTAT^24^).
  - Is the disease present within this continent and at what estimated risk level?
  - Is evidence found of disease in other species?
  - Are disease surveillance systems evident?
  - Is the country resource rich or poor (HIC or LMIC)? (World Bank^45^).
  - Are control and/or surveillance measures for trypanosomiasis reported? (WAHIS, WOAH).
  - Is disease reported reliably in the region?
  - Is there a vector present? (i.e., tsetse fly within the tsetse belt).

**TABLE 3:** Country level risk categories for equine exposure to trypanosomiasis.

Exposure risk to equines to trypanosomiasis	Definition
No data	No data available
Negligible	No reported disease outbreak AND prevalence estimated at 0% on surveillance
Low	Single report of outbreak Point prevalence estimated 0%–5% WOAH reported outbreak
Medium	One to five reported outbreaks Point prevalence estimated 5%–10% WOAH reported disease present limited to one or more zones
High	More than five reported outbreaks Point prevalence estimated at >10% WOAH reported disease present

### 2.3 Methods for study question 2: Morbidity and mortality

This aimed to answer the question ‘In equines is trypanosomiasis infection a significant contributor to global morbidity and mortality?’

Studies were selected describing the number of animals with specific clinical signs of trypanosomiasis (per agreed case definition) in a screened population or outbreak.

#### 2.3.1 Critical assessment of methodological quality

Quality of evidence was assessed using GRADE^32^ (‘very low’, ‘low’, ‘moderate’ or ‘high’) and used to categorise outcome statements as having ‘strong’ or ‘weak’ evidence. Heterogeneity (clinical, methodological, statistical) was assessed qualitatively for each species of *Trypanosoma* sp. and/or quantitatively where meta‐analysis was performed.

#### 2.3.2 Descriptive data extracted from included studies

Data quality was anticipated to be low; therefore, qualitative and quantitative information were extracted. Data were extracted pertaining to the number of confirmed cases in the sampled population, the number of symptomatic equines (morbidity), the frequency of specific clinical signs, and the number of deaths (mortality). Cases that were positive on the diagnostic used but not presenting with clinical signs were recorded as asymptomatic (at time of evaluation). The outcome variables were morbidity and mortality. The death‐to‐case ratio was calculated when data permitted. Descriptive statistics for included studies were summarised in table format focused upon the comparison of outcome variables.

The data were sub‐categorised by reported *Trypanosoma* sp. and as ‘outbreak’ or ‘endemic disease’. An outbreak was defined as a report of an occurrence of cases linked by time, place or exposure where the observed number of cases exceeds the number expected for that location or time. Endemic disease was defined as disease regularly found within a specific population or geographic area and an expected level of occurrence. Four outcomes were reported for each of these sub‐categories where available in addition to the descriptive data using meta‐analyses of extracted proportion data using an inverse variance method with a random effects model (with Hartung‐Knapp adjustment) and logit transformation. For individual studies Clopper‐Pearson confidence intervals were calculated. *τ* ^2^ (using a DerSimonian‐Laird estimator) and *I* ^2^ (based on Q) were calculated as quantitative measures of heterogeneity. The results were visualised in forest plots. Analyses were performed using ‘metafor’^41^ and ‘meta’^42^ in R studio.^43^ R script is available in supplementary data (Data S3). The four outcomes were:

1. Infection rate (number of animals positive by diagnostics/number of animals with potential exposure to disease).
2. Morbidity (number of animals symptomatic/number of animals positive by diagnostics)
3. Mortality (number of animals died/number of animals with potential exposure to disease).
4. Death to case ratios (number of animals died/number of confirmed cases of disease).

## 3 RESULTS

### 3.1 General results: Descriptive data of literature

The complete extracted data can be accessed in Table S1. A PRISMA flow diagram illustrates the selection of eligible articles for study inclusion (Figure S1).

From an initial 10,491 manuscripts, 205 were included. The author institutions comprised 68% (139/205) from the studied country, 30% (62/205) a cross‐nationality collaborative team, and 2% (4/205) from a country outside of the country the manuscript reported on. Commonly (41%; 84/205) funding was not mentioned. External funding was referenced in 18% (36/205) and internal institutional funding in 28% (58/205). Ethical approval was documented in 28% (57/205). The manuscripts were predominantly of exploratory design (98%; 200/205) rather than confirmatory (2%; 5/205). As such, only 2% (5/205) stated a hypothesis. Sixty‐two percent (128/205) defined the aims and objectives of the manuscript.

### 3.2 Results for study question 1: Prevalence

#### 3.2.1 Demographics of data sources

##### Search outcome

**FIGURE 3:**
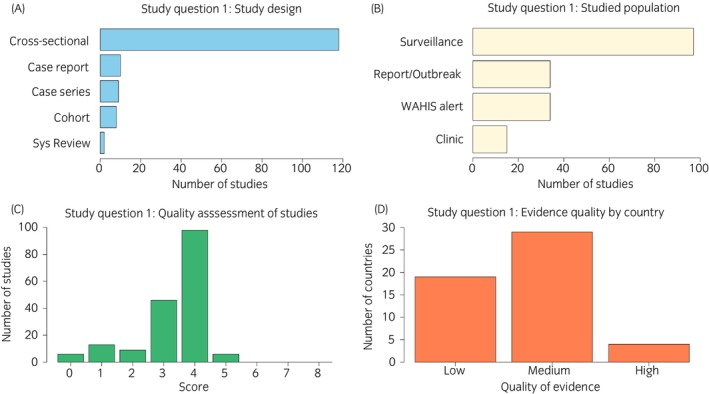
Barplots showing descriptive data of included studies relating to equine trypanosomiasis' prevalence and disease distribution. (A) Study design. (B) Types of populations. (C) Quality assessment scores. (D) Assessed evidence quality by country.

One‐hundred and forty‐seven of the 205 papers (72%) met the inclusion criteria for ‘Study question 1—Prevalence’. The most commonly reported was a descriptive, cross‐sectional survey (117/147; 80%), followed by case report (10/147; 7%), case series (9/147; 6%), descriptive, cohort (8/147; 5%) and systematic review (3/147; 2%). Descriptive, cross‐sectional surveys were commonly combined with diagnostic comparison or development (46/117; 40%) (Figure 3).

The three systematic reviews^46^, ^47^, ^48^ were checked for additional primary sources and used to provide evidence for data gaps and evidence of disease in other species within countries lacking data for equines. The selected studies and extracted data including FAO data on equine populations, quality of evidence assessment (study level and country level) and estimated point prevalence of countries with 95% confidence intervals are reported as supplementary data (Table S3; Data S2). Studies included were from Algeria,^49^, ^50^ Argentina,^51^, ^52^, ^53^, ^54^, ^55^ Brazil,^56^, ^57^, ^58^, ^59^, ^60^, ^61^, ^62^, ^63^, ^64^, ^65^, ^66^, ^67^, ^68^, ^69^, ^70^ Burkina Faso,^71^, ^72^ Chad,^73^ China,^74^ Columbia,^75^ Egypt,^76^, ^77^, ^78^, ^79^ Ethiopia,^8^, ^80^, ^81^, ^82^, ^83^, ^84^, ^85^, ^86^, ^87^, ^88^, ^89^, ^90^, ^91^, ^92^, ^93^, ^94^, ^95^ Gambia,^7^, ^96^, ^97^, ^98^, ^99^, ^100^, ^101^ Ghana,^102^ Greece,^103^ India,^104^, ^105^, ^106^, ^107^, ^108^, ^109^, ^110^, ^111^, ^112^, ^113^, ^114^, ^115^, ^116^, ^117^, ^118^, ^119^, ^120^, ^121^, ^122^, ^123^, ^124^, ^125^, ^126^, ^127^, ^128^, ^129^, ^130^ Indonesia,^131^, ^132^ Israel,^133^, ^134^ Italy,^135^, ^136^, ^137^, ^138^ Jordan,^139^ Kazakhstan,^140^ Kenya,^141^ Malaysia,^142^, ^143^ Mongolia,^144^, ^145^, ^146^, ^147^, ^148^ Namibia,^149^ The Netherlands,^47^, ^138^ Nigeria,^150^, ^151^, ^152^, ^153^, ^154^, ^155^, ^156^, ^157^ Pakistan,^158^, ^159^, ^160^, ^161^, ^162^, ^163^, ^164^, ^165^, ^166^, ^167^, ^168^, ^169^, ^170^ Palestine,^171^ Papua New Guinea,^172^ Paraguay,^173^ Philippines,^174^ Saudi Arabia,^175^ Senegal,^176^, ^177^ Spain,^178^, ^179^ Sudan,^10^, ^180^, ^181^, ^182^ Tanzania,^183^ Thailand,^184^, ^185^ Turkey,^186^ Uganda,^187^ United Arab Emirates^188^ and Venezuela.^189^

The manuscripts recorded 34 disease reports (which included reports of 22 disease outbreaks), 97 surveillance studies, and 15 clinic‐based surveillance data reports. Thirty‐four equine trypanosomiasis disease alerts were retrieved from WOAH, WAHIS (Figure 3).

##### Quality assessment of data sources

The individual studies were graded between zero and five out of eight (median 4/8) for their quality of evidence (Table S3). The most common concerns were absence of power calculation (120/147; 82%) and no mention of random sampling (115/147; 78%). For country reports from WOAH, WAHIS^34^ the quality of evidence was arbitrarily set as medium (4/8) since no information was available on sample size, diagnostic methods, or sampled population. Qualitatively, heterogeneity was high. The studies used a wide range of diagnostics; sampling techniques were variable, and sampling frames reflected different geographical regions and seasons of the year. The majority of studies reported positive results (139/147; 95%) introducing probable publication bias. This assessment was not used to exclude studies as a primary goal was to collate data from the maximum geographical area.

#### 3.2.2 Geographical distribution of reported *Trypanosoma* sp. in equines

**FIGURE 4:**
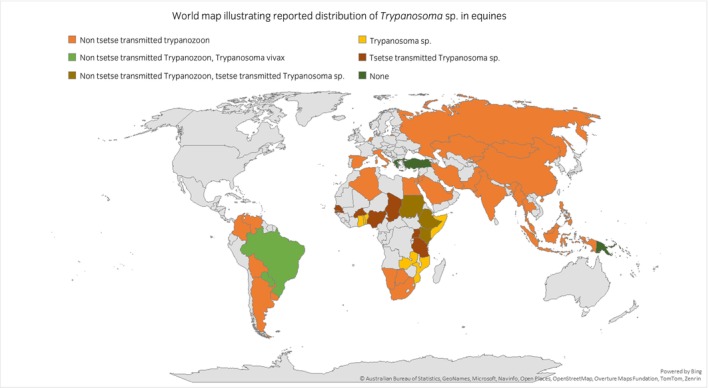
World map illustrating reported *Trypanosoma* sp. in equines. *Trypanosoma equiperdum* and *Trypanosoma evansi* are reported as Trypanozoon due to the challenges of differentiating these pathogens solely using diagnostics. *Trypanosoma brucei*, *Trypanosoma congolense* and *Trypanosoma vivax* are collectively reported as tsetse transmitted *Trypanosoma* spp. with the exception of *Trypanosoma vivax* in South America where there are no tsetse flies and the vector is solely biting flies.

Reported geographical distribution was wide. Only three of the 52 countries for which data were available reported no evidence of *Trypanosoma* sp. *T. evansi* was the most commonly reported *Trypanosoma* sp. by country (26/52 countries) (Table 4; Figure 4; data presented in Table S4).

**TABLE 4:** The number of countries in which different species of trypanosomes have been detected in equines.

*Trypanosoma* sp.	Number of countries (*n* = 52)
*Trypanosoma evansi*	26
*Trypanosoma equiperdum*	13
*Trypanosoma brucei*	7
*Trypanosoma congolense*	9
*Trypanosoma vivax*	11
*Trypanosoma* sp. (microscopic diagnosis)	11
None	3

#### 3.2.3 Comparison of HIC and LMIC risk of exposure

The majority of the countries for which data were available were categorised as LMIC (44/52; 85%) representing 34% of LMIC countries (43/126) for which the majority were reporting disease presence (42/44; 95%). Data were available for eight HICs, representing 10% of HICs (8/80). The majority of these HICs reported disease presence (7/8; 88%). Based on these data, LMICs were over‐represented in reporting equine disease (42/126, 33% vs. 7/80, 9%; *p* < 0.001 OR 5.2 2.1–14.5) (data presented in Table S4).

#### 3.2.4 Country level prevalence estimation

**FIGURE 5:**
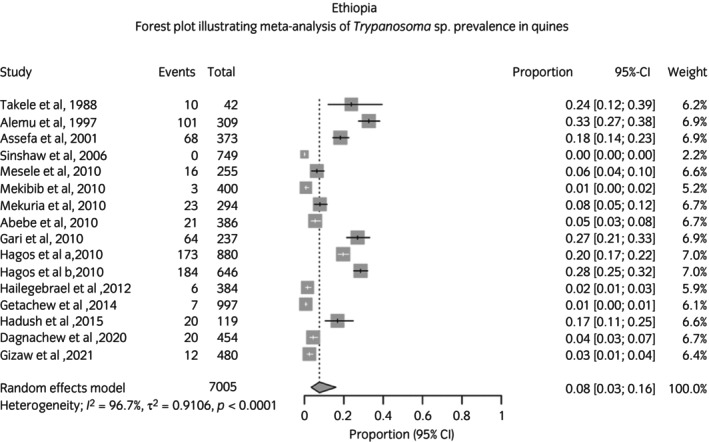
Forest plot illustrating meta‐analysis of *Trypanosoma* sp. prevalence in equines in Ethiopia. The remainder of the forest plots are available in Data S2.

For 37 countries, country‐level prevalence was estimated. The Philippines, Gambia, Argentina, Paraguay, and Chad were the countries with the highest recorded pooled prevalence (Tables 5 and S3). Forest plots for each country with available data are available in supplementary data (Data S2). An example forest plot illustrating the meta‐analysis of prevalence literature for Ethiopia is shown in Figure 5. Quantitative heterogeneity of studies was high at the country level.

**TABLE 5:** Summary of estimated country level prevalence of equine trypanosomiasis (including 95% confidence intervals).

Country	Estimated prevalence (%) (95% CI)	Quality of evidence
Philippines	91 (78–97)	Low
Gambia	43 (14–77)	Medium
Argentina	39 (0–99)	Medium
Paraguay	36 (32–39)	Medium
Chad	35 (30–41)	Medium
Uganda	32 (22–45)	Low
Egypt	19 (1–89)	Medium
Kazakhstan	17 (11–24)	Low
Palestine	16 (8–26)	Low
Algeria	15 (0–100)	Medium
Malaysia	14 (11–17)	Medium
Brazil	13 (1–61)	Medium
Sudan	11 (1–68)	Medium
India	10 (6–17)	Medium
Pakistan	9 (5–14)	Medium
Ethiopia	8 (3–16)	High
Namibia	8 (7–9)	Medium
Venezuela	7 (4–13)	Medium
Jordan	7 (3–12)	Low
Senegal	7 (3–15)	Medium
Burkina Faso	6 (5–6)	High
Indonesia	5 (0–100)	Medium
Israel	5 (3–7)	Medium
Nigeria	5 (1–13)	Medium
Mongolia	5 (2–10)	High
United Arab Emirates	5 (1–14)	Low
Ghana	3 (0–12)	Low
Saudi Arabia	3 (2–5)	Medium
Spain	3 (2–4)	Medium
Columbia	2 (1–3)	Medium
Thailand	0 (0–1)	Medium
China	0 (0.00–0.01)	Low
Italy	0.00 (0.00–0.00)	Medium
Greece	0 (0.00–0.00)	Medium
Papua New Guinea	0 (0.00–0.00)	Low
Turkey	0 (0.00–0.01)	Low

#### 3.2.5 Summated prevalence and risk assessment for equines in countries with data available

**FIGURE 6:**
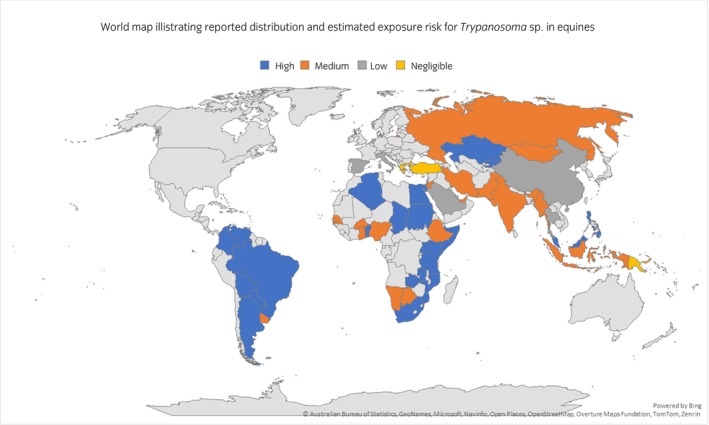
World map visualising by country estimated risk of exposure to trypanosomiasis as negligible, low, medium or high (defined in Table 3). Countries for which no data were available are coloured in grey.

An estimate of 12% (5%–33%; 36 countries, exposed 8,289,526; population 69,075,069) was made for the summated prevalence of equine trypanosomiasis in countries for which quantitative data were available (*n* = 37; data presented in Table S3). All data (prevalence, outbreak, WAHIS) were used for the risk assessment which permitted evaluation of 52 countries populated with approximately 75.7 million equines.^39^ Approximately 67.5 million of these equines were estimated to live in a medium or high risk country (56% of world equine population) (Table 6; Figure 6; data presented in Table S3). For the remaining approximately 45.8 million equines, there were no data available (38%).

**TABLE 6:** The estimated number of equines residing in different risk levels (defined in Table 3) for equine trypanosomiasis.

Risk level	Number of equines residing in risk level	Percentage of world equine population
Negligible	248,373	0.2
Low	7,849,155	6.5
Medium	32,389,319	26.7
High	35,207,282	29.0
No data	45,826,656	37.7
Total	121,520,785	100.0

#### 3.2.6 Risk assessment for countries with missing data

There were no data available pertaining to the presence or absence of *Trypanosoma* spp. in the equine populations of most countries (153/206; 74%). There is no disease‐free status awarded by WOAH for trypanosomiasis.^190^ When collating this data, an incongruence was noted between the small number of countries that declare no disease to WOAH and those where reports have been published in the peer‐reviewed literature. The regions of missing data can be divided into areas where prior knowledge would expect disease not to be present and regions where disease would be expected to be present.

##### Disease not expected to be present

*North America, Australia, New Zealand, and Europe (excluding Russia)*:

There are open source equine disease surveillance systems^35^, ^36^, ^37^, ^38^ in place in several of these countries. These are high‐income countries with predominantly performance and pleasure horses, large amounts of international travel, and national/international organisations implementing protocols for disease screening.^47^ Disease control and surveillance measures for trypanosomiasis (WAHIS, WOAH) are reported in approximately two out of three countries (data reported in Table S5). In the study period, there have been two outbreaks in these regions (Italy, Spain) and a single case (Netherlands) that have been reported in considerable detail^47^, ^135^, ^136^, ^137^, ^138^, ^178^, ^179^ supporting the functionality of the systems in place. There is a large amount of opportunity to detect disease. These regions are considered to be highly likely to be at negligible risk of disease.

##### Disease expected to be present

*Africa*:

All of the African countries (19/19) where data are available report the presence of equine trypanosomiasis at medium or high risk. Two of the systematic reviews which were included in this study provide evidence of a wider distribution in other susceptible species across this continent of *T. vivax* ^48^ and *T. evansi*.^46^ The tsetse fly vector is present across a large proportion of the continent.^191^ Most (44/54; 81%) countries in Africa have recorded populations of equines.^39^ All the African countries are lower or middle‐income countries^40^ with predominantly working equine populations. No open‐source disease surveillance systems have been identified, suggesting lower opportunity to detect disease. Disease control and surveillance measures (WAHIS, WOAH) are reported by a minority of countries (Table S5).

These regions are therefore considered to be highly likely to have equine trypanosomiasis at medium or high risk levels. Further surveillance studies are a priority for countries where there are no data in this region.

*Asia (including Russia)*:

For the countries with data available, the majority report the presence of equine trypanosomiasis (15/16) at low, medium, or high risk. One of the systematic reviews which was included in this study provides evidence of a wider distribution in other susceptible species across this continent of *T. evansi*.^46^ In Asia, 43/49 (88%) of countries have recorded populations of equines.^39^ The majority of countries in Asia are lower or middle‐income countries^40^ with predominantly working equine populations. No open‐source disease surveillance systems have been identified, suggesting a lower opportunity to detect disease. Disease control and surveillance measures (WAHIS, WOAH) are reported in less than half of the countries (Table S5).

These regions are therefore considered to be highly likely to have equine trypanosomiasis at medium risk levels. Further surveillance studies are a priority for countries where there are no data in this region.

*Central and South America*:

All of the Central and South American countries for which data were available report the presence of equine trypanosomiasis (7/7) at medium or high risk levels. Two of the systematic reviews which were included in this study provide evidence of a wider distribution in other susceptible species across this continent of *T. vivax* ^48^ and *T. evansi*.^46^ All countries in Central and South America have recorded populations of equines.^39^ Only one country is a higher income country (Chile)^40^ with predominantly working equine populations. No open source disease surveillance systems have been identified, suggesting lower opportunity to detect disease. Disease control and surveillance measures (WAHIS, WOAH) are reported in approximately 2/3 of countries (Table S5).

These regions are therefore considered to be highly likely to have equine trypanosomiasis at medium or high risk levels. Further surveillance studies are a priority for countries where there are no data in this region.

### 3.3 Results for study question 2: ‘Morbidity and mortality’

#### 3.3.1 Search outcome

**FIGURE 7:**
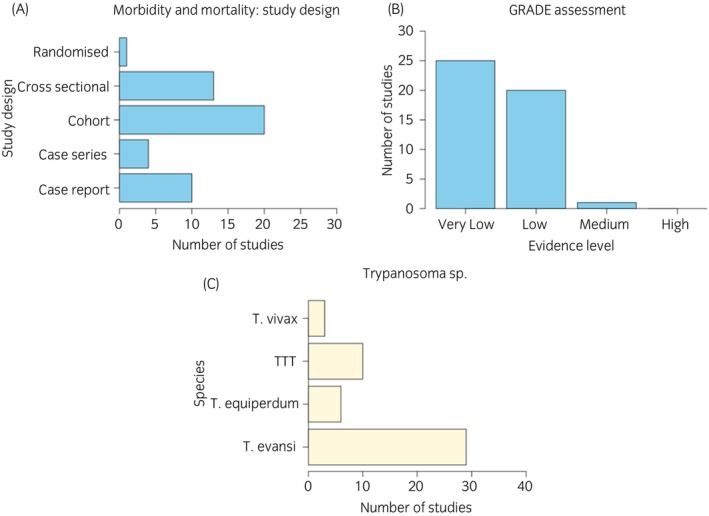
Barplots illustrating descriptive data of included studies on morbidity and mortality related to equine trypanosomiasis. (A) Study design. (B) GRADE assessment. (C) *Trypanosoma* sp. described. TTT, tsetse transmitted trypanosomiasis.

Forty‐eight of the 205 manuscripts (23%) reviewed met the criteria. There was one randomised noninferiority trial, 20 cohort studies, 4 case series, 13 cross‐sectional studies, and 10 case reports (Figure 7).

Twenty‐nine studies related to *T. evansi* (describing 11 outbreaks, 16 endemic disease), six to *T. equiperdum* (three outbreaks, three endemic disease), ten to tsetse transmitted trypanosomiasis (mixed infections) (one outbreak, nine endemic disease) and three to *T. vivax* (two outbreaks, one endemic disease) (Figure 7).

The quality of evidence was assessed as ‘moderate’ (*n* = 1), ‘low’ (*n* = 20) or ‘very low’ (*n* = 25) (Figure 7). On qualitative assessment, heterogeneity was high (clinical, methodological and statistical) and therefore re‐evaluated for each subcategory to determine whether data assimilation were useful. Meta‐analyses and forest plots by *Trypanosoma* sp. and disease characteristics (infection rate, morbidity, mortality and death to case ratio) are available in Data S3. Trypanocidal treatment was given in some studies and will impact upon the outcome variables. The effect size of this intervention is unknown as the data quality does not permit even qualitative assessment. It was often unclear what drug, dose and route of administration had been used or at what time point in the disease course the drug was given.

#### 3.3.2 Trypanosoma evansi

##### Reported disease outbreaks

There were 13 studies (nine cohort studies, two case series and two case reports) involving outbreaks of *T. evansi*. Geographical representation was from six countries on three continents (Brazil (*n* = 5), Israel (*n* = 2), Thailand (*n* = 2), India (*n* = 2), Malaysia (*n* = 1), Spain (*n* = 1)). Quality of evidence^32^ was assessed as ‘very low’ (*n* = 9) or ‘low’ (*n* = 4). Heterogeneity originated from study design, diagnostics, follow‐up period, treatment and reporting of clinical data. Extracted data are summarised in Table 7.

**TABLE 7:** Summary of clinical reports of *Trypanosoma evansi* outbreaks.

Study	Study design	Infection rate	Morbidity	Mortality and death to case ratio	Asymptomatic animals?	Timeframe of outbreak and follow‐up	Grade
Zanette et al 2008^67^	Cohort	5/13 (38%)	4/13 (31%)	0/13 (0%)	1/5 (20%)	No details	Very low
Berlin et al 2009, 2010^133^, ^200^	Case report; Cohort	9/20 (45%)	1/20 (5%) (neurological and haemolymphatic clinical signs)	1/20 (5%) DCR: 1/1 (100%)	8/20 (40%) Positive on diagnostic no clinical signs	~4 months clinical signs‐ death 2 years follow‐up	Very low Low
Tuntasuvan et al 2003^184^	Cohort	57/57 (100%)	57/57 (100%) 42% pregnant mares aborted/stillbirth	20/57 (1/10 mules; 19/47 horses); 35% DCR: 35%	No	~2 months clinical onset‐ resolution/death. No follow‐up for clinically resolved cases	Very low
Yadav et al 2014^130^	Cohort	9/30 (30%)	1/30 (3%)	1/30 (3%) DCR: 1/9 (11%)	8/30 (27%) Very early intervention with trypanocide	Disease onset within 6 days of index case 6 months (↓ Ab positive)	Very low
Camoin et al 2011^185^	Cohort	26/29 (90%)	26/29 (90%) (neuro signs 2/29; haemolymphatic 26/29)	20/29 (69%) including 2/2 neuro signs DCR: 20/26 (77%)	No. 2/29 (7%) remained uninfected	~1 month outbreak (infected local cattle) No details of follow‐up	Low
Reck et al 2020^66^	Cohort	6/30 (20%)	6/30 (20%) (neuro signs 3/6; haemolymphatic signs 6/6)	0/30 (0%) DCR: 0/6 (0%)	Post treatment 4/6 asymptomatic	~1 month of outbreak cases 3 months follow‐up	Very low
Da Silva et al 2016^65^	Cohort	256/826 (31%)	256/826 (31%)	223/826 (27%) DCR: 223/256 (87%)	Cases without clinical signs not tested	~1 month outbreak (*n* = 2) No details of follow‐up	Very low
Tamarit et al 2010^179^	Cohort	8/92 (9%)	No details	0/92 (0%) DCR: 0/8 (0%)	No details	Index case (camel) at premises 18 months before onset of clinical signs ~2 months follow‐up	Low
Ranjithkumar et al 2014^199^	Case series	14 horses	14 horses (total population unknown) 4/14 (29%): neurological disease (multifocal lesions localised to forebrain/brain stem and/or spinal cord) post treatment (27–151 days). NB. CSF PCR can be −ve in histologically confirmed disease	Total population unknown DCR: 3/14 (21%) 3/4 with neurological disease (disease course 2‐10 days)	Only symptomatic discussed.	Initial outbreak ~2 months 5 months follow‐up	Very low
Rajdi et al 2021^143^	Case report	1 horse	1/1 Tachycardia, fever, anaemia, poor body condition, oedema, urticaria on neck. Neurological signs (ataxia, weakness)	1/1	No	2 months clinical course until death	Very low
Rodrigues et al 2005; 2009^5^, ^68^	Case series; Cohort	205 horses (2 premises, part of larger outbreak)	66/80 (83%) (1 premises) 23 horses with clinical signs reported in detail Weight loss despite good appetite, lethargy, HL muscle atrophy/weakness, pale mucous membranes, anaemia and leukocytosis (lymphocytosis). Neurological signs (incoordination/instability of HL) Encephalitic signs (*n* = 9)	108/205 (53%) (2 premises) DCR: 52/66 (79%) of one cohort 9/9 (100%) with encephalitis died/euthanised (Marked ataxia, blindness, head tilt, circling, hyperexcitability, obtundity, proprioceptive deficits, head pressing, paddling movements)	10/62 (16%) Ab +ve serum samples from clinically healthy horses (random) 4/5 (80%) Ab +ve in neurological cases	Encephalitis: clinical course 2–20 days	Very low; Low
*Meta‐analyses and descriptive summations*		*Infection rate: 42% (95% CI 14–76)*	*Morbidity: 47% (95% CI 13–85)*	*Mortality: 23% (95% CI 7–54)* *DCM:45% (95% CI 20–73)*	*Not actively looked for. often only symptomatic tested* *27 animals which fulfil criteria*	*Length of outbreaks range ~1–4 months*	

**FIGURE 8:**
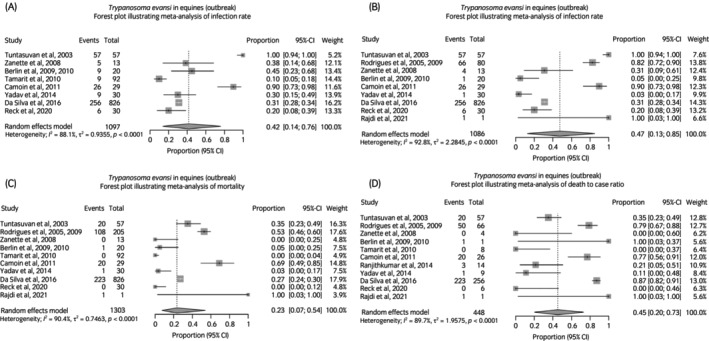
Forest plots illustrating meta‐analyses for *Trypanosoma evansi* disease outbreak characteristics. (A) Infection rate. (B) Morbidity. (C) Mortality. (D) Death to case ratio.

For *T. evansi* outbreaks the combined infection rate (proportion; 95% CI) (excluding individual case reports;) on a premises was 42% (14–76) of equines, morbidity (number symptomatic in population premises) was 47% (13–85) and mortality (number that died on population premises) was 23% (7–54). Death to case ratio was 45% (20–73) (forest plots Figure 8). There were no quantitative data on presenting clinical signs or consistent staging of disease (haemolymphatic/ neurological). Reported disease outbreaks were consistent with acute onset clinical signs and relatively rapid disease progression through premises (range 1–4 months). There is weak evidence that *T. evansi* can cause abortion storms.^184^

##### Reported endemic disease

There were sixteen studies (three cohort studies, seven cross‐sectional and six case reports) which reported disease classified as endemic. Geographical representation was from four countries on two continents (Brazil (*n* = 2), India (*n* = 9), Paraguay (*n* = 2), Pakistan (*n* = 3)).

Quality of evidence^32^ was assessed as ‘very low’ (*n* = 10) or ‘low’ (*n* = 6). Extracted data are summarised in Table 8.

**TABLE 8:** Summary of clinical reports of endemic *Trypanosoma evansi*.

Study	Study design	Infection rate	Morbidity	Mortality and death to case ratio	Asymptomatic animals?	Timeframe of outbreak and follow‐up	Grade
Waheed et al 1998; Waheed et al 2003; Gondal and Ahmad 2010^164^, ^170^, ^202^	Cross‐sectional, longitudinal	2910/73497 (clinic/hospital population)	2910/73497 Positive cases 52/85 (61%) pyrexic. Oedema rare. Superficial lymphadenopathy associated with neurological signs seen in advanced cases.	116/73497 (0.2%) DCR: 116/2910 (4%)	No data. Clinical signs influenced decision to test.	Follow‐up assumed short (mobile clinic/hospital based regional intervention).	Very low
Bhatt et al 2010^106^	Case report	1 horse	1/1 Clinical signs (fever, anaemia, poor body condition, dehydration, rough hair coat, no neurological signs).	0/1	No	Symptomatic for several days prior to presentation. 10 days until resolution of clinical signs.	Very low
Shaikh et al 2016^104^	Case report	1 horse	1/1 Clinical signs (tachycardia, fever, anaemia, poor body condition, oedema, haematuria, no neurological signs).	0/1	No	Symptomatic for ~1 month prior to presentation. 15 days until resolution of clinical signs.	Very low
Bharkad et al 2005^107^	Case report	1 horse	1/1 Clinical signs (tachycardia, fever, anaemia, poor body condition, oedema, neurological signs (recumbent, weakness))	1/1	No	Symptomatic for ~1 month prior to presentation. Died third day treatment.	Very low
Laha and Sasmal 2008^110^	Cohort	14/102 (14%)	14/102 (14%) 14/14 fever; 4/14 anaemia, 10/14 poor body condition, 4/14 oedema, 2/14 neurological signs.	6/102 (6%) (inc. 2/2 neuro signs) DCR: 6/14 (42%)	No	~4 month period of follow‐up.	Very low
Tehseen et al 2017^159^	Cross‐sectional	54/375 (14%)	No details of number with clinical signs. Emaciated animals OR = 17.5 (95%CI 8.2–37.4) for Surra diagnosis.	N/A no period of follow‐up.	No clinical details	0	Low
Kumar et al 2020^126^	Case report	1 mule	1/1 Tachycardia, fever, anaemia, poor body condition score, anorexia, depression, no oedema, lymphadenopathy, petechial haemorrhages. Neurological signs (mild ataxia, HL wobbling gait).	0/1	No	Duration of clinical signs unknown. 6 months follow‐up.	Very low
Saqib et al 1998^167^	Case report	1 donkey	1/1 Tachycardia, fever, anaemia, poor body condition, no oedema, depressed, tachypnoea. Neurological signs (ataxia).	0/1	No	3 months duration of clinical signs. 3 days follow‐up post treatment.	Very low
Bhardwaj et al 2007^117^	Case report	1 horse	1/1 Tachycardia, fever, anaemia, petechial haemorrhages, nasal/ocular discharge, frequent urination. Neurological signs (staggering gait).	0/1	No	Duration of clinical signs unknown. 5 months follow‐up (live foal)	Very low
Parreira et al 2016^59^	Cohort	51/105 (49%) Ab +ve	0/105 (0%)	0/105 (0%) 0/51 (0%)	51/105 (49%)	15 month period of study	Low
Herrera et al 2004^60^	Cohort	236/321 (74%) 31/321 (10%) parasitaemic	Anaemia correlated to parasitaemia. Mean population values 30–33%. No neurological signs noted. Full clinical examination not documented.	0/321 (0%)	Not clear, but clinical signs if present inferred to be mild, no overt neurological signs.	No follow‐up.	Low
Suganuma et al 2022; Yamazaki et al 2022^173^, ^192^	Cross‐sectional	68/739 (9%)	Horses were ‘apparently healthy’, no clinical data presented. Mixed infections (with *T. vivax*) caused mild reduction in mean haematocrit. No significant difference with *Trypanozoon* infections only.	N/A No follow‐up.	Animals screened were apparently healthy (no clinical data presented).	No follow‐up.	Low
Pal et al 2021^127^	Cross‐sectional	55/285 (19%) (inc. 65 donkeys)	No clinical data. Mean packed cell volume 20% vs. 34% in uninfected.	N/A No follow up.	No data.	No follow up.	Low

For *T. evansi* reports in endemic regions, there is clear methodical heterogeneity in the study populations which negates any value in summary data (screening healthy populations vs. symptomatic hospital/clinic populations). Clinical data are sparse and the majority of studies have no available follow‐up data, reducing the value of quoted mortality figures. There is strong evidence for the common association of *T. evansi* with anaemia, fever, poor body condition score, and neurological abnormalities but not in all cases (Table 8).

There is weak evidence for the presence of apparently asymptomatic/minimally symptomatic equines suggested by three study populations^59^, ^60^, ^173^, ^192^ although detailed clinical data and follow up are not available for analysis.

#### 3.3.3 Trypanosoma equiperdum

##### Reported disease outbreaks

There were three studies involving outbreaks of *T. equiperdum* (one cohort, one case series and one case report) pertaining to the same outbreak (Italy). Quality of evidence^32^ was assessed as ‘very low’ (*n* = 2) or ‘low’ (*n* = 1). Extracted data are summarised in Table 9.

**TABLE 9:** Summary of clinical reports of *Trypanosoma equiperdum* outbreaks.

Study	Study design	Infection rate	Morbidity	Mortality and death to case ratio	Asymptomatic animals?	Timeframe of outbreak and follow‐up	Grade
Calistri et al 2013; Vulpiani et al 2012; Scacchia et al 2011^136^, ^137^, ^138^	Cohort; Case series; Case report	By premises 20/168 (12%, 95% CI 7–18)	5/168 (3%) of equines on premises 5/20 (25%; 95% CI 9–49) of diagnosed cases **Index case** Poor body condition, anaemia, perineal depigmentation, oedematous skin plaque, peripheral lymphadenopathy. Hindlimb ataxia. **Clinical evaluations of *n* = 6**: **Stage 1 (genital)**: Weight loss (6/6); Sheath/ vulva oedema (2/6) **Stage 2 (cutaneous)**: Wheals/Plaques (3/6); Oedema: (mammary (3/4); limb (4/6); ventral (3/6)) **Stage 3 (neurological)**: Ataxia (2/6); Facial nerve paralysis (lower lip) (2/6); Progression of anaemia (2/6 did not become anaemic); Joint effusions (4/6) None presented with fever	20/20 (100%) (euthanasia protocol)	15/20 (75%) No longitudinal follow‐up	Variable period of infection (up to ~1 year before diagnosis) Symptomatic equines observed prior to euthanasia (15 days to 6 months)	Low; Very low; Very low

For *T. equiperdum* the single outbreak, proactively managed in a naïve population, provides some limited data and an example of the approach to countrywide disease eradication. The infection rate of the outbreak was 12% (95% CI 7–18) and 25% (9–49) of cases were symptomatic at the time of diagnosis. There are no data pertaining to the use of these animals for breeding and what proportion of those exposed were identified cases (symptomatic or not). There was a relatively long period of time prior to the identification of the index case and a low total number of cases identified.

Clinical signs in the cases reported were consistent with a mixture of acute and chronic disease. The diagnostic criteria used to determine a case underline the complexity of disease identification. Observed disease course was up to six months. There was no attempt at treatment and all cases were euthanised per the country's eradication plan.

##### Reported endemic disease

There were three studies (two cross‐sectional studies, one case report) which reported disease classified as endemic. Geographical representation was from two countries on two continents (Ethiopia (*n* = 1), Mongolia (*n* = 2)). Quality of evidence^32^ was assessed as ‘very low’ (*n* = 1) or ‘low’ (*n* = 2). Extracted data are summarised in Table 10.

**TABLE 10:** Summary of clinical reports of endemic *Trypanosoma equiperdum*.

Study	Study design	Infection rate	Morbidity	Mortality and death to case ratio	Asymptomatic animals?	Timeframe of outbreak and follow‐up	Grade
Alemu et al 1997^83^	Cross‐sectional	101/309 (33%)	66/309 (21%) of sampled population. 66/101 (65%) of serologically positive population. No quantitative clinical data. Poor body condition, oedema of genitalia, conjunctivitis and neurological signs (HL wide gait, recumbency, paraplegia, paralysis and death) described.	No follow‐up	35/101 (35%) report no clinical signs but detailed clinical data and follow‐up not provided. Many had received trypanocidal treatment.	No follow‐up	Low
Davkharbayar et al 2020^144^	Case report	1 horse	1/1 Poor body condition, oedema (testicles, sheath, scrotum), depigmentation of penis, smegma accumulation, no skin plaques. No ataxia, mild unilateral facial nerve paralysis.	0/1	No	2.5 year follow‐up including repeat diagnostics	Very low
Clausen et al 2003^145^	Cross‐sectional	75/1122 (7%)	Unknown Poor body condition correlated with positive serological status. Abortion in mares: ×2 OR for seropositivity.	No details	No detailed clinical data.	No follow‐up	Low

For *T. equiperdum* reports in endemic regions, there is methodological heterogeneity in the study designs and reporting of clinical data that prevents summation of findings. There is no information on how breeding is controlled within the different sampled populations. Clinical data are sparse, and the two cross‐sectional studies have no follow‐up data and so provide no data on mortality. There is weak evidence for the association of poor body condition score and abortion in mares with *T. equiperdum* infection.^145^

There is weak evidence for the presence of apparently asymptomatic/minimally symptomatic equines^83^ although no detailed clinical data or follow‐up are available. The percentage of reported asymptomatic equines in that endemic population is lower than in a reported outbreak (35% vs. 75%) although the diagnostic approach differed. The case report^144^ provides an example of the detail of clinical and diagnostic data that would be useful for the larger studies.

#### 3.3.4 Tsetse transmitted trypanosomiasis

##### Reported disease outbreaks

There was one study (one cohort) involving an outbreak of Tsetse transmitted trypanosomiasis from Kenya.^141^ Quality of evidence^32^ was assessed as ‘very low’ (*n* = 1). Extracted data are summarised in Table 11.

**TABLE 11:** Summary of clinical reports of tsetse transmitted trypanosomiasis outbreaks.

Study	Study design	Infection rate	Morbidity	Mortality and death to case ratio	Asymptomatic animals?	Timeframe of outbreak and follow‐up	Grade
Kihurani et al 1994^141^	Cohort	16/35 (46%; 95% CI 29–63)	16/35 (46%; 95% CI 29–63) Majority tachycardic, febrile, poor body condition. Oedema and corneal opacity occ. noted. 16/16 anaemic	2/35 (6%; 95% CI 1–19) DCR: 2/16 (12%; 2–38)	Only testing symptomatic	6 symptomatic in first 6 months Follow‐up for further 2 months	Very low

For tsetse transmitted trypanosomiasis, a single documented outbreak provides some limited data (although instigation of a post outbreak preventative trypanocide protocol suggests the region could be endemic). In this report the infection rate and morbidity were 46% (29–63), mortality 6% (1–19) and death to case ratio 12% (2–38). Anaemia at presentation was a consistent clinical finding.

##### Reported endemic disease

There were nine studies (one randomised noninferiority trial, four cohort studies, three cross‐sectional and one case series) which reported Tsetse transmitted trypanosomiasis classified as endemic. Geographical representation was from three countries within Africa (Gambia (*n* = 6), Ethiopia (*n* = 2), Tanzania (*n* = 1)). Quality of evidence^32^ was assessed as ‘moderate’ (*n* = 1), ‘low’ (*n* = 6) and ‘very low’ (*n* = 2). Extracted data are summarised in Table 12.

**TABLE 12:** Summary of clinical reports of endemic tsetse‐transmitted trypanosomiasis.

Study	Study design	Infection rate	Morbidity	Mortality and death to case ratio	Asymptomatic animals?	Timeframe of outbreak and follow‐up	Grade
Abebe et al 2010^203^	Cross‐sectional	21/386 (5%)	Low PCV and poor body condition score associated with positive infection status	No data	No	No follow‐up	Low
Raftery et al 2019, 2020^96^, ^193^	Randomised noninferiority trial, Cohort	162/247 (66%) 141 donkeys	Not recorded 247/641 (39%) symptoms 162/247 (66%) +ve PCR Tachycardia (125/162; 77%); fever (91/162; 56%); anaemia (157/162; 97%); poor body condition (110/162; 68%); oedema (6/162; 4%), quiet/dull demeanour (118/162; 73%). Abortion (21/92; 22%). (Equines with *neurological signs excluded*)	DCR: 1/162 (0.6%)	Increase in PCV post trypanocide administration in PCR negative group, consistent with low level infection (minimally symptomatic)	2 weeks	Moderate, low
Auty et al 2008^183^	Cohort	18 cases in 24 horses over 18 months (75%)	Not recorded. Only symptomatic tested. 14/18 (78%) haemolymphatic clinical signs; 4/18 neurological signs. Intensive clinical monitoring. All had pyrexia at presentation except 1 (17/18) likely a chronic case previously infected. Frequency of clinical signs: jaundice (11/18), depression (11/18), anorexia (8/18), aggression (1/18), headshaking (1/18), ataxia (4/18). Chronic cases developed anaemia, ataxia and weight loss.	4/24 (17%) DCR: 4/18 (22%) Mortality outcome associated with presence of ataxia, severe anaemia and weight loss.	No 18/18 (100%) symptomatic	Longitudinal study over 18 months	Low
Dhollander et al 2006^7^	Cross‐sectional	1214/2285 (53%)	Negative correlation between mean PCV and parasitaemia score (darkground microscopy). Clinical signs included mild to severe weight loss, mild to severe oedema, mild to severe anorexia, quiet demeanour to total paresis.	Point of care. No follow‐up.	No	No follow‐up.	Low
Savage et al 2021^101^	Cohort	162/323 (50%)	Not recorded 34/323 (11%) had neurological signs: ‐Donkeys: progressive cerebral dysfunction, cranial nerve deficits dysphagia, facial nerve paralysis; ‐Horses: progressive spinal ataxia. Neurological disease: BCS <2 (OR 11.4 (4.6–27.9)), fever (*p* = 0.008; OR 3.3 (1.3–9.5)), lower PCV (*p* = 0.024), tachycardia (p < 0.001) Positive serology for *T. brucei* 18/19 (95%) neurological equines vs. 21/35 (60%) no neuro signs (*p* = 0.009).	14/323 (4%) DCR: 14/162 (9%) Equines with neurological signs had 75% mortality where follow‐up available.	No data	No follow‐up for most equines.	Low
Dagnachew et al 2020^95^	Cohort	20/454 (4%) (434 donkeys; 20 mules)	Poor body condition OR 8 (3.8–10) for trypanosome infection vs. good body condition.	No follow‐up	No data	No follow‐up	Low
Sutton et al 2012^204^	Cross‐sectional (abstract)	35/101 (35%) (72 donkeys)	In screened apparently healthy population no significant difference in clinical parameters or body condition between infected or uninfected. Significant association between PCV and infection status.	No follow‐up	Equines presented apparently healthy supporting mild signs only if present.	No follow‐up	Very low
Kingston 2018^194^	Case series	N/A	N/A	13/13 (100%) (neuro cases)	Recruitment based on clinical and neurological abnormalities.	All recruited with neurological signs, survival up to 1 year.	Very low
*Meta‐analyses*		*Infection rate = 22% (95% CI 4–66) (n = 5)*		*Mortality = 8% (95% CI 0–100) (n = 2)* *DCT = 19% (95% CI 1–87%) (n = 3)*			

Four studies provided detailed data on clinical parameters of infected equines.^96^, ^101^, ^183^, ^193^ Two studies^101^, ^194^ documented in depth findings of neurological examinations of cases with CNS invasion.

The infection rate was 22% (95% CI 4–66), morbidity could not be extracted, mortality 8% (0–100) and death to case ratio 19% (1–87) (*n* = 8 not all with data for each figure; excluding the case series selecting for neurological disease). The single study^183^ with longer follow‐up (18 months) reported the highest mortality (17%).

There is strong evidence (one observational study but a low risk intervention with high potential benefit) in favour of recording twice daily rectal temperatures of equines in high challenge regions to detect fever (due to the association with acute onset disease and the improved outcomes with early treatment).^183^ There is strong evidence for the association of mortality with ataxia, weight loss/poor body condition score, and severe anaemia (Table 12). There is weak evidence for the presence of minimally symptomatic/ asymptomatic equines (Table 12).

#### 3.3.5 *Trypanosoma vivax* (non tsetse transmitted)

##### Reported disease outbreaks

There are two studies (two cohort) involving outbreaks of *T. vivax*. Geographical representation from two countries on two continents (Brazil (*n* = 1), Senegal (*n* = 1)). Quality of evidence^32^ was assessed as ‘very low’ (*n* = 1) or ‘low’ (*n* = 2). Extracted data are summarised in Table 13.

**TABLE 13:** Summary of clinical reports of *Trypanosoma vivax* outbreaks.

Study	Study design	Infection rate	Morbidity	Mortality and death to case ratio	Asymptomatic animals?	Timeframe of case identification and follow‐up	Grade
Da Silva et al 2011^61^	Cohort	6/12 (50%)	6/12 (50%) Clinical signs fever (*n* = 6), anaemia (*n* = 4), oedema (prepuce/vulva) (*n* = 6). No neurological signs. Post treatment clinical relapses 3×	3/12 (25%) DCR: 3/6 (50%) (2 euthanised)	No	6 months follow‐up	Very low
Dehoux et al 1996^177^	Cohort	20/49 (41%)	20/49 (41%) Common clinical signs: fever up to 41°C, anaemia, oedema.	5/49 (10%) DCR: 5/20 (25%)	Only tested symptomatic	Deaths occurred ~5 weeks after onset of clinical signs	Low
*Total*		*Infection rate = 43% (95% CI 10–83) (n = 2)*	*Infection rate = 43% (95% CI 10–83) (n = 2)*	*Mortality = 15% (95% CI 0%–99%) (n = 2)* *DCR = 32% (95% CI 0–100) (n = 2)*			

The median infection rate was 43% (95% CI 10–83), morbidity 43% (10–83), mortality 15% (0–100), DCR 32% (0–100) (*n* = 2). Clinical signs were haemolymphatic with no neurological signs documented.

##### Reported endemic disease

There was one study (one cross‐sectional) from Brazil which reported *T. vivax* classified as endemic. Quality of evidence^32^ was assessed as ‘low’ (*n* = 1). Extracted data are summarised in Table 14. There is weak evidence for the presence of asymptomatic infected donkeys.

**TABLE 14:** *Summary of clinical report of endemic Trypanosoma vivax*.

Study	Study design	Infection rate	Morbidity	Mortality and death to case ratio	Asymptomatic animals?	Timeframe of case identification and follow‐up	Grade
Rodrigues et al 2015^58^	Cross‐sectional	30/180 (17%) Donkeys	No association between *T. vivax* and PCV or BCS	No follow‐up	Yes (30/30)	No follow‐up	Low

A summary of infection rate, morbidity, mortality, and death to case ratios for each *Trypanosoma* sp. are collated in Table 15; forest plots are presented in Data S3.

**TABLE 15:** Summary of outcome variables for *Trypanosoma* sp. outbreaks.

*Trypanosoma* sp. (proportion (95% CI))	*Trypanosoma evansi*	*Trypanosoma equiperdum*	Tsetse transmitted trypanosomes	*Trypanosoma vivax*
Infection rate	42 (14–76)	12 (7–18)	46 (29–63)	43 (10–83)
Morbidity	47 (13–85)	25 (9–49)	46 (29–63)	43 (10–83)
Mortality	23 (7–54)	No data	6 (1–19)	15 (0–100)
Death to case ratio	45 (20–73)	No data	12 (2–38)	32 (0–100)

*Note*: Data were not suitable for synthesis for endemic disease reports except for endemic tsetse transmitted trypanosomiasis. The infection rate was 22% (95% CI 4–66), morbidity (no data), mortality 8% (0–100) and death to case ratio 19% (1–87).

## 4 DISCUSSION AND CONCLUSIONS

This is the first study to assess the impact of equine trypanosomiasis on a global level. Given the wide geographical prevalence demonstrated (Study question 1) and the severity of the syndromes described (Study question 2) there is *strong evidence in favour* of equine trypanosomiasis being a significant contributor to global mortality and morbidity in equines. This supports the study hypothesis, but with the challenge of having high confidence in specific conclusions due to study quality and gaps in available knowledge.

### 4.1 Study question 1: Prevalence

The clear limitations and wide anticipated error margins in compiling these data are outweighed by the value of permitting the reader to understand, with the most complete evidence available, the challenge of this disease to our world equine population.

By estimate from this study 12% of the approximately 69 million equine population for which data were available are affected by trypanosomiasis. This is likely to be an underestimate. There are vast geographical regions where large numbers of equines are known to reside^39^ which have been additionally risk assessed as medium to high risk in this systematic review. Data sources demonstrate disease in other species^46^, ^48^ and vectors are present but disease reports are absent.^191^ Sixty‐seven and a half million equines are estimated to be at medium or high risk of exposure to trypanosomiasis. Engagement with veterinary professionals and relevant government departments to fill in these important data gaps is a crucial next step. The predominance of disease reports from LMIC countries and the role of equines within these countries in supporting the livelihood of people, mirror the burden of tropical disease in human's termed ‘neglected tropical diseases’ (NTDs) like Human African Trypanosomiasis and Chagas disease (American Trypanosomiasis). This recognition in the early 2000's of NTDs revolutionised the funding landscape and focus on these diseases.^195^ A similar approach could be beneficial for veterinary disease affecting livestock, working equines and poultry to recognise the impact that ‘veterinary neglected tropical diseases’ have within a One Health framework.

Notable limitations of the available data should be considered in reading this review and designing future studies to address knowledge gaps. Prevalence estimates are limited by small sample sizes, infrequent surveying and small areas of geographical representation of screened populations. There are large geographical areas with sparse or no data reported. There is significant bias from selection criteria within studies and publication bias (evident from lack of reporting of negative results). In addition, surveillance data may not be published, countries with no known disease burden are unlikely to test (except for during import testing) and LMIC countries may not have the resources to run surveillance programs.

A large degree of heterogeneity of included studies was noted; primarily clinical (equine selection criteria) and methodological (variable diagnostics, variable local prevalence of disease (impacting upon the positive and negative predictive values for the tests used)).

Objective assignment of disease free status is challenging. There is no disease free status for trypanosomiasis awarded by WOAH. Many countries may be assumed to be disease free due to their higher level of general disease surveillance and prior knowledge of vector distribution (e.g., UK).

Differentiation of *Trypanozoon* is complex and requires results of diagnostic tests to be evaluated in the context of clinical signs and the disease epidemiology.^196^ For the purposes of this review, the diagnosis made by the authors in the reported studies was the one recorded.

### 4.2 Study question 2: Morbidity and mortality

These collated studies provide strong evidence for the importance of *Trypanosoma* spp. as an equine pathogen. The clinical data demonstrate the capability of trypanosomes to cause severe haemolymphatic pathology progressing to neuropathology in neuroinvasive forms which are frequently fatal. Within outbreaks morbidity (25%–47%), mortality rates (6%–23%) and case fatality rates (12%–45%) were high. Available data for endemic trypanosomiasis also documented high mortality (8%) and case fatality rates (19%). The disease can result in abortion and infertility^96^, ^144^, ^184^ which will further impact equine populations.

These data suggest there is diversity in the clinical presentation, speed of progression, and extent of disease spread. The reported clinical phenotypes fall into five overlapping categories, namely, haemolymphatic, wasting, neurological (spinal), neurological (encephalitis) and infertility. The pathophysiology of wasting in equines with trypanosomiasis has not been explored, but based upon work from other species, is thought to derive from anaemia (reducing oxygen delivery to tissues), chronic inflammation (causing anorexia, pyrexia, promoting muscle breakdown and increasing energy expenditure), protein catabolism (parasite consumption of glucose and amino acids, chronic inflammation triggering breakdown of muscle protein), nutrient imbalance (reduced feed intake due to anorexia and parasite consumption of glucose and amino acids), organ dysfunction (including liver and spleen impairing metabolism and detoxification), and immune system exhaustion (from persistent parasitaemia increasing susceptibility to secondary infections).^197^, ^198^ Attempts to clinically differentiate between weakness (secondary to muscle atrophy) and spinal ataxia are missing from most manuscripts.

The temporal progression from haemolymphatic disease to central neurological disease for neuroinvasive species varies. Some equines present with per acute neurological disease^130^; others are delayed up to 20 months.^5^, ^183^, ^194^, ^199^ In the interim period, equines can be asymptomatic^183^; symptoms can wax and wane or display signs of wasting and anaemia.^5^, ^194^ It is not clear if this is an inevitable progression in all cases from haemolymphatic disease in the absence of trypanocidal treatment.

Neuroanatomical localisation based on clinical signs can be consistent with myelitis (hindlimb ataxia)^107^, ^110^, ^126^, ^143^, ^167^ and/or encephalitis (with forebrain and brain stem deficits).^199^, ^200^ There is a differing presentation described for donkeys compared with horses.^101^, ^194^

This diversity in phenotype is observed in all infected species and could be due to *Trypanosoma* spp., strain, virulence, challenge level, timepoint in the disease evolution, health status and immunity of the equine population.^201^

There is *weak evidence in favour* of investigating further whether there is a minimally symptomatic/asymptomatic disease state for equines. Several studies report parasitaemic equines without apparent clinical signs.^58^, ^59^, ^133^ High seropositivity for *Trypanosoma* spp. is reported in some apparently healthy equines.^68^ Both scenarios would benefit from further clinical evaluation with follow‐up (since evolution of disease can be prolonged).

The practical intervention of twice daily monitoring of rectal temperature by owners in high challenge regions may be achievable even in countries with fractured animal health systems if equipment and training are provided. This type of approach may form part of a necessary shift towards more strategic population‐level interventions to maximise use of limited resources.

Clinical data are infrequently described completely in reports involving morbidity and mortality caused by equine trypanosomiasis. Clinical acumen, including neurological evaluations, appears limited in many studies (including differentiation of weakness vs. ataxia). The ability to categorise (acute/chronic disease; haemolymphatic/neurological signs) is limited. Follow‐up time and diagnostics vary. Study designs are predominantly descriptive. Some equines received treatment, but timing, drug, dose, and route of administration were often not described.

### 4.3 Conclusions

These combined objective assessments of existing literature on equine trypanosomiasis highlight important knowledge and capacity gaps. The risk assessment of countries for which there are no data demonstrated the number of countries likely to be at high risk (e.g., due to documented presence of tsetse vector and large equine populations). These countries are a priority to acquire baseline prevalence data. It is clear from the quality of published work that research, clinical, and diagnostic capacity is challenging in many of these LMIC countries. To ensure studies generate useful data, establishing sustainable positive collaborations with capable and ethical partners is key for capacity building. In particular, engagement to support optimisation of study design for suitable sample sizes and random selection protocols is recommended. A protocol to standardise the use of diagnostic tests would also facilitate the assessment of temporal trends and comparison of geographical regions.

Within clinical data, similar gaps are evident. Whilst large amounts of money have been invested in basic science research on trypanosomes, the translation into impact in the field has been poor to date. This may partly derive from the lack of value assigned to clinical observations, expertise, and connection with those working in the field by funding agencies and basic science researchers. Information flow between field observations and findings from laboratory models is key for both to benefit optimally. Collaborative discussion between stakeholders including owners, animal health professionals working in the field, specialist veterinary clinical academics, and those involved in contributing to animal health system design will enable co‐identification of priority clinical questions and optimisation of study design given constraints in resources. There is a need to also capacity build clinical observational skills, as these are valuable data that require minimal equipment to acquire. These are fundamental connections and changes to the current status quo that will form the foundations of improving evidence‐based veterinary medicine in LMIC countries.

## FUNDING INFORMATION

Alexandra G. Raftery is funded by the Vet Fund (University of Glasgow).

## CONFLICT OF INTEREST STATEMENT

The authors declare no conflicts of interest.

## AUTHOR CONTRIBUTIONS

**Alexandra G. Raftery:** Conceptualization; investigation; writing – original draft; methodology; visualization; writing – review and editing; formal analysis; project administration; data curation; funding acquisition. **Lauren Gummery:** Writing – review and editing; investigation. **Karelhia Garcia:** Conceptualization; writing – review and editing. **Dinesh Mohite:** Conceptualization; writing – review and editing. **Paul Capewell:** Writing – review and editing; supervision. **David Sutton:** Writing – original draft; writing – review and editing; supervision.

## DATA INTEGRITY STATEMENT

Alexandra G. Raftery had full access to all the data in the study and takes responsibility for the integrity of the data and the accuracy of data analysis.

## Supporting information


**Data S1.** Study protocol.

**Data S2.** Forest plots of prevalence meta‐analyses by country (R script at end of document).

**Data S3.** Forest plots of infection rate, morbidity, mortality and death to case ration by *Trypanosoma* sp. (R script at end of document).

**Figure S1.** PRISMA flow diagram.

**Table S1.** Results of search strategy following removal of duplicates, application of general inclusion and exclusion criteria.

**Table S2.** Quality assessment scoring system for prevalence data.

**Table S3.** Extracted prevalence data and equine populations by country.

**Table S4.** Extracted data for economic status, reported trypanosome status and equine population by country.

**Table S5.** Extracted data for trypanosome control measures by continent.

## Data Availability

The data that support the findings of this study are openly available in Figshare (www.Figshare.com) at http://doi.org/10.6084/m9.figshare.30331726.
