# Supplementary material for: Equine trypanosomiasis, a systematic review and meta‐analyses: Prevalence, morbidity and mortality
Source: Equine Vet J. 2025 Oct 23;58(2):291–319. doi: 10.1111/evj.70101 (PMC12892385; doi:10.1111/evj.70101)
Supplement: Supplementary file 2 — Data S2. Forest plots of prevalence meta‐analyses by country (R script at end of document). [file EVJ-58-291-s008.pdf]

Equine Veterinary Journal BEVA

Data S2: Meta-analyses of *Trypanosoma* sp. prevalence data.

Country level analyses

1. **Algeria**

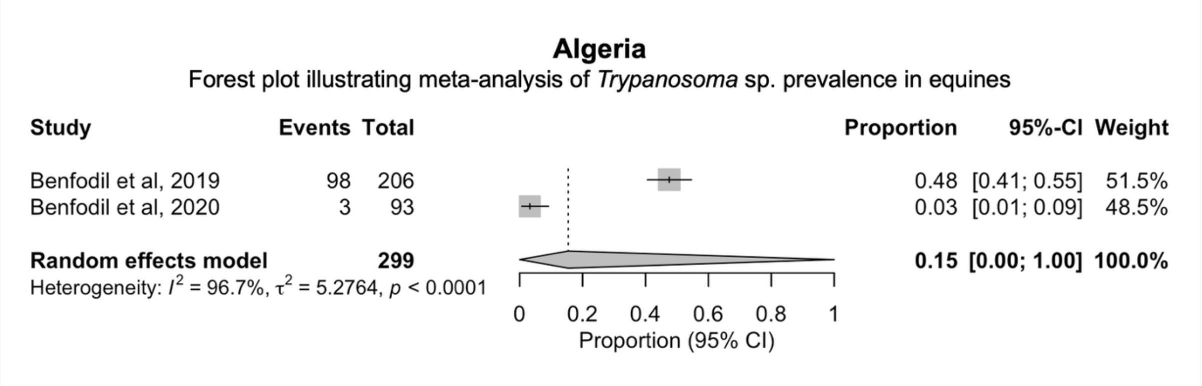

2. **Argentina**

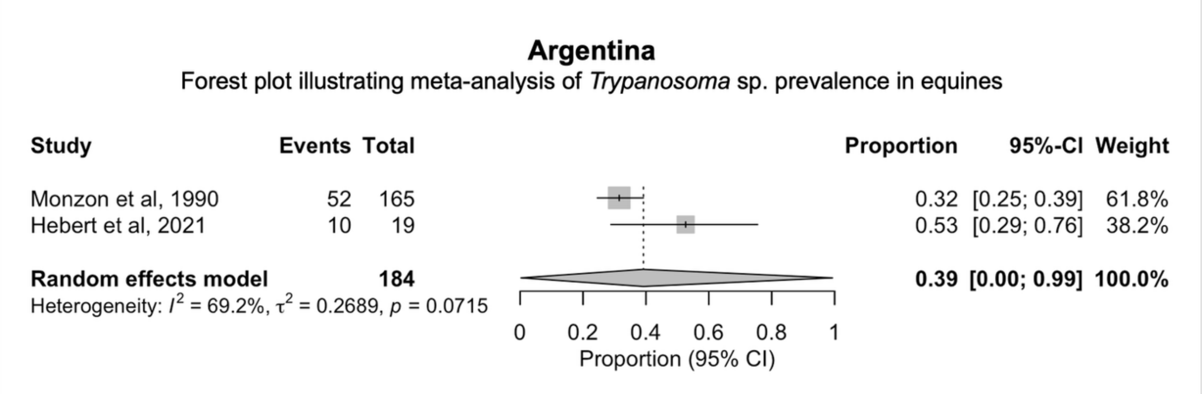

3. **Brazil**

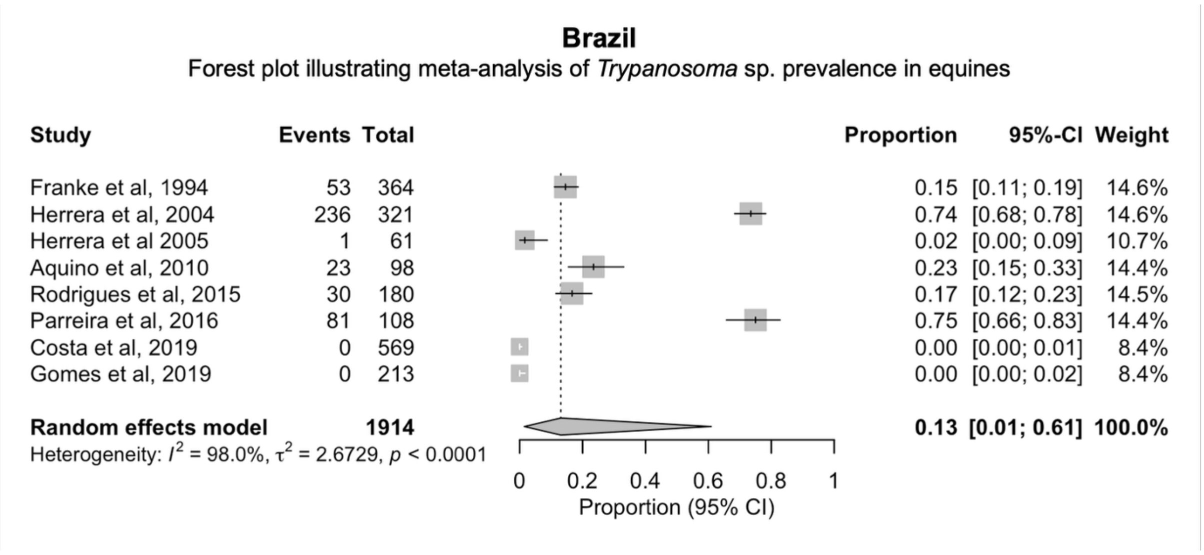

4. **Burkina Faso**

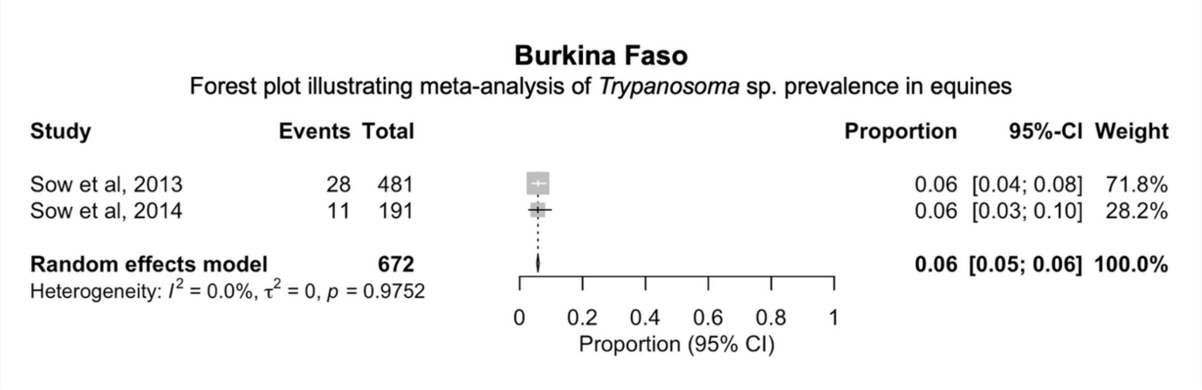

5. **Chad**

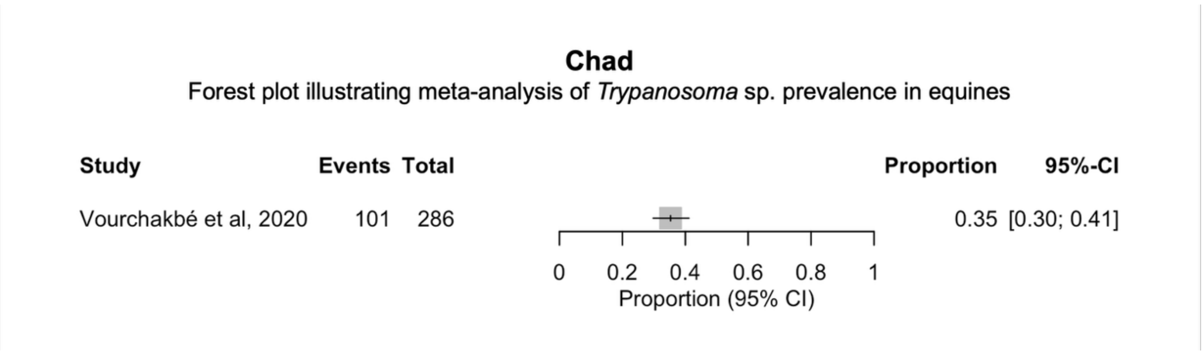

6. **China**

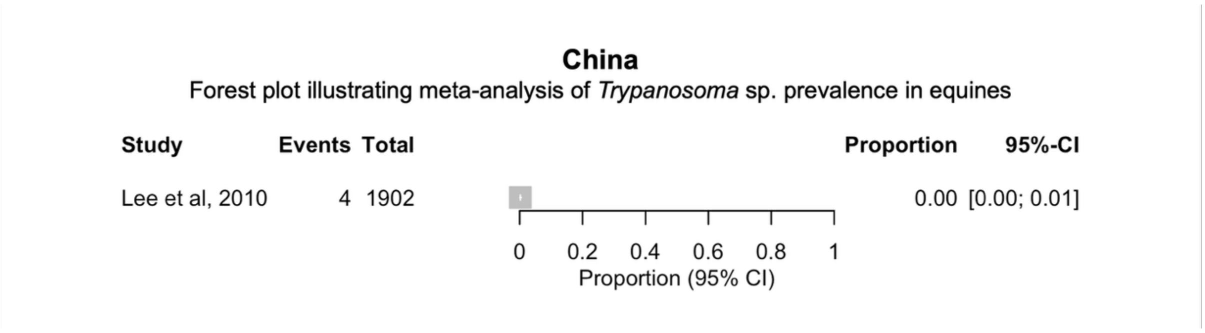

7. **Columbia**

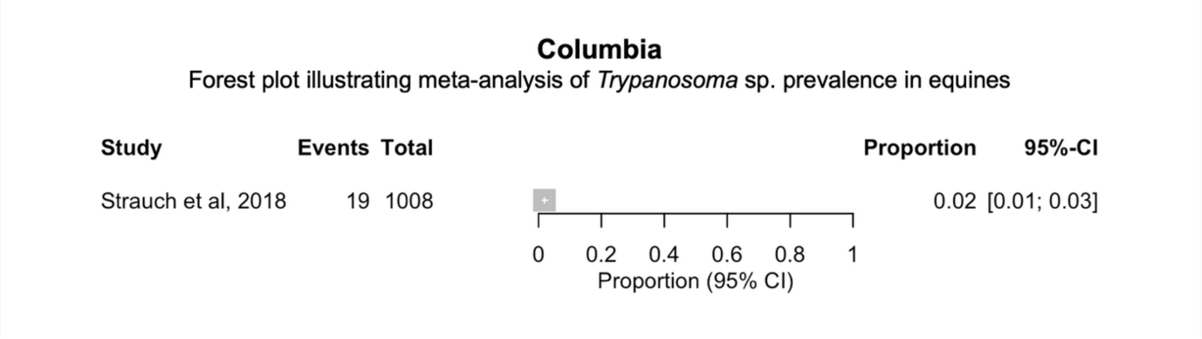

8. Egypt

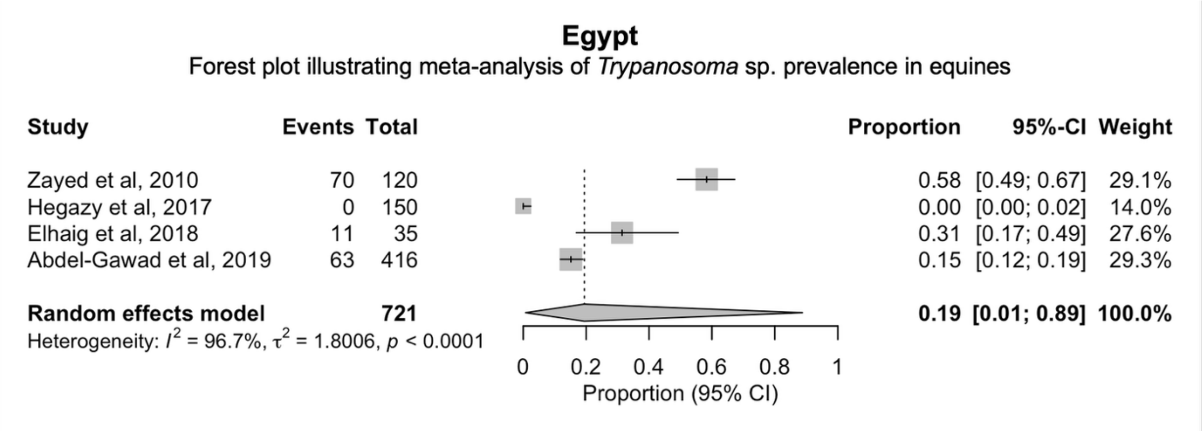

9. Ethiopia

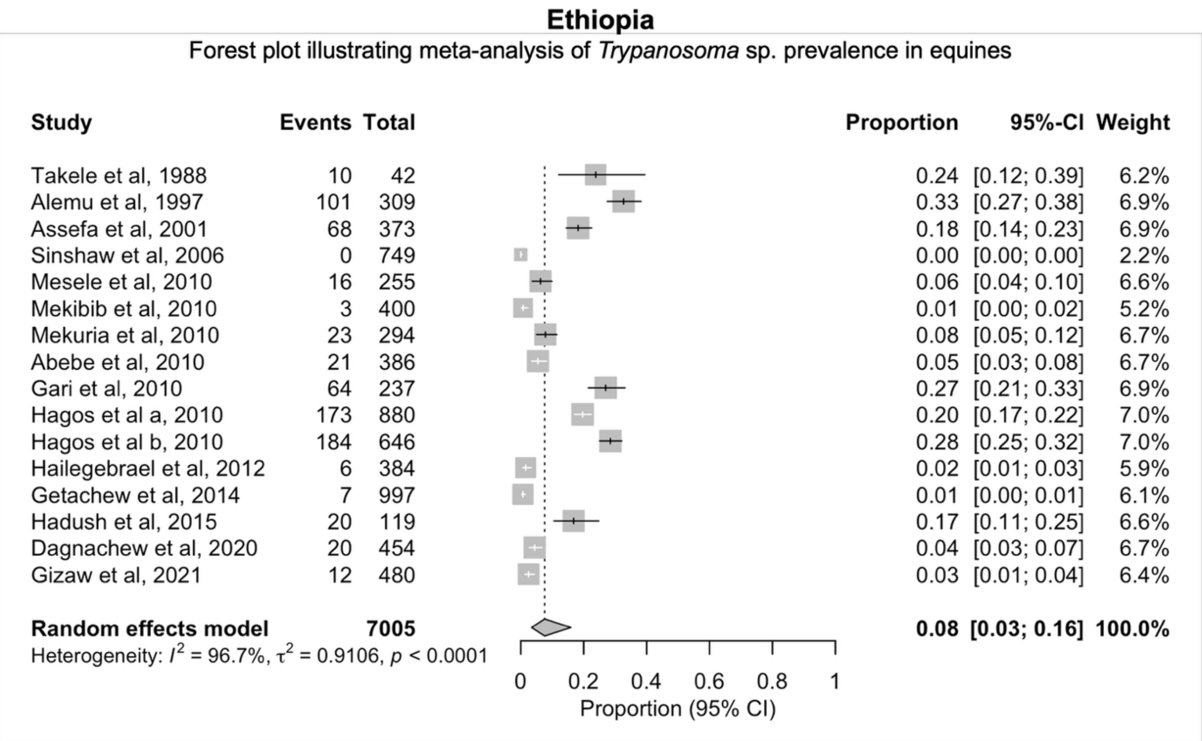

10. Gambia

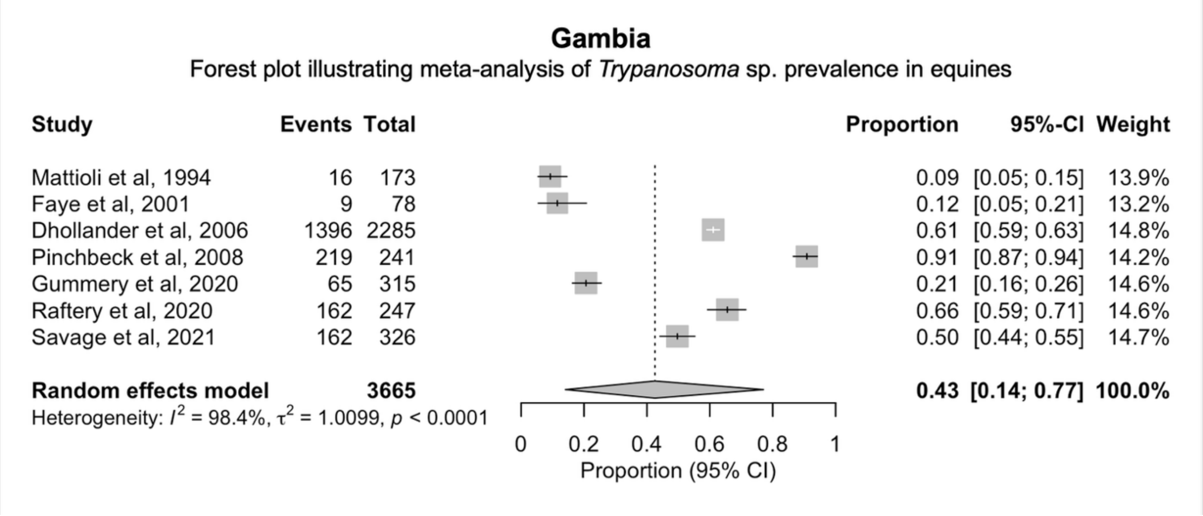

11. Ghana

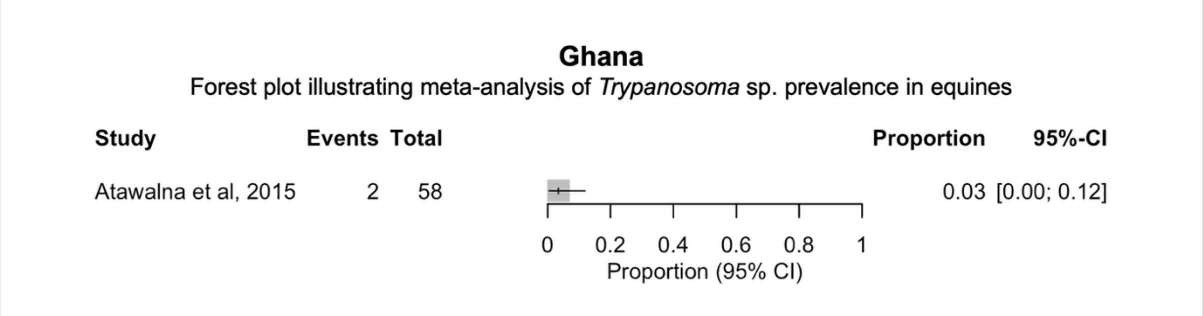

12. Greece

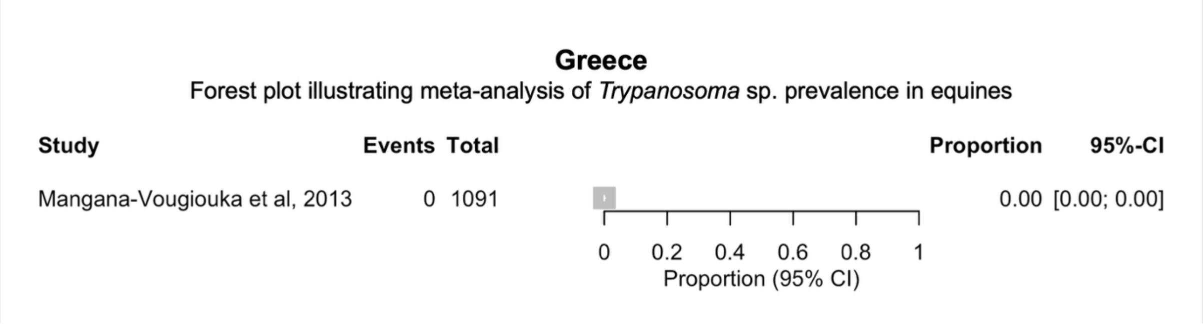

13. India

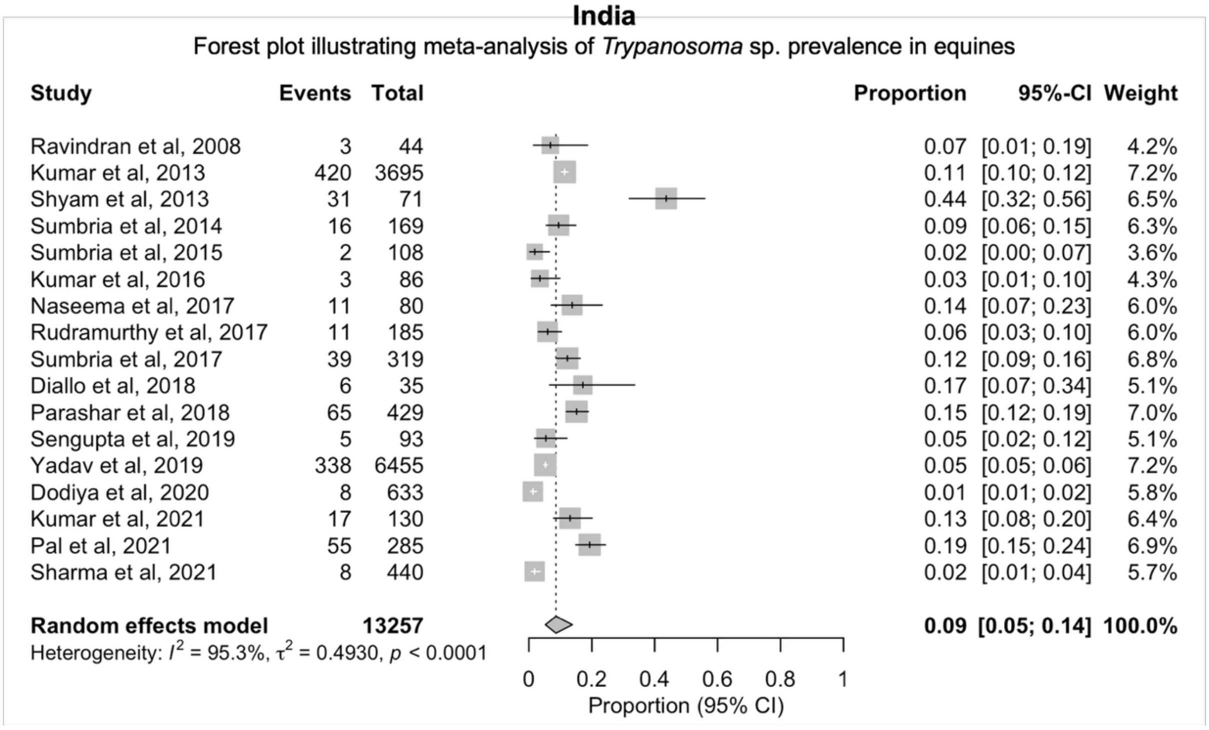

14. Indonesia

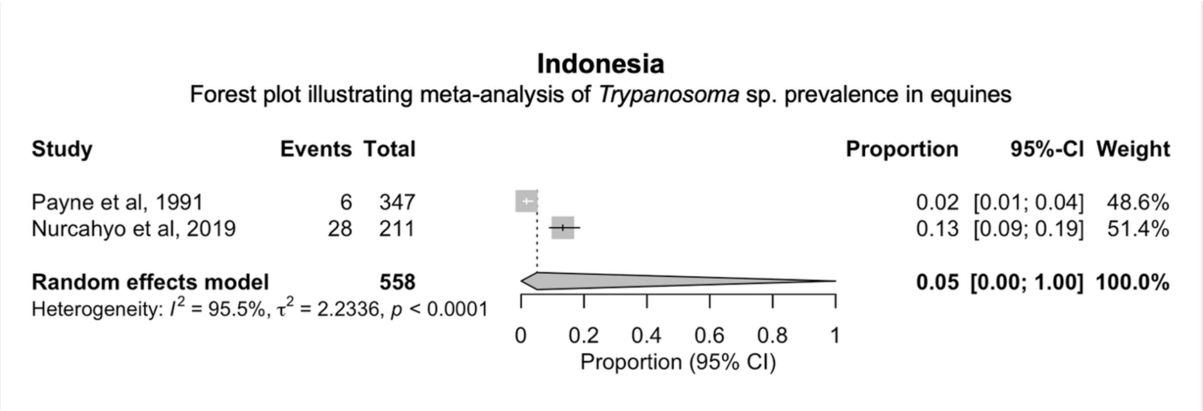

15. Israel

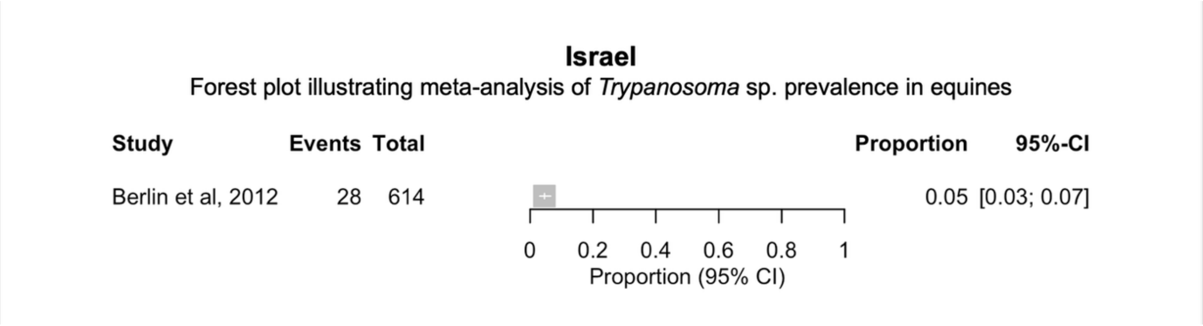

16. Italy

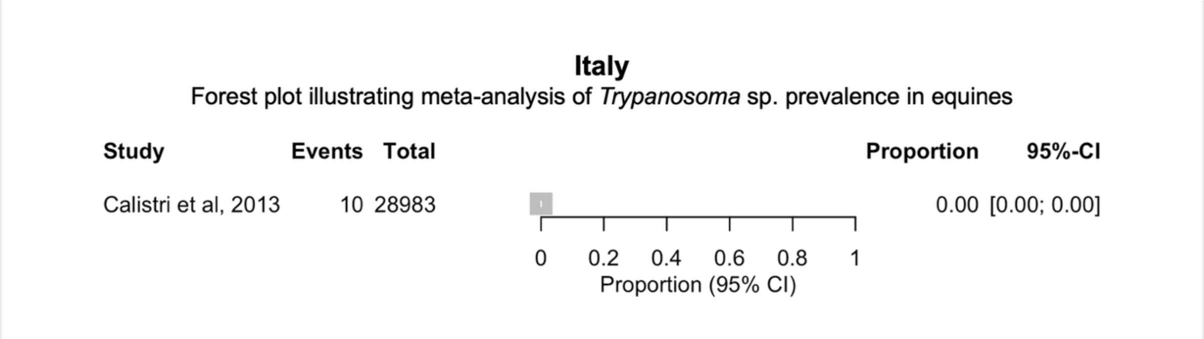

17. Jordan

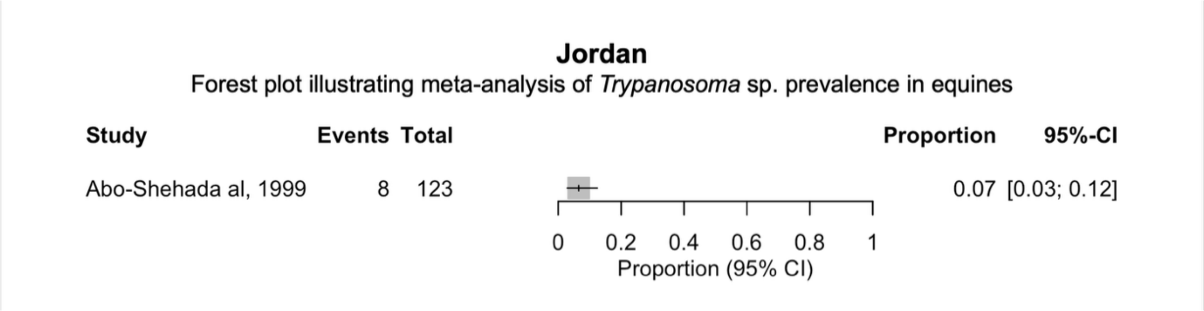

18. Kazakhstan

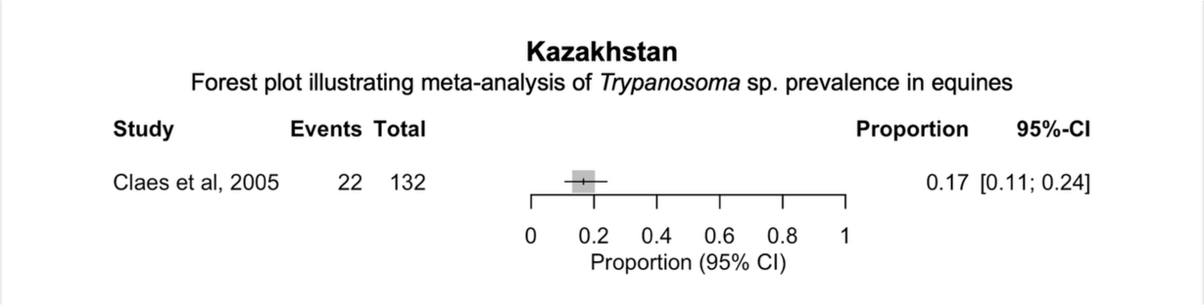

19. Malaysia

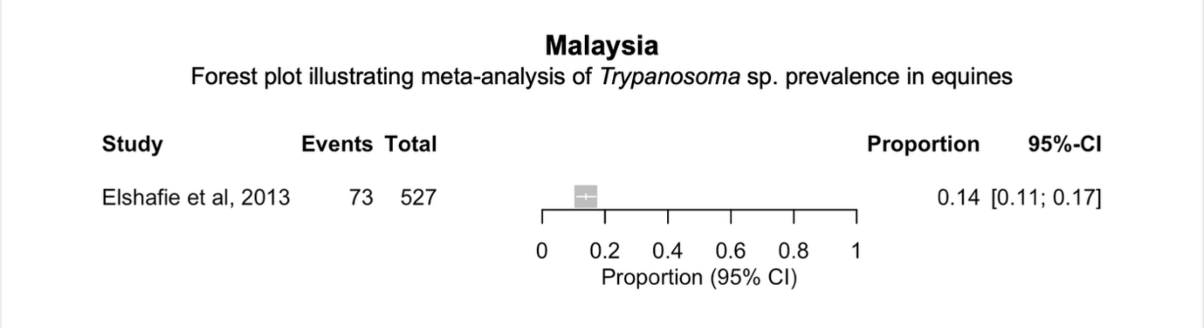

20. Mongolia

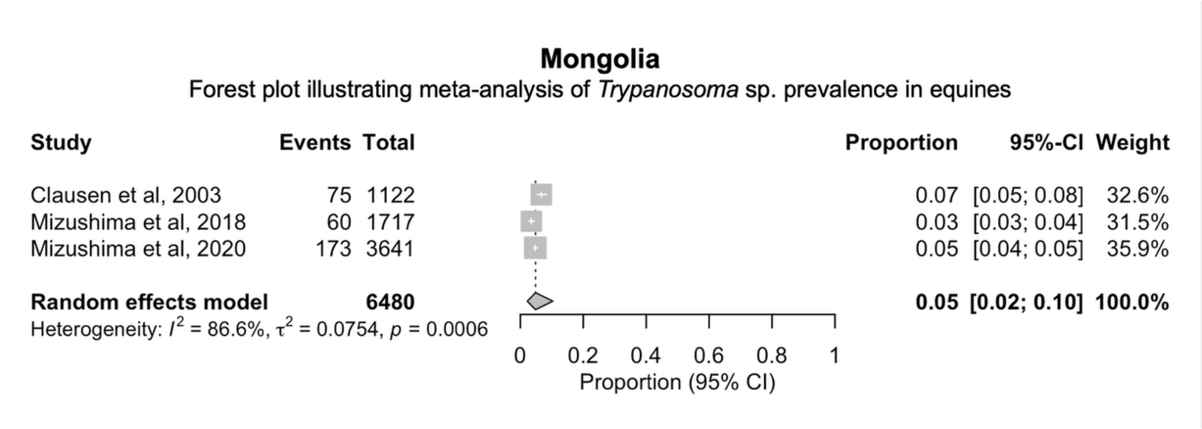

21. Namibia

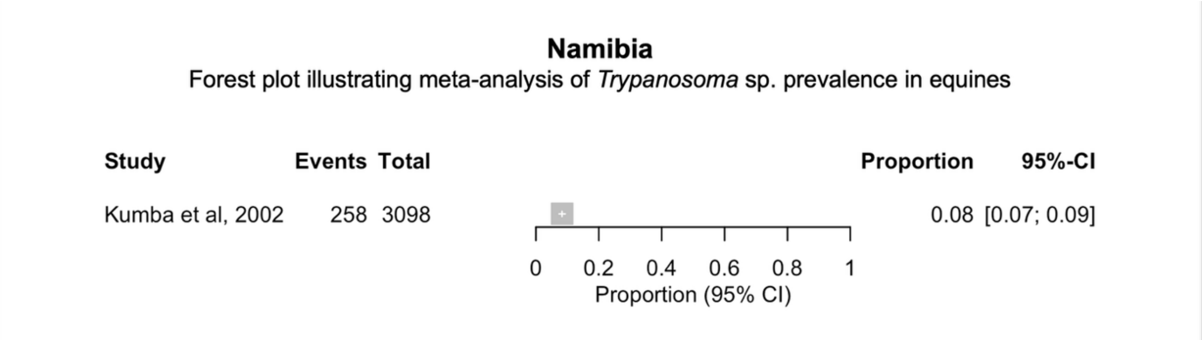

22. Nigeria

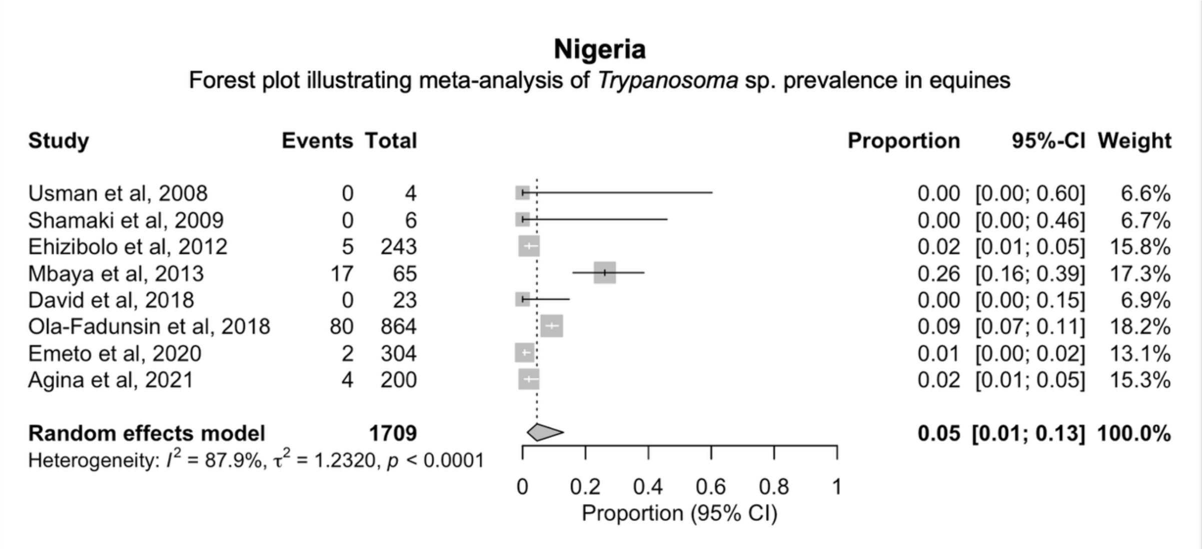

23. Pakistan

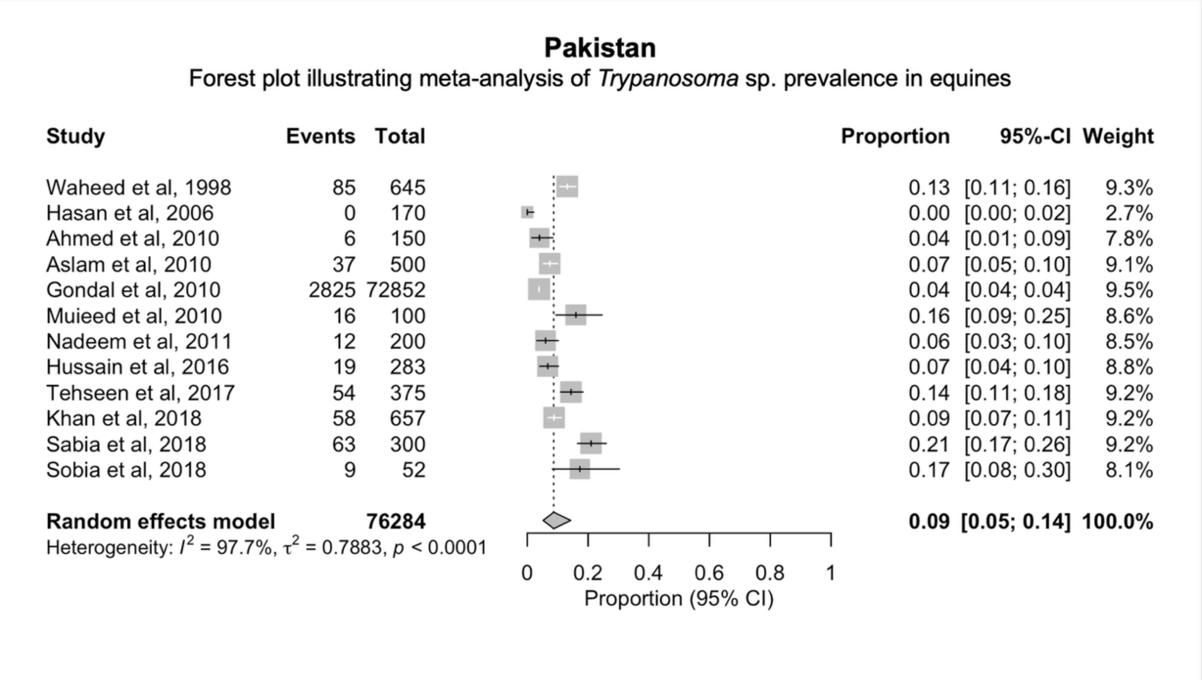

24. Palestine

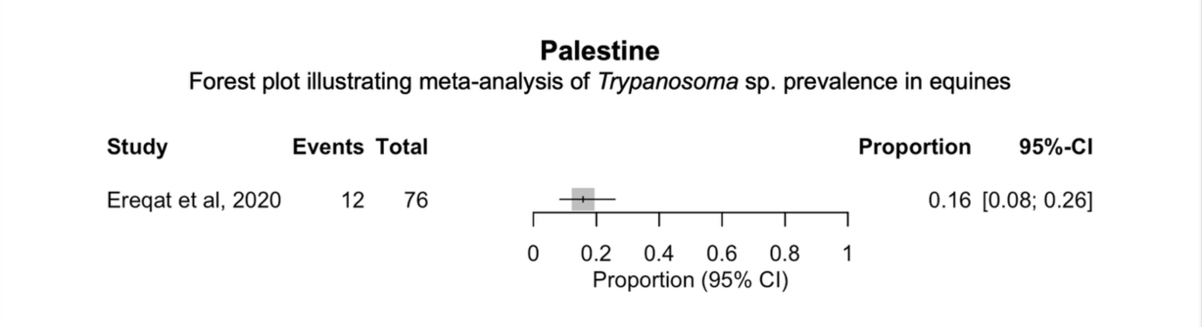

25. Papua New Guinea

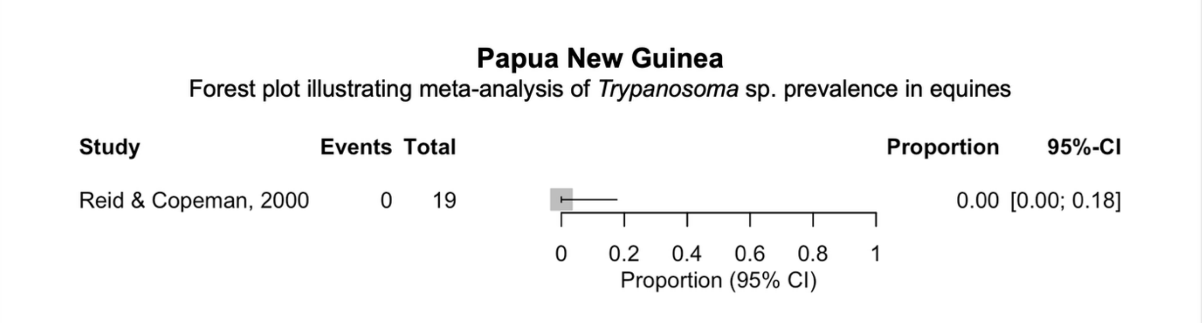

26. Paraguay

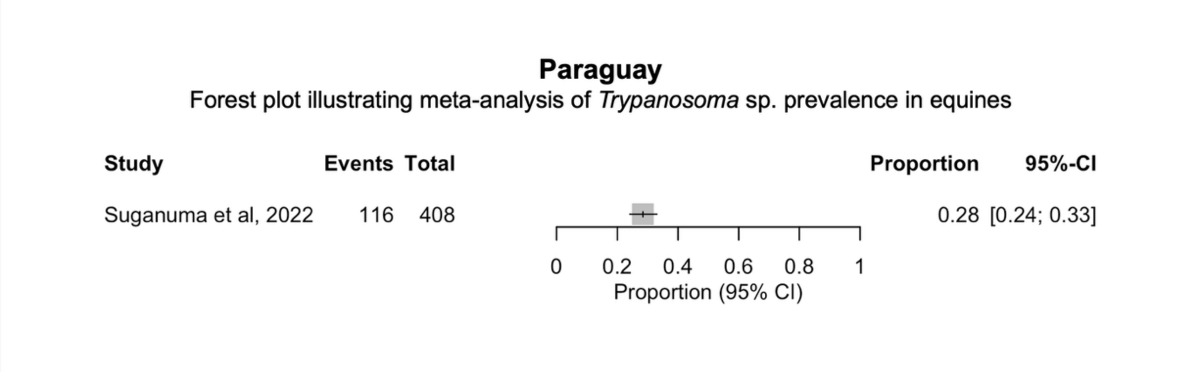

27. Philippines

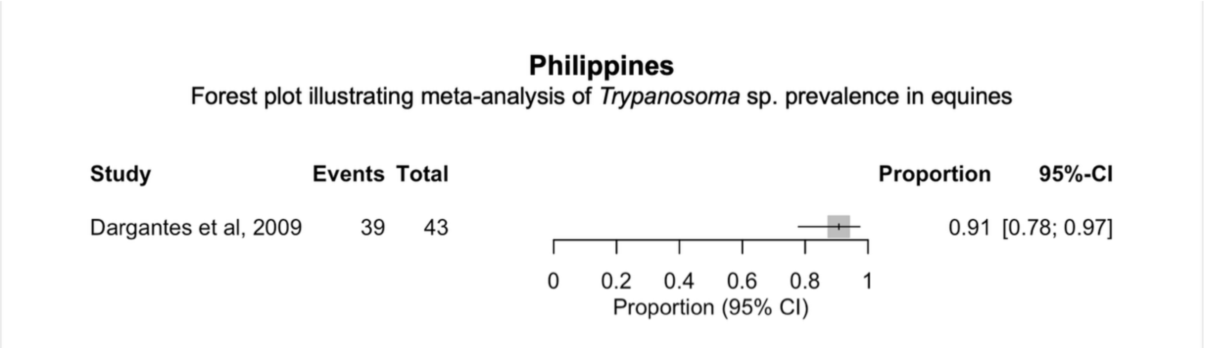

28. Saudi Arabia

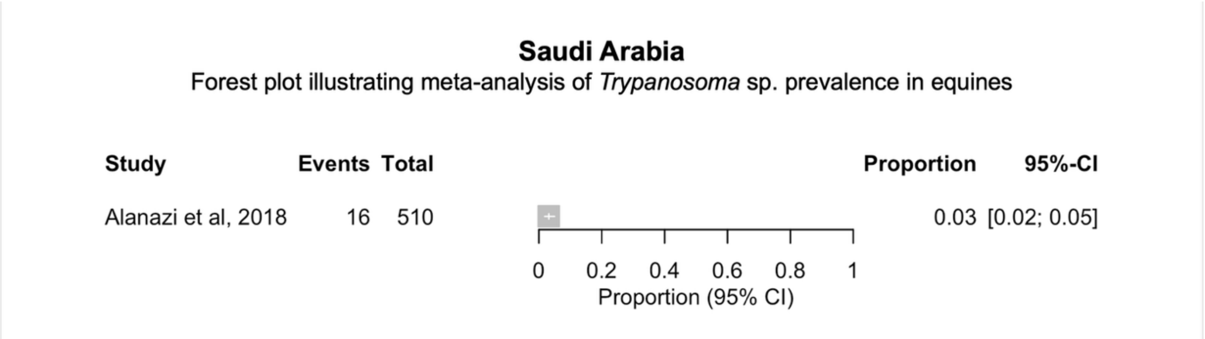

29. Senegal

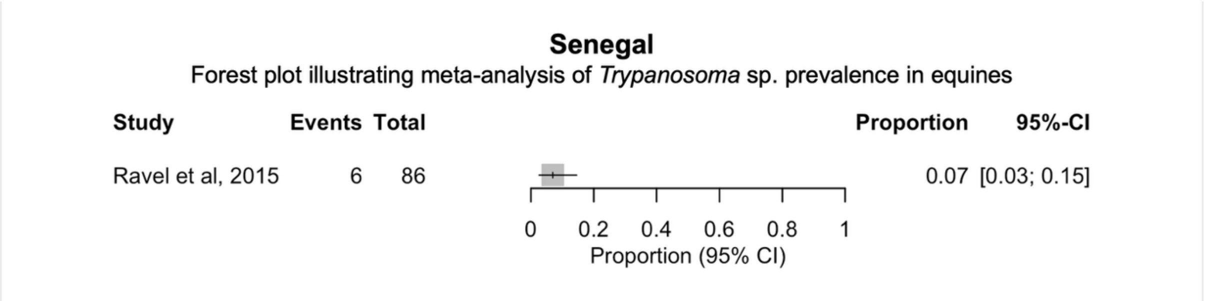

30. Spain

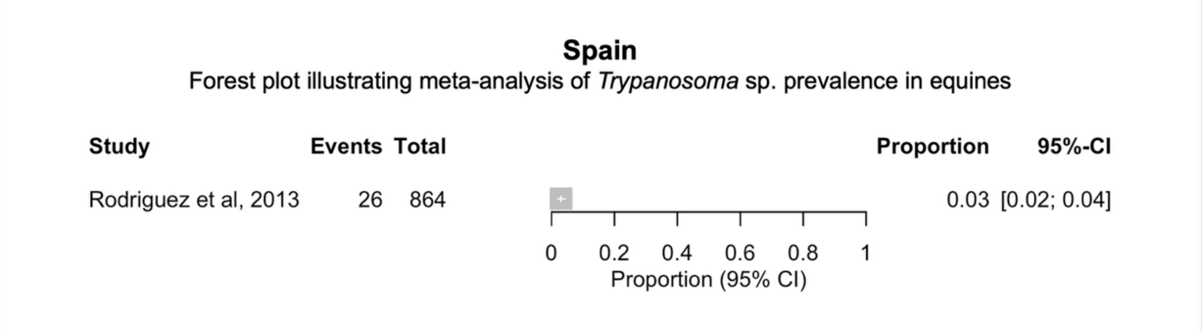

31. Sudan

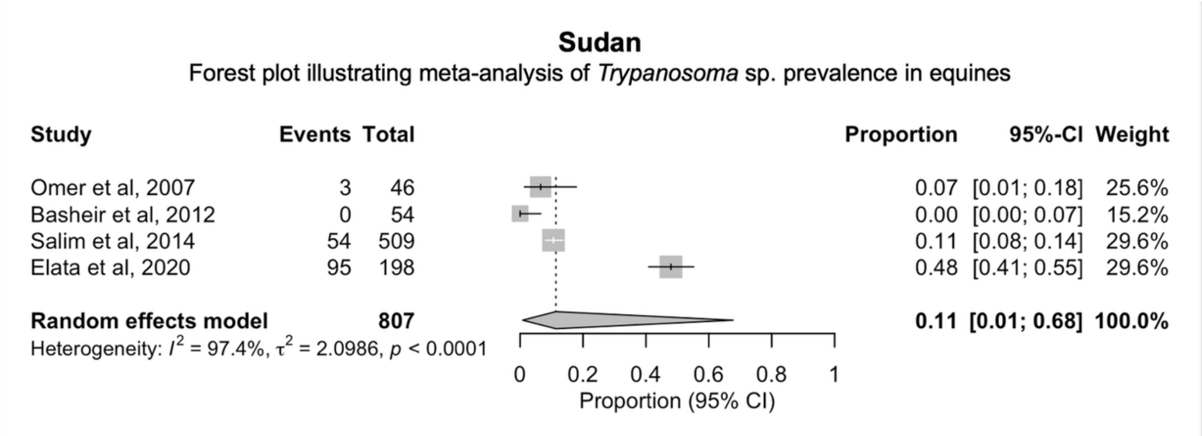

32. Thailand

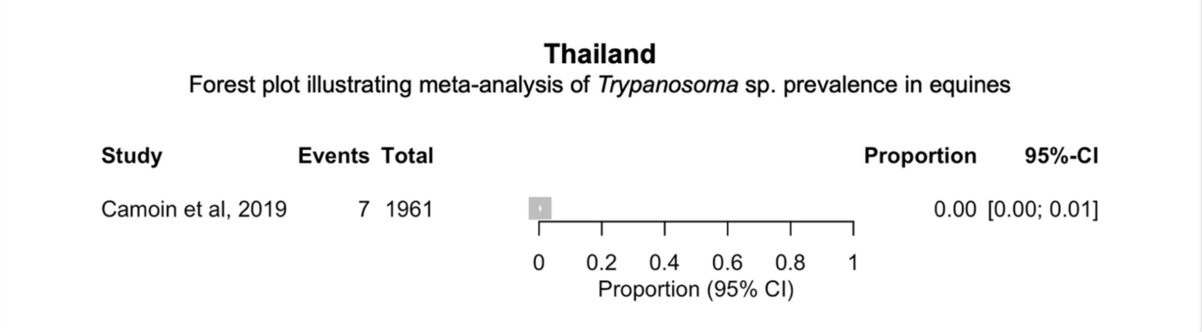

33. Turkey

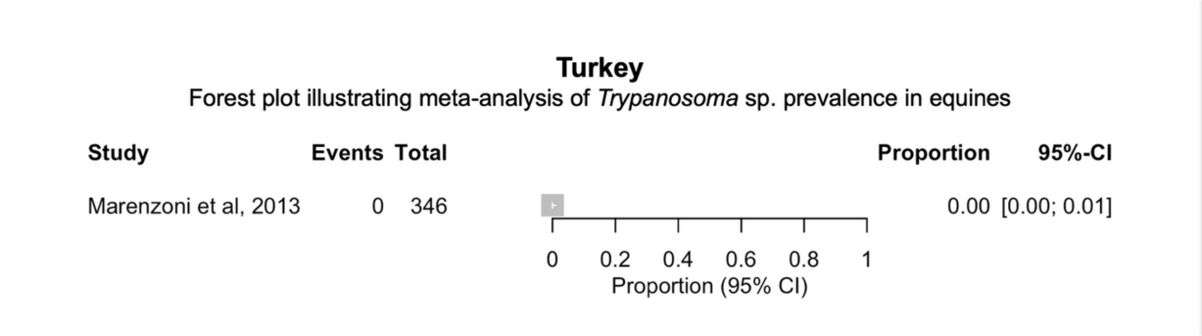

34. Uganda

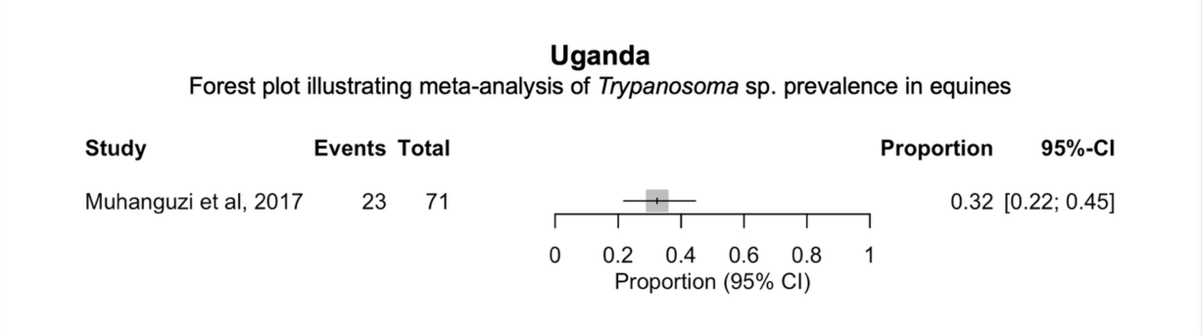

35. United Arab Emirates

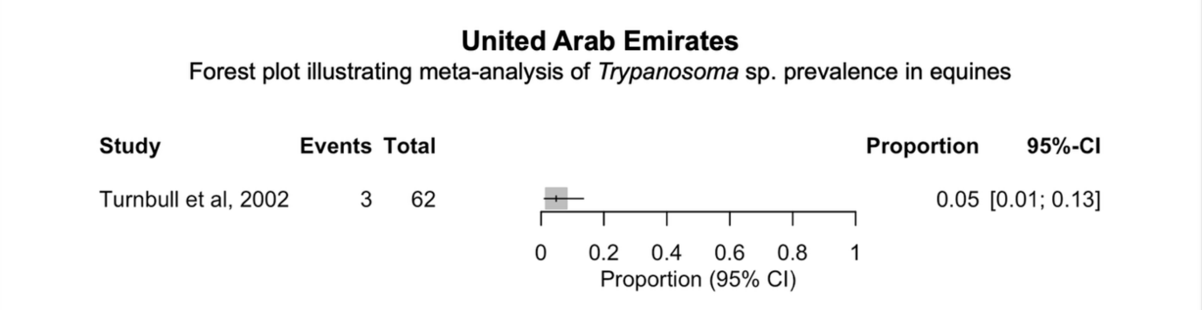

36. Venezuela

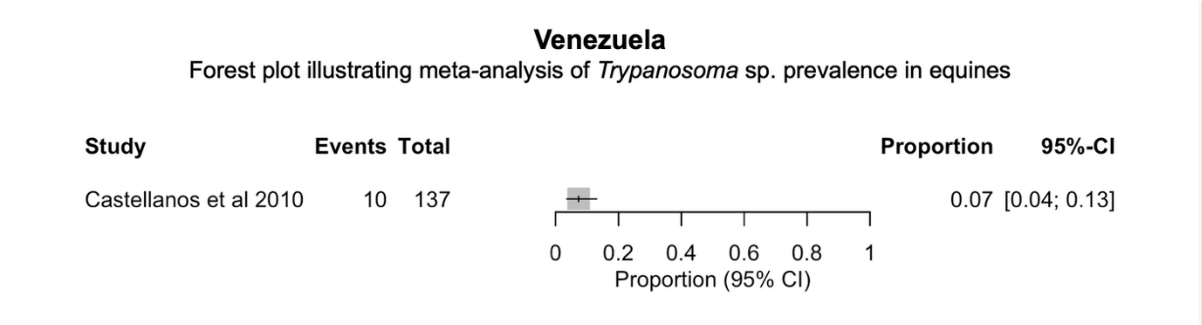

R script for meta-analyses and forest plots

```
#Use random-effects models (account for between study variability).
# Assess heterogeneity (I2 statistic) and study quality

install.packages("meta")
install.packages("metafor")

library(meta)
library(metafor)

#Algeria
Algeria<- data.frame(
  studyid=c("Benfodil et al, 2019","Benfodil et al, 2020" ),
  events=c(98, 3),
  n= c(206, 93)
)
```

```
m1<-metaprop(event=events,
          n=n,
          data=Algeria,
          sm="PLOGIT", #Logit-transformed proportion
          method="Inverse", #inverse -variance weighting used PFT transformation
          method.tau="DL", #DerSimonian-Laird estimator
          method.random.ci = "HK", #Hartung-Knapp adjustment for CI
          random=TRUE,
          common=F,
          studlab= studyid
)

summary(m1)

forest(m1,
      title="Algeria: Meta-analysis of Prevalence",
      xlab="Proportion (95% CI)",
      xlim = c(0, 1),
      colgap.forest.left="2cm"

      )

#Argentina
Argentina<- data.frame(
  studyid=c("Monzon et al, 1990","Hebert et al, 2021" ),
  events=c(52, 10),
  n= c(165, 19)
)

m2<-metaprop(event=events,
          n=n,
          data=Argentina,
          sm="PLOGIT", #Logit-transformed proportion
          method="Inverse", #inverse -variance weighting used PFT transformation
          method.tau="DL", #DerSimonian-Laird estimator
          method.random.ci = "HK", #Hartung-Knapp adjustment for CI
          random=TRUE,
          common=F,
          studlab= studyid
)

summary(m2)

forest(m2,
      title="Argentina: Meta-analysis of Prevalence",
      xlab="Proportion (95% CI)",
      xlim = c(0, 1),
      colgap.forest.left="2cm"

)

#Brazil

Brazil<- data.frame(
  studyid=c("Franke et al, 1994","Herrera et al, 2004","Herrera et al 2005",
          "Aquino et al, 2010" , "Rodrigues et al, 2015",
          "Parreira et al, 2016", "Costa et al, 2019", "Gomes et al, 2019"),
  events=c(53, 236, 1, 23, 30, 81, 0, 0),
  n= c(364, 321, 61, 98, 180, 108, 569, 213)
)
```

```
m3<-metaprop(event=events,
          n=n,
          data=Brazil,
          sm="PLOGIT", #Logit-transformed proportion
          method="Inverse", #inverse -variance weighting used PFT transformation
          method.tau="DL", #DerSimonian-Laird estimator
          method.random.ci="HK", #Hartung-Knapp adjustment for CI
          random=TRUE,
          common=F,
          studlab= studyid
)

summary(m3)

forest(m3,
      title="Brazil: Meta-analysis of Prevalence",
      xlab="Proportion (95% CI)",
      xlim = c(0, 1),
      colgap.forest.left="2cm"

)

#Burkino Faso

BurkinaFaso<- data.frame(
  studyid=c("Sow et al, 2013","Sow et al, 2014" ),
  events=c(28, 11),
  n= c(481, 191)
)

m4a<-metaprop(event=events,
          n=n,
          data=BurkinaFaso,
          sm="PLOGIT", #Logit-transformed proportion
          method="Inverse", #inverse -variance weighting used PFT transformation
          method.tau="DL", #DerSimonian-Laird estimator
          method.random.ci="HK", #Hartung-Knapp adjustment for CI
          random=TRUE,
          common=F,
          studlab= studyid
)

summary(m4a)

forest(m4a,
      title="Burkina Faso: Meta-analysis of Prevalence",
      xlab="Proportion (95% CI)",
      xlim = c(0, 1),
      colgap.forest.left="2cm"

)

#Chad
Chad<- data.frame(
  studyid=c("Vourchakbé et al, 2020"),
  events=c(101),
  n= c(286)
)
```

```
m4<-metaprop(event=events,
            n=n,
            data=Chad,
            sm="PLOGIT", #Logit-transformed proportion
            method="Inverse", #inverse -variance weighting used PFT transformation
            method.tau="DL", #DerSimonian-Laird estimator
            method.random.ci="HK", #Hartung-Knapp adjustment for CI
            random=TRUE,
            common=F,
            studlab= studyid
)

summary(m4)

forest(m4,
      title="Chad: Meta-analysis of Prevalence",
      xlab="Proportion (95% CI)",
      xlim = c(0, 1),
      colgap.forest.left="2cm"
)


#China

China<- data.frame(
  studyid=c("Lee et al, 2010"),
  events=c(4),
  n= c(1902)
)

m5<-metaprop(event=events,
            n=n,
            data=China,
            sm="PLOGIT", #Logit-transformed proportion
            method="Inverse", #inverse -variance weighting used PFT transformation
            method.tau="DL", #DerSimonian-Laird estimator
            method.random.ci="HK", #Hartung-Knapp adjustment for CI
            random=TRUE,
            common=F,
            studlab= studyid
)

summary(m5)

forest(m5,
      title="China: Meta-analysis of Prevalence",
      xlab="Proportion (95% CI)",
      xlim = c(0, 1),
      colgap.forest.left="2cm"
)

#Columbia

Columbia<- data.frame(
  studyid=c("Strauch et al, 2018" ),
  events=c(19),
  n= c(1008)
)

m6<-metaprop(event=events,
```

```
            n=n,
            data=Columbia,
            sm="PLOGIT", #Logit-transformed proportion
            method="Inverse", #inverse -variance weighting used PFT transformation
            method.tau="DL", #DerSimonian-Laird estimator
            method.random.ci="HK", #Hartung-Knapp adjustment for CI
            random=TRUE,
            common=F,
            studlab= studyid
)

summary(m6)

forest(m6,
       title="Columbia: Meta-analysis of Prevalence",
       xlab="Proportion (95% CI)",
       xlim = c(0, 1),
       colgap.forest.left="2cm"

)

#Egypt

Egypt<- data.frame(
  studyid=c("Zayed et al, 2010","Hegazy et al, 2017",
           "Elhaig et al, 2018", "Abdel-Gawad et al, 2019" ),
  events=c(70, 0, 11, 63),
  n= c(120, 150, 35, 416)
)

m7<-metaprop(event=events,
            n=n,
            data=Egypt,
            sm="PLOGIT", #Logit-transformed proportion
            method="Inverse", #inverse -variance weighting used PFT transformation
            method.tau="DL", #DerSimonian-Laird estimator
            method.random.ci="HK", #Hartung-Knapp adjustment for CI
            random=TRUE,
            common=F,
            studlab= studyid
)

summary(m7)

forest(m7,
       title="Egypt: Meta-analysis of Prevalence",
       xlab="Proportion (95% CI)",
       xlim = c(0, 1),
       colgap.forest.left="2cm"

)

#Ethiopia

Ethiopia<- data.frame(
  studyid=c("Takele et al, 1988","Alemu et al, 1997", "Assefa et al, 2001",
           "Sinshaw et al, 2006","Mesele et al, 2010", "Mekibib et al, 2010",
           "Mekuria et al, 2010", "Abebe et al, 2010", "Gari et al, 2010",
           "Hagos et al a, 2010", "Hagos et al b, 2010",
           "Hailegebrael et al, 2012", "Getachew et al, 2014",
```

```
          "Hadush et al, 2015", "Dagnachew et al, 2020",
          "Gizaw et al, 2021"),
  events=c(10, 101, 68, 0, 16, 3, 23, 21, 64, 173, 184, 6,7, 20, 20, 12),
  n= c(42, 309, 373, 749, 255, 400, 294, 386, 237, 880, 646, 384, 997, 119, 454, 480)
)

m8<-metaprop(event=events,
          n=n,
          data=Ethiopia,
          sm="PLOGIT", #Logit-transformed proportion
          method="Inverse", #inverse -variance weighting used PFT transformation
          method.tau="DL", #DerSimonian-Laird estimator
          method.random.ci="HK", #Hartung-Knapp adjustment for CI
          random=TRUE,
          common=F,
          studlab= studyid
)

summary(m8)

forest(m8,
      title="Ethiopia: Meta-analysis of Prevalence",
      xlab="Proportion (95% CI)",
      xlim = c(0, 1),
      colgap.forest.left="2cm"

)

#Gambia

Gambia<- data.frame(
  studyid=c("Mattioli et al, 1994","Faye et al, 2001","Dhollander et al, 2006",
          "Pinchbeck et al, 2008", "Gummery et al, 2020", "Raftery et al, 2020",
          "Savage et al, 2021"),
  events=c(16, 9, 1396, 219, 65, 162, 162),
  n= c(173, 78, 2285, 241, 315, 247, 326)
)

m9<-metaprop(event=events,
          n=n,
          data=Gambia,
          sm="PLOGIT", #Logit-transformed proportion
          method="Inverse", #inverse -variance weighting used PFT transformation
          method.tau="DL", #DerSimonian-Laird estimator
          method.random.ci="HK", #Hartung-Knapp adjustment for CI
          random=TRUE,
          common=F,
          studlab= studyid
)

summary(m9)

forest(m9,
      title="Gambia: Meta-analysis of Prevalence",
      xlab="Proportion (95% CI)",
      xlim = c(0, 1),
      colgap.forest.left="2cm"

)
```

```
#Ghana

Ghana<- data.frame(
  studyid=c("Atawalna et al, 2015" ),
  events=c(2),
  n= c(58)
)

m10<-metaprop(event=events,
          n=n,
          data=Ghana,
          sm="PLOGIT", #Logit-transformed proportion
          method="Inverse", #inverse -variance weighting used PFT transformation
          method.tau="DL", #DerSimonian-Laird estimator
          method.random.ci = "HK", #Hartung-Knapp adjustment for CI
          random=TRUE,
          common=F,
          studlab= studyid
)

summary(m10)

forest(m10,
      title="Ghana: Meta-analysis of Prevalence",
      xlab="Proportion (95% CI)",
      xlim = c(0, 1),
      colgap.forest.left="2cm"

)

#Greece

Greece<- data.frame(
  studyid=c("Mangana-Vougiouka et al, 2013" ),
  events=c(0),
  n= c(1091)
)

m11<-metaprop(event=events,
          n=n,
          data=Greece,
          sm="PLOGIT", #Logit-transformed proportion
          method="Inverse", #inverse -variance weighting used PFT transformation
          method.tau="DL", #DerSimonian-Laird estimator
          method.random.ci = "HK", #Hartung-Knapp adjustment for CI
          random=TRUE,
          common=F,
          studlab= studyid
)

summary(m11)

forest(m11,
      title="Greece: Meta-analysis of Prevalence",
      xlab="Proportion (95% CI)",
      xlim = c(0, 1),
      colgap.forest.left="2cm"

)
```

```
#India


India<- data.frame(
  studyid=c("Ravindran et al, 2008",
          "Kumar et al, 2013", "Shyam et al, 2013", "Sumbria et al, 2014",
          "Sumbria et al, 2015", "Kumar et al, 2016","Naseema et al, 2017",
          "Rudramurthy et al, 2017", "Sumbria et al, 2017","Diallo et al, 2018",
          "Parashar et al, 2018", "Sengupta et al, 2019", "Yadav et al, 2019",
          "Dodiya et al, 2020", "Kumar et al, 2021", "Pal et al, 2021",
          "Sharma et al, 2021"),
  events=c(3,420, 31, 16, 2, 3, 11, 11, 39, 6, 65, 5, 338, 8, 17, 55, 8),
  n= c(44, 3695, 71, 169, 108, 86, 80, 185, 319, 35, 429, 93, 6455, 633, 130, 285, 440)
)

m12<-metaprop(event=events,
            n=n,
            data=India,
            sm="PLOGIT", #Logit-transformed proportion
            method="Inverse", #inverse -variance weighting used PFT transformation
            method.tau="DL", #DerSimonian-Laird estimator
            method.random.ci = "HK", #Hartung-Knapp adjustment for CI
            random=TRUE,
            common=F,
            studlab= studyid
)

summary(m12)

forest(m12,
      title="India: Meta-analysis of Prevalence",
      xlab="Proportion (95% CI)",
      xlim = c(0, 1),
      colgap.forest.left="2cm"

)



#Indonesia


Indonesia<- data.frame(
  studyid=c("Payne et al, 1991", "Nurcahyo et al, 2019" ),
  events=c(6, 28),
  n= c(347, 211)
)

m13<-metaprop(event=events,
            n=n,
            data=Indonesia,
            sm="PLOGIT", #Logit-transformed proportion
            method="Inverse", #inverse -variance weighting used PFT transformation
            method.tau="DL", #DerSimonian-Laird estimator
            method.random.ci = "HK", #Hartung-Knapp adjustment for CI
            random=TRUE,
            common=F,
            studlab= studyid
)
```

```
summary(m13)

forest(m13,
       title="Indonesia: Meta-analysis of Prevalence",
       xlab="Proportion (95% CI)",
       xlim = c(0, 1),
       colgap.forest.left="2cm"

)

#Israel


Israel<- data.frame(
  studyid=c("Berlin et al, 2012" ),
  events=c(28),
  n= c(614)
)

m14<-metaprop(event=events,
             n=n,
             data=Israel,
             sm="PLOGIT", #Logit-transformed proportion
             method="Inverse", #inverse -variance weighting used PFT transformation
             method.tau="DL", #DerSimonian-Laird estimator
             method.random.ci = "HK", #Hartung-Knapp adjustment for CI
             random=TRUE,
             common=F,
             studlab= studyid
)

summary(m14)

forest(m14,
       title="Israel: Meta-analysis of Prevalence",
       xlab="Proportion (95% CI)",
       xlim = c(0, 1),
       colgap.forest.left="2cm"

)

#Italy


Italy<- data.frame(
  studyid=c("Calistri et al, 2013" ),
  events=c(10),
  n= c(28983)
)

m15<-metaprop(event=events,
             n=n,
             data=Italy,
             sm="PLOGIT", #Logit-transformed proportion
             method="Inverse", #inverse -variance weighting used PFT transformation
             method.tau="DL", #DerSimonian-Laird estimator
             method.random.ci = "HK", #Hartung-Knapp adjustment for CI
             random=TRUE,
             common=F,
             studlab= studyid
```

```
)

summary(m15)

forest(m15,
       title="Italy: Meta-analysis of Prevalence",
       xlab="Proportion (95% CI)",
       xlim = c(0, 1),
       colgap.forest.left="2cm"

)

#Jordan


Jordan<- data.frame(
  studyid=c("Abo-Shehada al, 1999" ),
  events=c(8),
  n= c(123)
)

m16<-metaprop(event=events,
              n=n,
              data=Jordan,
              sm="PLOGIT", #Logit-transformed proportion
              method="Inverse", #inverse -variance weighting used PFT transformation
              method.tau="DL", #DerSimonian-Laird estimator
              method.random.ci = "HK", #Hartung-Knapp adjustment for CI
              random=TRUE,
              common=F,
              studlab= studyid
)

summary(m16)

forest(m16,
       title="Jordan: Meta-analysis of Prevalence",
       xlab="Proportion (95% CI)",
       xlim = c(0, 1),
       colgap.forest.left="2cm"

)

#Kazakhstan


Kazakhstan<- data.frame(
  studyid=c("Claes et al, 2005" ),
  events=c(22),
  n= c(132)
)

m17<-metaprop(event=events,
              n=n,
              data=Kazakhstan,
              sm="PLOGIT", #Logit-transformed proportion
              method="Inverse", #inverse -variance weighting used PFT transformation
              method.tau="DL", #DerSimonian-Laird estimator
              method.random.ci = "HK", #Hartung-Knapp adjustment for CI
              random=TRUE,
```

```
            common=F,
            studlab= studyid
)

summary(m17)

forest(m17,
      title="Kazakhstan: Meta-analysis of Prevalence",
      xlab="Proportion (95% CI)",
      xlim = c(0, 1),
      colgap.forest.left="2cm"

)



#Malaysia


Malaysia<- data.frame(
  studyid=c("Elshafie et al, 2013" ),
  events=c(73),
  n= c(527)
)

m18<-metaprop(event=events,
            n=n,
            data=Malaysia,
            sm="PLOGIT", #Logit-transformed proportion
            method="Inverse", #inverse -variance weighting used PFT transformation
            method.tau="DL", #DerSimonian-Laird estimator
            method.random.ci = "HK", #Hartung-Knapp adjustment for CI
            random=TRUE,
            common=F,
            studlab= studyid
)

summary(m18)

forest(m18,
      title="Malaysia: Meta-analysis of Prevalence",
      xlab="Proportion (95% CI)",
      xlim = c(0, 1),
      colgap.forest.left="2cm"

)

#Mongolia


Mongolia<- data.frame(
  studyid=c("Clausen et al, 2003", "Mizushima et al, 2018", "Mizushima et al, 2020" ),
  events=c(75, 60, 173),
  n= c(1122, 1717, 3641)
)

m19<-metaprop(event=events,
            n=n,
            data=Mongolia,
            sm="PLOGIT", #Logit-transformed proportion
```

```
            method="Inverse", #inverse -variance weighting used PFT transformation
            method.tau="DL", #DerSimonian-Laird estimator
            method.random.ci = "HK", #Hartung-Knapp adjustment for CI
            random=TRUE,
            common=F,
            studlab= studyid
)

summary(m19)

forest(m19,
      title="Mongolia: Meta-analysis of Prevalence",
      xlab="Proportion (95% CI)",
      xlim = c(0, 1),
      colgap.forest.left="2cm"

)


#Namibia


Namibia<- data.frame(
  studyid=c("Kumba et al, 2002" ),
  events=c(258),
  n= c(3098)
)

m20<-metaprop(event=events,
            n=n,
            data=Namibia,
            sm="PLOGIT", #Logit-transformed proportion
            method="Inverse", #inverse -variance weighting used PFT transformation
            method.tau="DL", #DerSimonian-Laird estimator
            method.random.ci = "HK", #Hartung-Knapp adjustment for CI
            random=TRUE,
            common=F,
            studlab= studyid
)

summary(m20)

forest(m20,
      title="Namibia: Meta-analysis of Prevalence",
      xlab="Proportion (95% CI)",
      xlim = c(0, 1),
      colgap.forest.left="2cm"

)



#Nigeria


Nigeria<- data.frame(
  studyid=c("Usman et al, 2008", "Shamaki et al, 2009", "Ehizibolo et al, 2012",
          "Mbaya et al, 2013", "David et al, 2018", "Ola-Fadunsin et al, 2018",
          "Emeto et al, 2020", "Agina et al, 2021"),
  events=c(0, 0, 5,  17, 0, 80, 2, 4),
```

```
  n= c(4, 6, 243, 65, 23, 864, 304, 200)
)

m21<-metaprop(event=events,
            n=n,
            data=Nigeria,
            sm="PLOGIT", #Logit-transformed proportion
            method="Inverse", #inverse -variance weighting used PFT transformation
            method.tau="DL", #DerSimonian-Laird estimator
            method.random.ci = "HK", #Hartung-Knapp adjustment for CI
            random=TRUE,
            common=F,
            studlab= studyid
)

summary(m21)

forest(m21,
      title="Nigeria: Meta-analysis of Prevalence",
      xlab="Proportion (95% CI)",
      xlim = c(0, 1),
      colgap.forest.left="2cm"

)

#Pakistan


Pakistan<- data.frame(
  studyid=c("Waheed et al, 1998", "Hasan et al, 2006", "Ahmed et al, 2010", "Aslam et al, 2010",
          "Gondal et al, 2010","Muieed et al, 2010","Nadeem et al, 2011",
          "Hussain et al, 2016", "Tehseen et al, 2017", "Khan et al, 2018",
          "Sabia et al, 2018", "Sobia et al, 2018"),
  events=c(85, 0, 6, 37, 2825, 16, 12, 19, 54, 58, 63, 9),
  n= c(645, 170, 150, 500, 72852, 100, 200, 283, 375, 657, 300, 52)
)

m22<-metaprop(event=events,
            n=n,
            data=Pakistan,
            sm="PLOGIT", #Logit-transformed proportion
            method="Inverse", #inverse -variance weighting used PFT transformation
            method.tau="DL", #DerSimonian-Laird estimator
            method.random.ci = "HK", #Hartung-Knapp adjustment for CI
            random=TRUE,
            common=F,
            studlab= studyid
)

summary(m22)

forest(m22,
      title="Pakistan: Meta-analysis of Prevalence",
      xlab="Proportion (95% CI)",
      xlim = c(0, 1),
      colgap.forest.left="2cm"

)
```

```
#Palestine


Palestine<- data.frame(
  studyid=c("Ereqat et al, 2020" ),
  events=c(12),
  n= c(76)
)

m23<-metaprop(event=events,
            n=n,
            data=Palestine,
            sm="PLOGIT", #Logit-transformed proportion
            method="Inverse", #inverse -variance weighting used PFT transformation
            method.tau="DL", #DerSimonian-Laird estimator
            method.random.ci = "HK", #Hartung-Knapp adjustment for CI
            random=TRUE,
            common=F,
            studlab= studyid
)

summary(m23)

forest(m23,
       title="Palestine: Meta-analysis of Prevalence",
       xlab="Proportion (95% CI)",
       xlim = c(0, 1),
       colgap.forest.left="2cm"

)

#Papua New Guinea


PapuaNG<- data.frame(
  studyid=c("Reid & Copeman, 2000" ),
  events=c(0),
  n= c(19)
)

m24<-metaprop(event=events,
            n=n,
            data=PapuaNG,
            sm="PLOGIT", #Logit-transformed proportion
            method="Inverse", #inverse -variance weighting used PFT transformation
            method.tau="DL", #DerSimonian-Laird estimator
            method.random.ci = "HK", #Hartung-Knapp adjustment for CI
            random=TRUE,
            common=F,
            studlab= studyid
)

summary(m24)

forest(m24,
       title="Papua New Guinea: Meta-analysis of Prevalence",
       xlab="Proportion (95% CI)",
       xlim = c(0, 1),
       colgap.forest.left="2cm"
```

```
)

#Paraguay


Paraguay<- data.frame(
  studyid=c("Suganuma et al, 2022" ),
  events=c(116),
  n= c(408)
)

m25<-metaprop(event=events,
              n=n,
              data=Paraguay,
              sm="PLOGIT", #Logit-transformed proportion
              method="Inverse", #inverse -variance weighting used PFT transformation
              method.tau="DL", #DerSimonian-Laird estimator
              method.random.ci = "HK", #Hartung-Knapp adjustment for CI
              random=TRUE,
              common=F,
              studlab= studyid
)

summary(m25)

forest(m25,
       title="Paraguay: Meta-analysis of Prevalence",
       xlab="Proportion (95% CI)",
       xlim = c(0, 1),
       colgap.forest.left="2cm"

)


#Philippines


Philippines<- data.frame(
  studyid=c("Dargantes et al, 2009" ),
  events=c(39),
  n= c(43)
)

m26<-metaprop(event=events,
              n=n,
              data=Philippines,
              sm="PLOGIT", #Logit-transformed proportion
              method="Inverse", #inverse -variance weighting used PFT transformation
              method.tau="DL", #DerSimonian-Laird estimator
              method.random.ci = "HK", #Hartung-Knapp adjustment for CI
              random=TRUE,
              common=F,
              studlab= studyid
)

summary(m26)

forest(m26,
       title="Philippines: Meta-analysis of Prevalence",
       xlab="Proportion (95% CI)",
```

```
      xlim = c(0, 1),
      colgap.forest.left="2cm"

)


#Saudi Arabia


SaudiArabia<- data.frame(
  studyid=c("Alanazi et al, 2018" ),
  events=c(16),
  n= c(510)
)

m27<-metaprop(event=events,
            n=n,
            data=SaudiArabia,
            sm="PLOGIT", #Logit-transformed proportion
            method="Inverse", #inverse -variance weighting used PFT transformation
            method.tau="DL", #DerSimonian-Laird estimator
            method.random.ci = "HK", #Hartung-Knapp adjustment for CI
            random=TRUE,
            common=F,
            studlab= studyid
)

summary(m27)

forest(m27,
      title="Saudi Arabia: Meta-analysis of Prevalence",
      xlab="Proportion (95% CI)",
      xlim = c(0, 1),
      colgap.forest.left="2cm"

)


#Senegal


Senegal<- data.frame(
  studyid=c("Ravel et al, 2015" ),
  events=c(6),
  n= c(86)
)

m28<-metaprop(event=events,
            n=n,
            data=Senegal,
            sm="PLOGIT", #Logit-transformed proportion
            method="Inverse", #inverse -variance weighting used PFT transformation
            method.tau="DL", #DerSimonian-Laird estimator
            method.random.ci = "HK", #Hartung-Knapp adjustment for CI
            random=TRUE,
            common=F,
            studlab= studyid
)

summary(m28)
```

```
forest(m28,
      title="Senegal: Meta-analysis of Prevalence",
      xlab="Proportion (95% CI)",
      xlim = c(0, 1),
      colgap.forest.left="2cm"

)


#Spain


Spain<- data.frame(
  studyid=c("Rodriguez et al, 2013" ),
  events=c(26),
  n= c(864)
)

m29<-metaprop(event=events,
            n=n,
            data=Spain,
            sm="PLOGIT", #Logit-transformed proportion
            method="Inverse", #inverse -variance weighting used PFT transformation
            method.tau="DL", #DerSimonian-Laird estimator
            method.random.ci = "HK", #Hartung-Knapp adjustment for CI
            random=TRUE,
            common=F,
            studlab= studyid
)

summary(m29)

forest(m29,
      title="Spain: Meta-analysis of Prevalence",
      xlab="Proportion (95% CI)",
      xlim = c(0, 1),
      colgap.forest.left="2cm"

)

#Sudan


Sudan<- data.frame(
  studyid=c("Omer et al, 2007", "Basheir et al, 2012","Salim et al, 2014",
          "Elata et al, 2020"),
  events=c(3, 0, 54, 95),
  n= c(46, 54, 509, 198)
)

m30<-metaprop(event=events,
            n=n,
            data=Sudan,
            sm="PLOGIT", #Logit-transformed proportion
            method="Inverse", #inverse -variance weighting used PFT transformation
            method.tau="DL", #DerSimonian-Laird estimator
            method.random.ci = "HK", #Hartung-Knapp adjustment for CI
            random=TRUE,
            common=F,
```

```
            studlab= studyid
)

summary(m30)

forest(m30,
      title="Sudan: Meta-analysis of Prevalence",
      xlab="Proportion (95% CI)",
      xlim = c(0, 1),
      colgap.forest.left="2cm"

)

#Thailand


Thailand<- data.frame(
  studyid=c("Camoin et al, 2019" ),
  events=c(7),
  n= c(1961)
)

m31<-metaprop(event=events,
            n=n,
            data=Thailand,
            sm="PLOGIT", #Logit-transformed proportion
            method="Inverse", #inverse -variance weighting used PFT transformation
            method.tau="DL", #DerSimonian-Laird estimator
            method.random.ci = "HK", #Hartung-Knapp adjustment for CI
            random=TRUE,
            common=F,
            studlab= studyid
)

summary(m31)

forest(m31,
      title="Thailand: Meta-analysis of Prevalence",
      xlab="Proportion (95% CI)",
      xlim = c(0, 1),
      colgap.forest.left="2cm"

)

#Turkey


Turkey<- data.frame(
  studyid=c("Marenzoni et al, 2013" ),
  events=c(0),
  n= c(346)
)

m32<-metaprop(event=events,
            n=n,
            data=Turkey,
            sm="PLOGIT", #Logit-transformed proportion
            method="Inverse", #inverse -variance weighting used PFT transformation
            method.tau="DL", #DerSimonian-Laird estimator
            method.random.ci = "HK", #Hartung-Knapp adjustment for CI
```

```
            random=TRUE,
            common=F,
            studlab= studyid
)

summary(m32)

forest(m32,
      title="Turkey: Meta-analysis of Prevalence",
      xlab="Proportion (95% CI)",
      xlim = c(0, 1),
      colgap.forest.left="2cm"

)


#Uganda


Uganda<- data.frame(
  studyid=c("Muhanguzi et al, 2017" ),
  events=c(23),
  n= c(71)
)

m33<-metaprop(event=events,
            n=n,
            data=Uganda,
            sm="PLOGIT", #Logit-transformed proportion
            method="Inverse", #inverse -variance weighting used PFT transformation
            method.tau="DL", #DerSimonian-Laird estimator
            method.random.ci = "HK", #Hartung-Knapp adjustment for CI
            random=TRUE,
            common=F,
            studlab= studyid
)

summary(m33)

forest(m33,
      title="Uganda: Meta-analysis of Prevalence",
      xlab="Proportion (95% CI)",
      xlim = c(0, 1),
      colgap.forest.left="2cm"

)


#UAE


UAE<- data.frame(
  studyid=c("Turnbull et al, 2002" ),
  events=c(3),
  n= c(62)
)

m34<-metaprop(event=events,
            n=n,
            data=UAE,
```

```
            sm="PLOGIT", #Logit-transformed proportion
            method="Inverse", #inverse -variance weighting used PFT transformation
            method.tau="DL", #DerSimonian-Laird estimator
            method.random.ci = "HK", #Hartung-Knapp adjustment for CI
            random=TRUE,
            common=F,
            studlab= studyid
)

summary(m34)

forest(m34,
      title="UAE: Meta-analysis of Prevalence",
      xlab="Proportion (95% CI)",
      xlim = c(0, 1),
      colgap.forest.left="2cm"

)

#Venezuela


Venezuela<- data.frame(
  studyid=c("Castellanos et al 2010" ),
  events=c(10),
  n= c(137)
)

m35<-metaprop(event=events,
            n=n,
            data=Venezuela,
            sm="PLOGIT", #Logit-transformed proportion
            method="Inverse", #inverse -variance weighting used PFT transformation
            method.tau="DL", #DerSimonian-Laird estimator
            method.random.ci = "HK", #Hartung-Knapp adjustment for CI
            random=TRUE,
            common=F,
            studlab= studyid
)

summary(m35)

forest(m35,
      title="Venezuela: Meta-analysis of Prevalence",
      xlab="Proportion (95% CI)",
      xlim = c(0, 1),
      colgap.forest.left="2cm"

)
```
