# Supplementary material for: Equine trypanosomiasis, a systematic review and meta‐analyses: Prevalence, morbidity and mortality
Source: Equine Vet J. 2025 Oct 23;58(2):291–319. doi: 10.1111/evj.70101 (PMC12892385; doi:10.1111/evj.70101)
Supplement: Supplementary file 3 — Data S3. Forest plots of infection rate, morbidity, mortality and death to case ration by Trypanosoma sp. (R script at end of document). [file EVJ-58-291-s006.pdf]

Equine Veterinary Journal BEVA

Data S3: Forest plots of infection rate, morbidity, mortality and death to case ratio by *Trypanosoma* sp.

Trypanosoma evansi

Infection rate

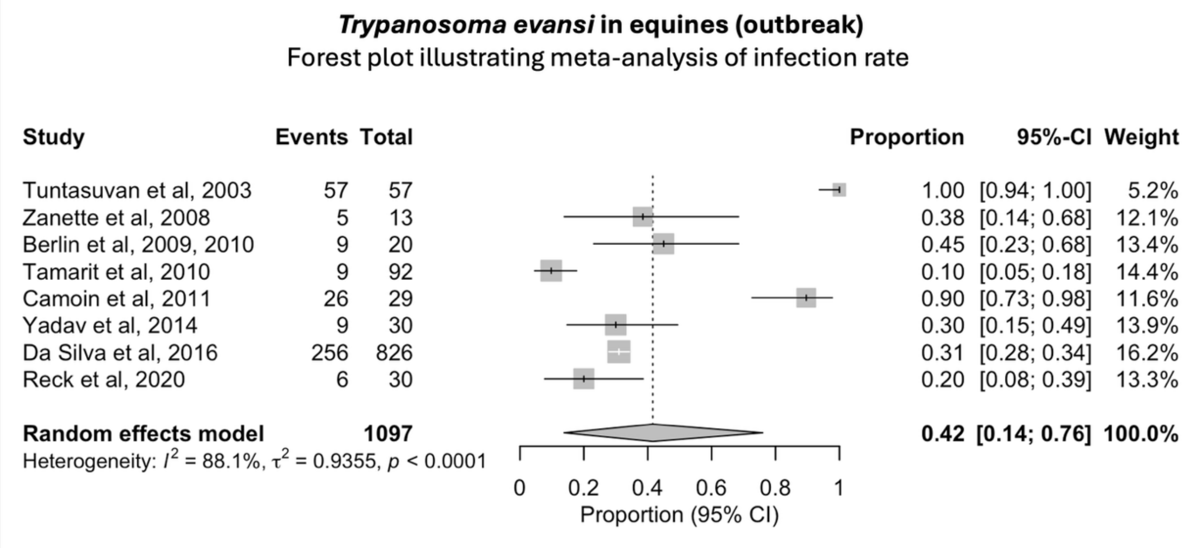

Morbidity

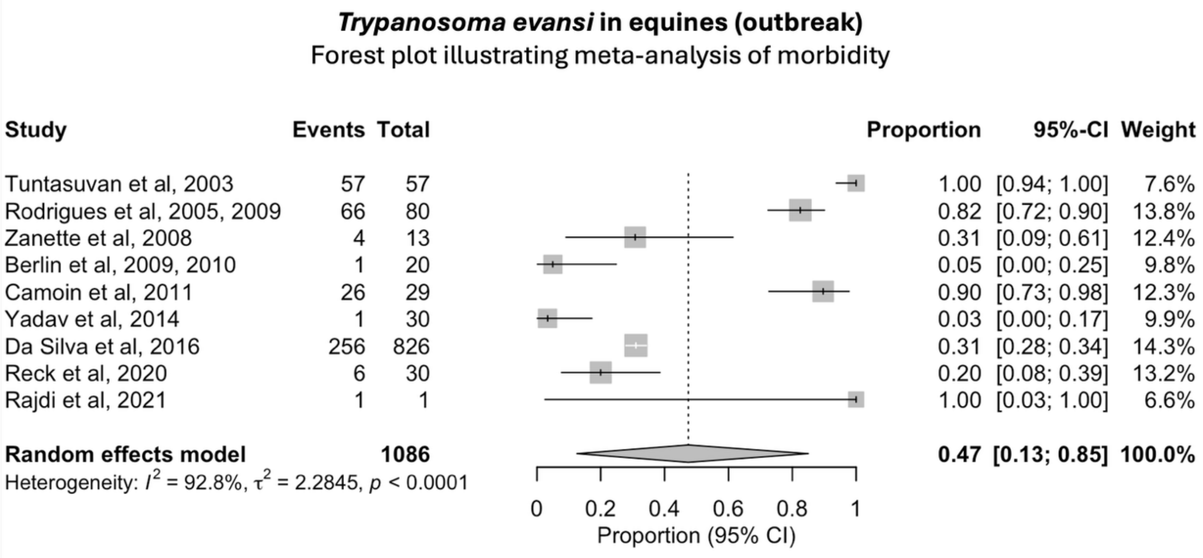

Mortality

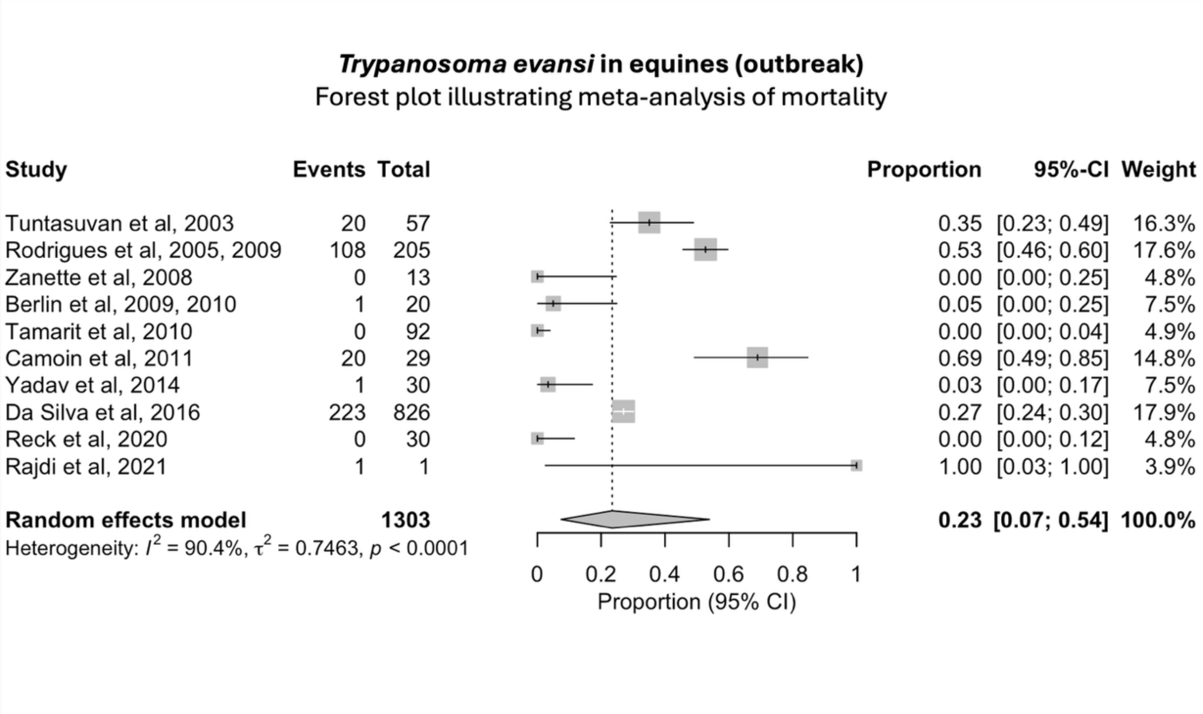

Death to case ratio

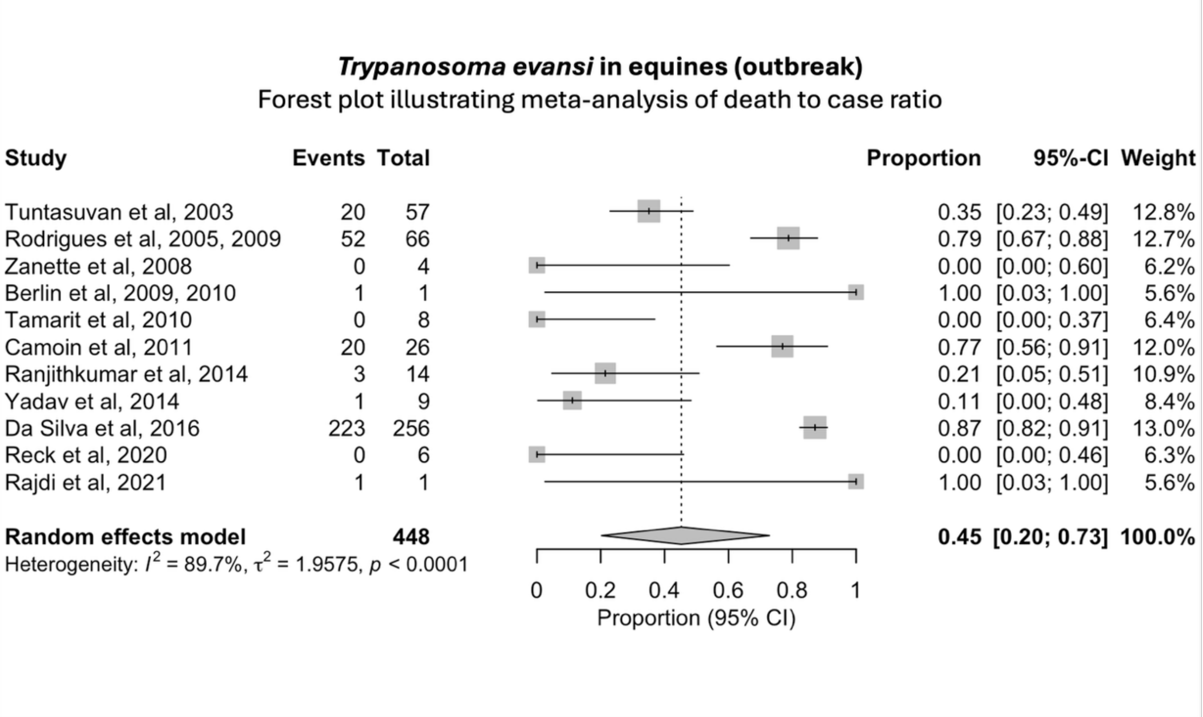

Trypanosoma equiperdum

Infection rate

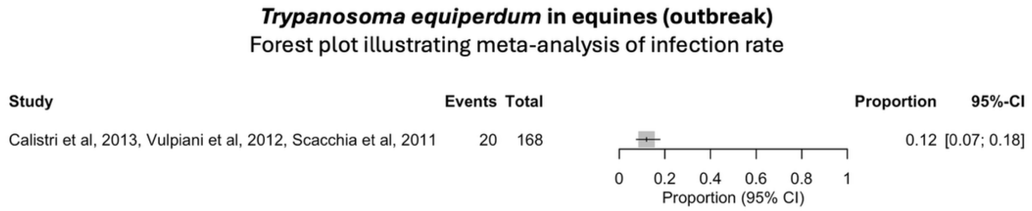

Morbidity

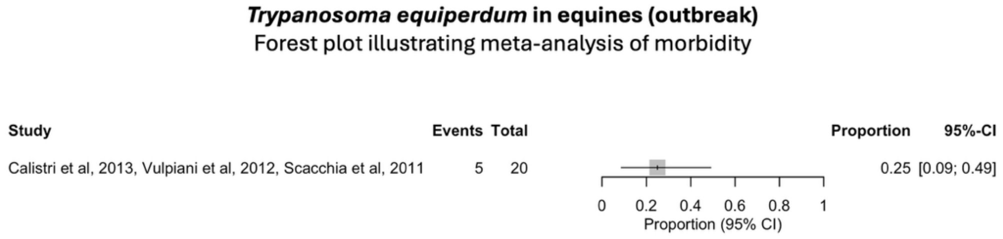

Tsetse transmitted trypanosomiasis

Infection rate and

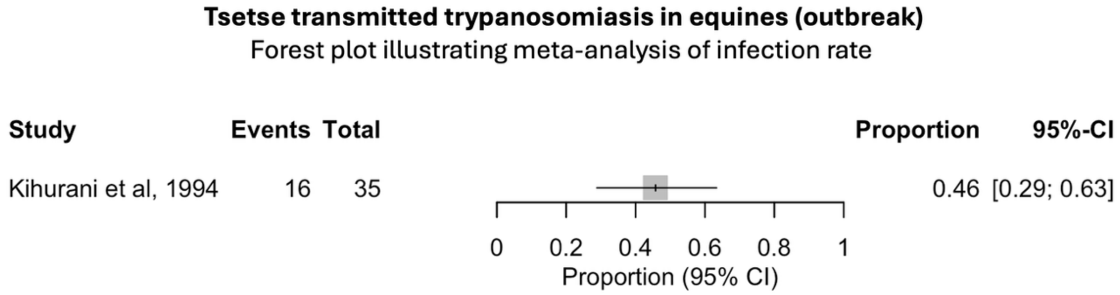

Morbidity

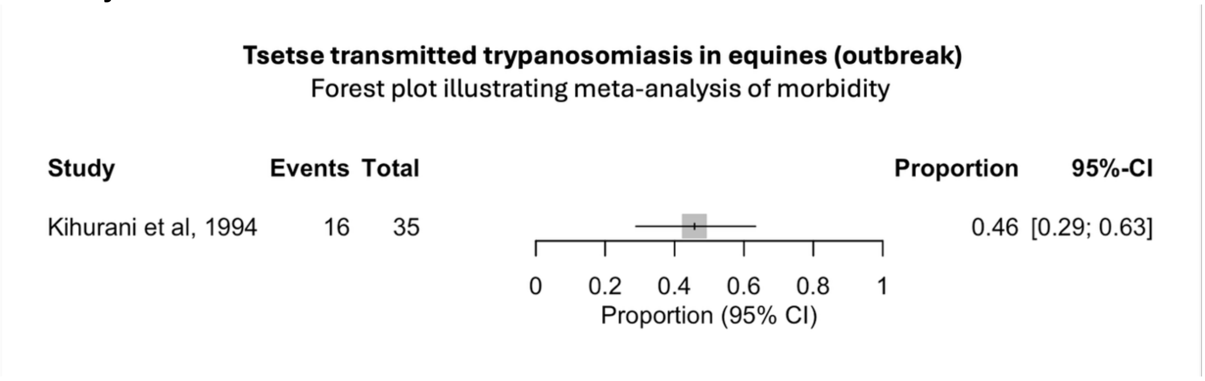

Mortality

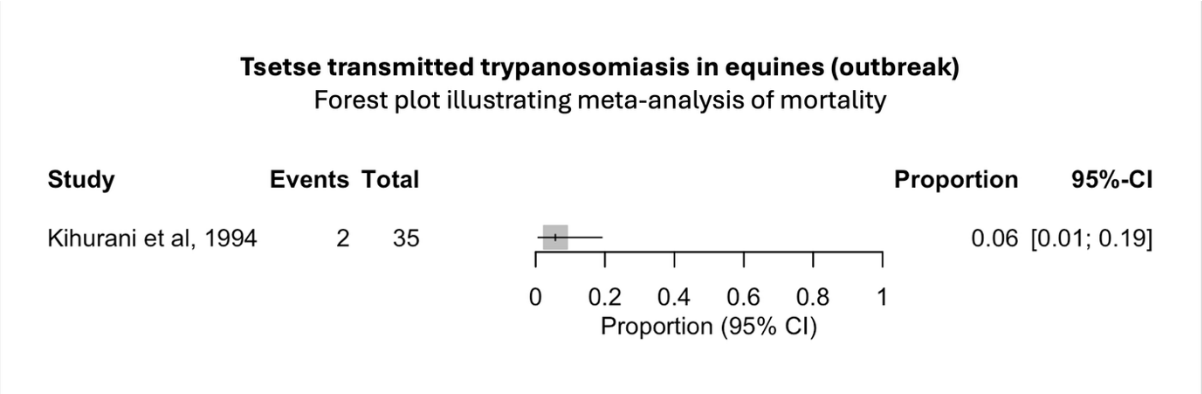

Death to case ratio

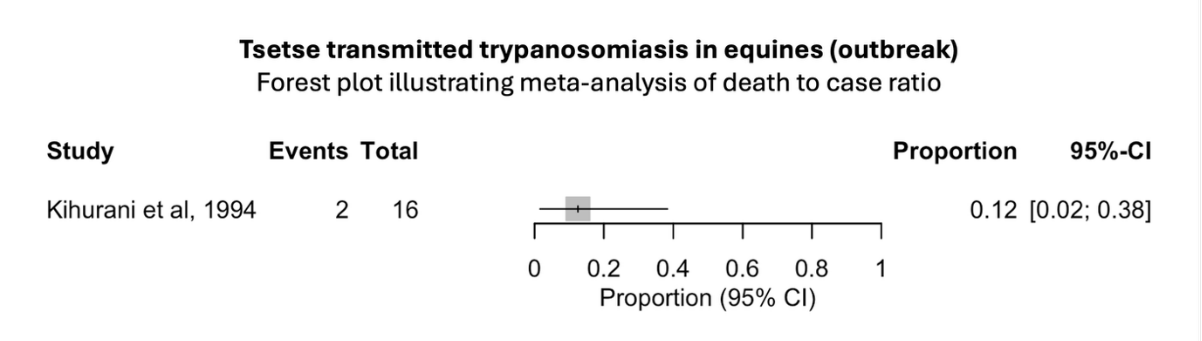

Non tsetse transmitted *Trypanosoma vivax*

Infection rate

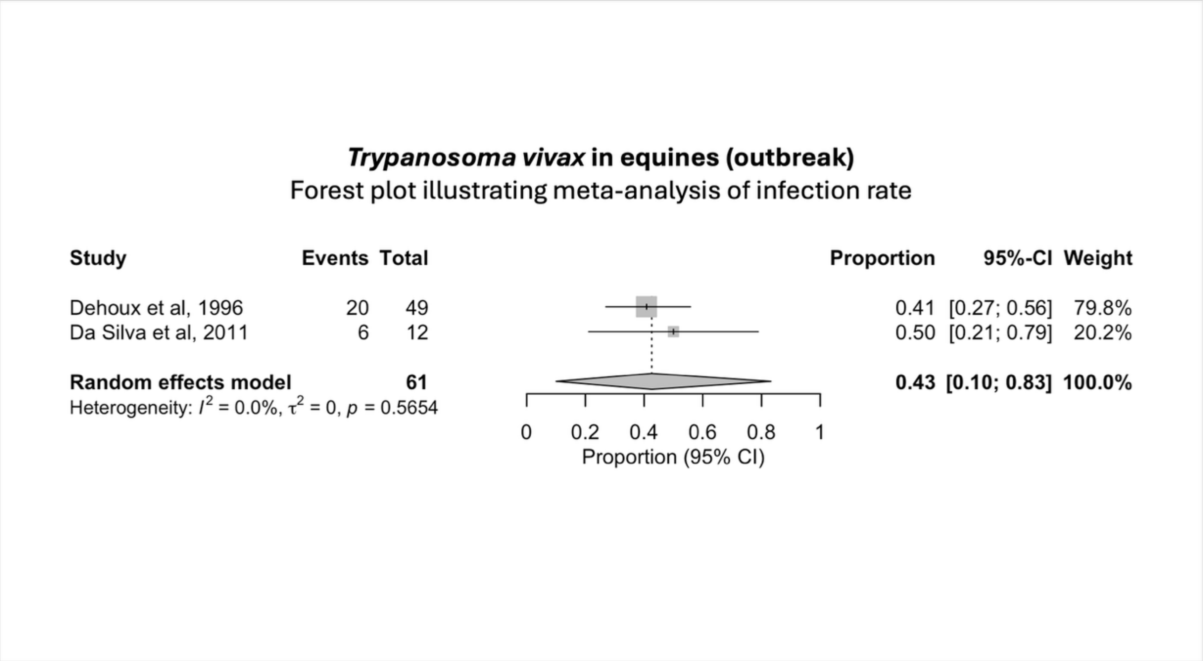

Morbidity

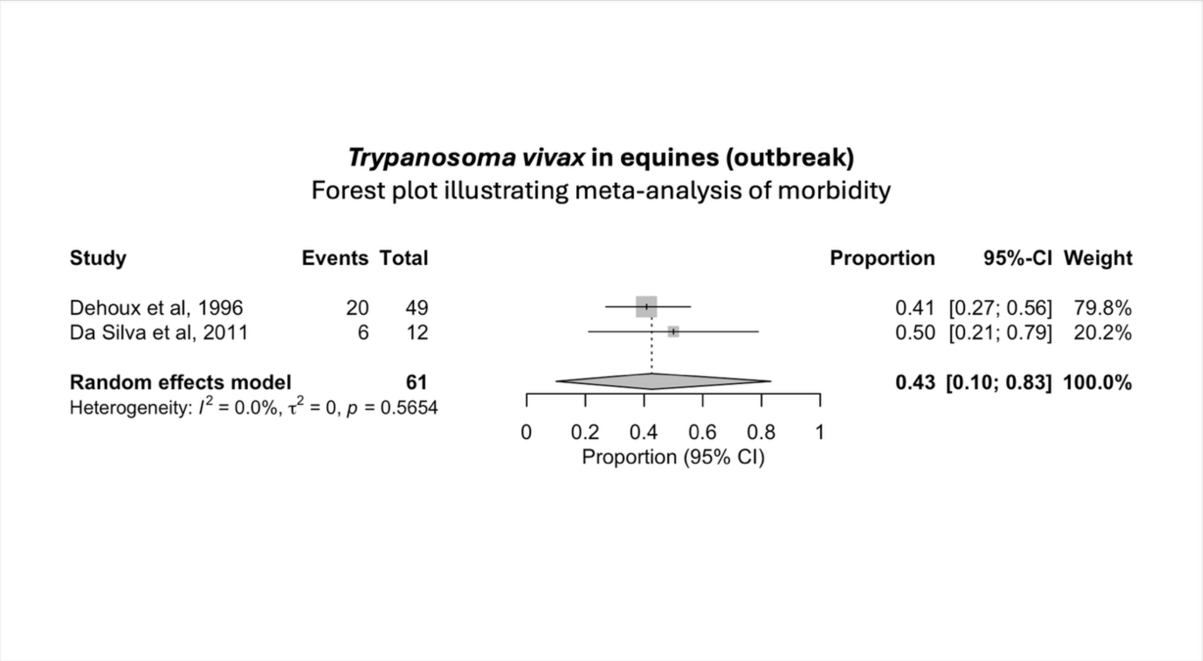

Mortality

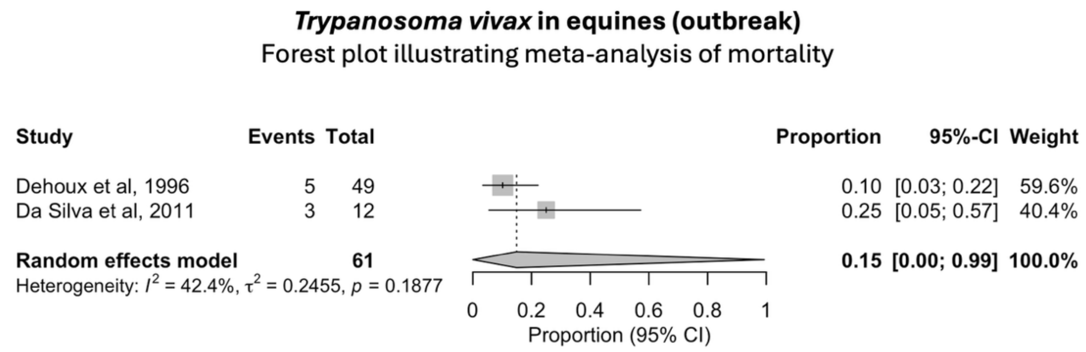

Death to case ratio

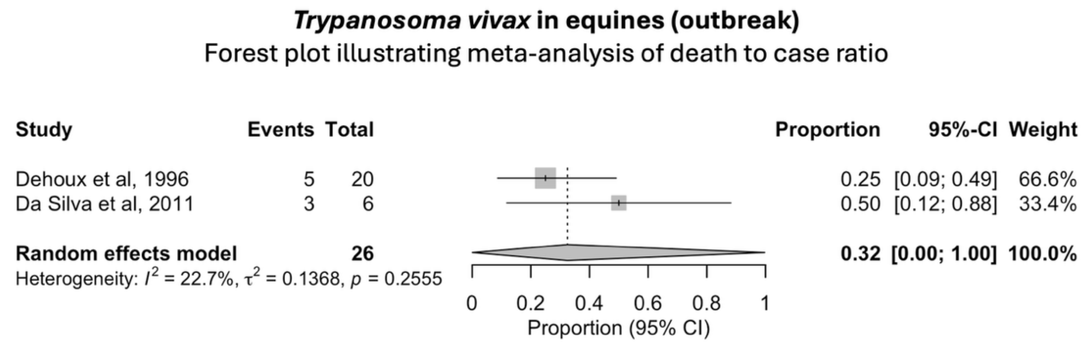

Trypanosoma evansi (endemic)

Infection rate

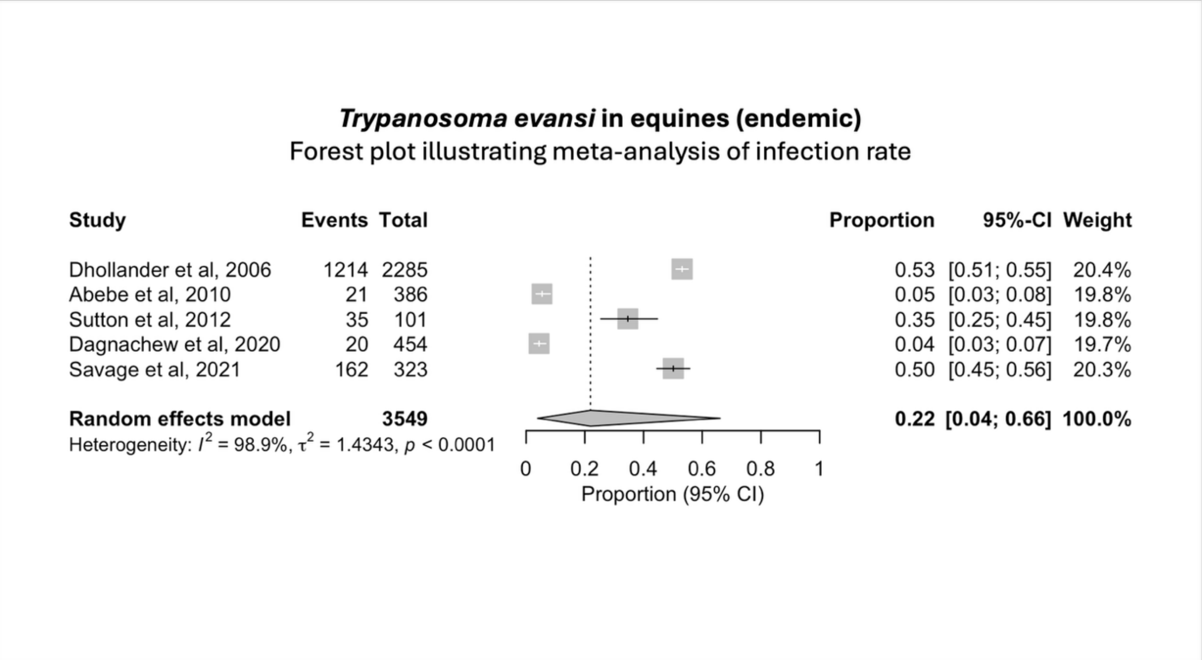

Mortality

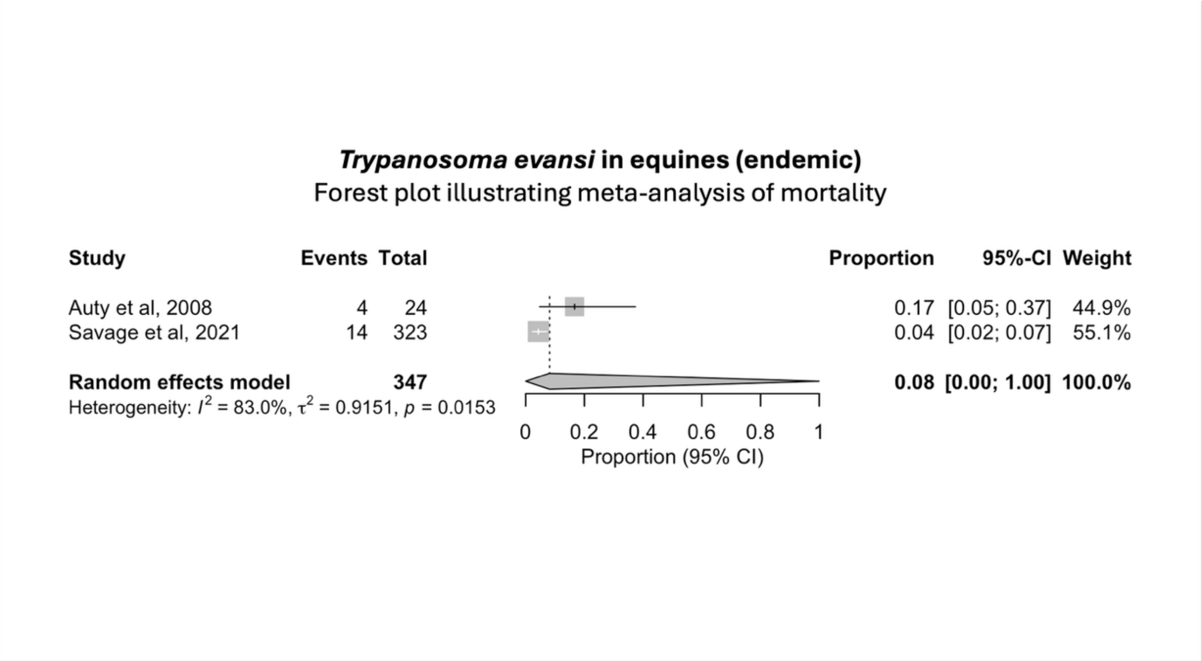

Death to case ratio

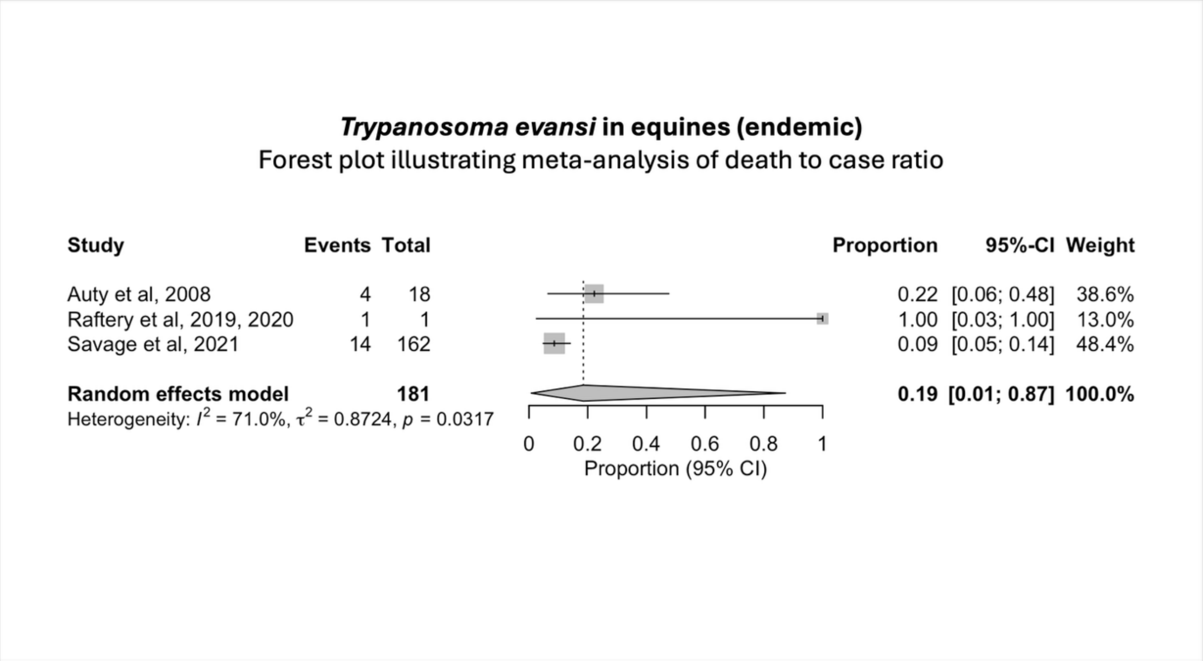

R script for meta-analyses and forest plots

```
#Use random-effects models (account for between study variability).
# Assess heterogeneity (I2 statistic)

install.packages("meta")
install.packages("metafor")

library(meta)
library(metafor)

#Trypanosoma Evansi
#infection rate
EvansiIR<- data.frame(
  studyid=c("Tuntasuvan et al, 2003", "Zanette et al, 2008","Berlin et al, 2009, 2010",
        "Tamarit et al, 2010","Camoin et al, 2011",
        "Yadav et al, 2014", "Da Silva et al, 2016", "Reck et al, 2020"),
  events=c(57, 5, 9, 9,26, 9, 256, 6),
  n= c(57, 13, 20, 92, 29, 30, 826, 30)
)

P1<-metaprop(event=events,
        n=n,
        data=EvansiIR,
        sm="PLOGIT", #Logit-transformed proportion
        method="Inverse", #inverse -variance weighting used PFT transformation
        method.tau="DL", #DerSimonian-Laird estimator
        method.random.ci = "HK", #Hartung-Knapp adjustment for CI
        random=TRUE,
        common=F,
        studlab= studyid
)

summary(P1)

forest(P1,
     title="Trypanosoma evansi: Meta-analysis of infection rate",
     xlab="Proportion (95% CI)",
     xlim = c(0, 1),
     colgap.forest.left="2cm"
)
#TEV morbidity
EvansiMorb<- data.frame(
  studyid=c("Tuntasuvan et al, 2003", "Rodrigues et al, 2005, 2009","Zanette et al, 2008",
        "Berlin et al, 2009, 2010", "Camoin et al, 2011",
        "Yadav et al, 2014", "Da Silva et al, 2016", "Reck et al, 2020",
        "Rajdi et al, 2021"),
  events=c(57, 66, 4 ,1, 26, 1 , 256, 6, 1),
  n= c(57, 80, 13, 20, 29, 30, 826, 30, 1)
)

P2<-metaprop(event=events,
        n=n,
        data=EvansiMorb,
        sm="PLOGIT", #Logit-transformed proportion
        method="Inverse", #inverse -variance weighting used PFT transformation
        method.tau="DL", #DerSimonian-Laird estimator
        method.random.ci = "HK", #Hartung-Knapp adjustment for CI
        random=TRUE,
        common=F,
```

```
            studlab= studyid
)

summary(P2)

forest(P2,
       title="Trypanosoma evansi: Meta-analysis of morbidity",
       xlab="Proportion (95% CI)",
       xlim = c(0, 1),
       colgap.forest.left="2cm"
)
studyid=c("Tuntasuvan et al, 2003", "Rodrigues et al, 2005, 2009","Zanette et al, 2008",
         "Berlin et al, 2009, 2010", "Tamarit et al, 2010","Camoin et al, 2011",
         "Ranjithkumar et al, 2014",
         "Yadav et al, 2014", "Da Silva et al, 2016", "Reck et al, 2020",
         "Rajdi et al, 2021"),


#TevansiMort

EvansiMort<- data.frame(
  studyid=c("Tuntasuvan et al, 2003", "Rodrigues et al, 2005, 2009","Zanette et al, 2008",
           "Berlin et al, 2009, 2010", "Tamarit et al, 2010","Camoin et al, 2011",
           "Yadav et al, 2014", "Da Silva et al, 2016", "Reck et al, 2020",
           "Rajdi et al, 2021"),
  events=c(20,108, 0, 1, 0, 20, 1, 223, 0, 1),
  n= c(57, 205, 13, 20, 92, 29, 30, 826, 30, 1)
)

P3<-metaprop(event=events,
            n=n,
            data=EvansiMort,
            sm="PLOGIT", #Logit-transformed proportion
            method="Inverse", #inverse -variance weighting used PFT transformation
            method.tau="DL", #DerSimonian-Laird estimator
            method.random.ci = "HK", #Hartung-Knapp adjustment for CI
            random=TRUE,
            common=F,
            studlab= studyid
)

summary(P3)

forest(P3,
       title="Trypanosoma evansi: Meta-analysis of mortality",
       xlab="Proportion (95% CI)",
       xlim = c(0, 1),
       colgap.forest.left="2cm"
)

#TEvansiDCR

EvansiDCR<- data.frame(
  studyid=c("Tuntasuvan et al, 2003", "Rodrigues et al, 2005, 2009","Zanette et al, 2008",
           "Berlin et al, 2009, 2010", "Tamarit et al, 2010","Camoin et al, 2011",
           "Ranjithkumar et al, 2014",
           "Yadav et al, 2014", "Da Silva et al, 2016", "Reck et al, 2020",
           "Rajdi et al, 2021"),
  events=c(20,52, 0, 1, 0, 20, 3, 1, 223, 0, 1),
  n= c(57,66, 4, 1, 8, 26, 14, 9, 256, 6, 1)
```

```
)

P4<-metaprop(event=events,
        n=n,
        data=EvansiDCR,
        sm="PLOGIT", #Logit-transformed proportion
        method="Inverse", #inverse -variance weighting used PFT transformation
        method.tau="DL", #DerSimonian-Laird estimator
        method.random.ci = "HK", #Hartung-Knapp adjustment for CI
        random=TRUE,
        common=F,
        studlab= studyid
)

summary(P4)

forest(P4,
    title="Trypanosoma evansi: Meta-analysis of death:case ratio",
    xlab="Proportion (95% CI)",
    xlim = c(0, 1),
    colgap.forest.left="2cm"
)

#TrypanosomaEquiperdum

EquiperdumIR<- data.frame(
  studyid=c("Calistri et al, 2013, Vulpiani et al, 2012, Scacchia et al, 2011"),
  events=c(20),
  n= c(168)
)

Q1<-metaprop(event=events,
        n=n,
        data=EquiperdumIR,
        sm="PLOGIT", #Logit-transformed proportion
        method="Inverse", #inverse -variance weighting used PFT transformation
        method.tau="DL", #DerSimonian-Laird estimator
        method.random.ci = "HK", #Hartung-Knapp adjustment for CI
        random=TRUE,
        common=F,
        studlab= studyid
)

summary(Q1)

forest(Q1,
    title="Trypanosoma equiperdum: Meta-analysis of infection rate",
    xlab="Proportion (95% CI)",
    xlim = c(0, 1),
    colgap.forest.left="2cm"
)

#EquiperdumMorbidity

EquiperdumMorb<- data.frame(
  studyid=c("Calistri et al, 2013, Vulpiani et al, 2012, Scacchia et al, 2011"),
  events=c(5),
  n= c(20)
)
```

```
Q2<-metaprop(event=events,
        n=n,
        data=EquiperdumMorb,
        sm="PLOGIT", #Logit-transformed proportion
        method="Inverse", #inverse -variance weighting used PFT transformation
        method.tau="DL", #DerSimonian-Laird estimator
        method.random.ci = "HK", #Hartung-Knapp adjustment for CI
        random=TRUE,
        common=F,
        studlab= studyid
)

summary(Q2)

forest(Q2,
    title="Trypanosoma equiperdum: Meta-analysis of morbidity",
    xlab="Proportion (95% CI)",
    xlim = c(0, 1),
    colgap.forest.left="2cm"
)

#TEQMort/ DCR
EquiperdumMort<- data.frame(
  studyid=c("Calistri et al, 2013, Vulpiani et al, 2012, Scacchia et al, 2011"),
  events=c(20),
  n= c(20)
)

Q3<-metaprop(event=events,
        n=n,
        data=EquiperdumMort,
        sm="PLOGIT", #Logit-transformed proportion
        method="Inverse", #inverse -variance weighting used PFT transformation
        method.tau="DL", #DerSimonian-Laird estimator
        method.random.ci = "HK", #Hartung-Knapp adjustment for CI
        random=TRUE,
        common=F,
        studlab= studyid
)

summary(Q3)

forest(Q3,
    title="Trypanosoma equiperdum: Meta-analysis of morbidity",
    xlab="Proportion (95% CI)",
    xlim = c(0, 1),
    colgap.forest.left="2cm"
)

#TTT
#TTTIR and morbidity
TTTIR<- data.frame(
  studyid=c("Kihurani et al, 1994"),
  events=c(16),
  n= c(35)
)

R1<-metaprop(event=events,
        n=n,
        data=TTTIR,
```

```
            sm="PLOGIT", #Logit-transformed proportion
            method="Inverse", #inverse -variance weighting used PFT transformation
            method.tau="DL", #DerSimonian-Laird estimator
            method.random.ci = "HK", #Hartung-Knapp adjustment for CI
            random=TRUE,
            common=F,
            studlab= studyid
)

summary(R1)

forest(R1,
       title="TTT: Meta-analysis of IR",
       xlab="Proportion (95% CI)",
       xlim = c(0, 1),
       colgap.forest.left="2cm"
)

#TTTMort

TTTMort<- data.frame(
  studyid=c("Kihurani et al, 1994"),
  events=c(2),
  n= c(35)
)

R2<-metaprop(event=events,
            n=n,
            data=TTTMort,
            sm="PLOGIT", #Logit-transformed proportion
            method="Inverse", #inverse -variance weighting used PFT transformation
            method.tau="DL", #DerSimonian-Laird estimator
            method.random.ci = "HK", #Hartung-Knapp adjustment for CI
            random=TRUE,
            common=F,
            studlab= studyid
)

summary(R2)

forest(R2,
       title="TTT: Meta-analysis of IR",
       xlab="Proportion (95% CI)",
       xlim = c(0, 1),
       colgap.forest.left="2cm"
)

#TTTDCR

TTTDCR<- data.frame(
  studyid=c("Kihurani et al, 1994"),
  events=c(2),
  n= c(16)
)

R3<-metaprop(event=events,
            n=n,
            data=TTTDCR,
            sm="PLOGIT", #Logit-transformed proportion
            method="Inverse", #inverse -variance weighting used PFT transformation
```

```
            method.tau="DL", #DerSimonian-Laird estimator
            method.random.ci = "HK", #Hartung-Knapp adjustment for CI
            random=TRUE,
            common=F,
            studlab= studyid
)

summary(R3)

forest(R3,
       title="TTT: Meta-analysis of DCR",
       xlab="Proportion (95% CI)",
       xlim = c(0, 1),
       colgap.forest.left="2cm"
)

#TTTEnd
#TTTeIR
TTTeIR<- data.frame(
  studyid=c("Dhollander et al, 2006", "Abebe et al, 2010",
           "Sutton et al, 2012",
          "Dagnachew et al, 2020", "Savage et al, 2021" ),
  events=c(1214, 21, 35, 20, 162),
  n= c(2285, 386, 101, 454, 323)
)

S1<-metaprop(event=events,
            n=n,
            data=TTTeIR,
            sm="PLOGIT", #Logit-transformed proportion
            method="Inverse", #inverse -variance weighting used PFT transformation
            method.tau="DL", #DerSimonian-Laird estimator
            method.random.ci = "HK", #Hartung-Knapp adjustment for CI
            random=TRUE,
            common=F,
            studlab= studyid
)

summary(S1)

forest(S1,
       title="TTTe: Meta-analysis of IR",
       xlab="Proportion (95% CI)",
       xlim = c(0, 1),
       colgap.forest.left="2cm"
)

#TTTeMORB

TTTeMORT<- data.frame(
  studyid=c("Auty et al, 2008", "Savage et al, 2021" ),
  events=c(4, 14),
  n= c(24, 323)
)

S3<-metaprop(event=events,
            n=n,
            data=TTTeMORT,
            sm="PLOGIT", #Logit-transformed proportion
            method="Inverse", #inverse -variance weighting used PFT transformation
```

```
            method.tau="DL", #DerSimonian-Laird estimator
            method.random.ci = "HK", #Hartung-Knapp adjustment for CI
            random=TRUE,
            common=F,
            studlab= studyid
)

summary(S3)

forest(S3,
      title="TTTe: Meta-analysis of Mort",
      xlab="Proportion (95% CI)",
      xlim = c(0, 1),
      colgap.forest.left="2cm"
)


#TTTeDCR

TTTeDCR<- data.frame(
  studyid=c("Auty et al, 2008", "Raftery et al, 2019, 2020","Savage et al, 2021" ),
  events=c(4, 1, 14),
  n= c(18, 1, 162)
)

S4<-metaprop(event=events,
            n=n,
            data=TTTeDCR,
            sm="PLOGIT", #Logit-transformed proportion
            method="Inverse", #inverse -variance weighting used PFT transformation
            method.tau="DL", #DerSimonian-Laird estimator
            method.random.ci = "HK", #Hartung-Knapp adjustment for CI
            random=TRUE,
            common=F,
            studlab= studyid
)

summary(S4)

forest(S4,
      title="TTTe: Meta-analysis of Mort",
      xlab="Proportion (95% CI)",
      xlim = c(0, 1),
      colgap.forest.left="2cm"
)


#Trypanosoma vivax

TvivIR<- data.frame(
  studyid=c("Dehoux et al, 1996", "Da Silva et al, 2011" ),
  events=c(20, 6),
  n= c(49, 12)
)

T1<-metaprop(event=events,
            n=n,
            data=TvivIR,
            sm="PLOGIT", #Logit-transformed proportion
            method="Inverse", #inverse -variance weighting used PFT transformation
```

```
            method.tau="DL", #DerSimonian-Laird estimator
            method.random.ci = "HK", #Hartung-Knapp adjustment for CI
            random=TRUE,
            common=F,
            studlab= studyid
)

summary(T1)

forest(T1,
      title="Tviv: Meta-analysis of IR",
      xlab="Proportion (95% CI)",
      xlim = c(0, 1),
      colgap.forest.left="2cm"
)

#TvivMort

TvivMORT<- data.frame(
  studyid=c("Dehoux et al, 1996", "Da Silva et al, 2011" ),
  events=c(5, 3),
  n= c(49, 12)
)

T2<-metaprop(event=events,
            n=n,
            data=TvivMORT,
            sm="PLOGIT", #Logit-transformed proportion
            method="Inverse", #inverse -variance weighting used PFT transformation
            method.tau="DL", #DerSimonian-Laird estimator
            method.random.ci = "HK", #Hartung-Knapp adjustment for CI
            random=TRUE,
            common=F,
            studlab= studyid
)

summary(T2)

forest(T2,
      title="Tviv: Meta-analysis of IR",
      xlab="Proportion (95% CI)",
      xlim = c(0, 1),
      colgap.forest.left="2cm"
)


#TvivDCR

TvivDCR<- data.frame(
  studyid=c("Dehoux et al, 1996", "Da Silva et al, 2011" ),
  events=c(5, 3),
  n= c(20, 6)
)

T3<-metaprop(event=events,
            n=n,
            data=TvivDCR,
            sm="PLOGIT", #Logit-transformed proportion
            method="Inverse", #inverse -variance weighting used PFT transformation
            method.tau="DL", #DerSimonian-Laird estimator
```

```
        method.random.ci = "HK", #Hartung-Knapp adjustment for CI
        random=TRUE,
        common=F,
        studlab= studyid
)

summary(T3)

forest(T3,
    title="Tviv: Meta-analysis of DCR",
    xlab="Proportion (95% CI)",
    xlim = c(0, 1),
    colgap.forest.left="2cm"
)
```
