# Supplementary material for: Equine trypanosomiasis, a systematic review and meta‐analyses: Prevalence, morbidity and mortality
Source: Equine Vet J. 2025 Oct 23;58(2):291–319. doi: 10.1111/evj.70101 (PMC12892385; doi:10.1111/evj.70101)
Supplement: Supplementary file 4 — Figure S1. PRISMA flow diagram. [file EVJ-58-291-s005.pdf]

Equine Veterinary Journal BEVA

Figure S1: PRISMA 2020 flow diagram for new systematic reviews which included searches of databases and registers only.

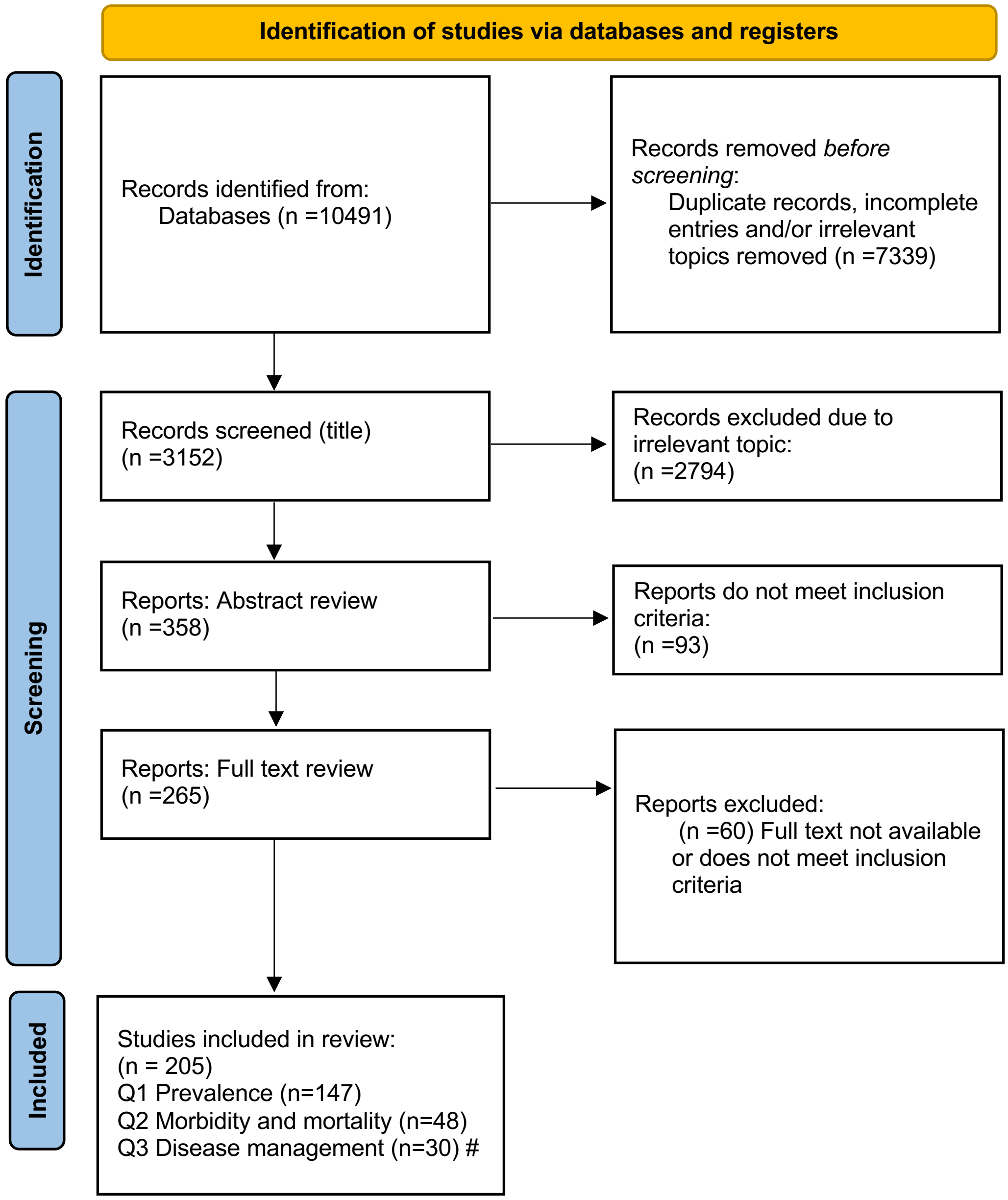

*Consider, if feasible to do so, reporting the number of records identified from each database or register searched (rather than the total number across all databases/registers).

**If automation tools were used, indicate how many records were excluded by a human and how many were excluded by automation tools.

Study question 3 is the subject of 'Equine trypanosomiasis: a systematic review of disease management' (under review)

From: Page MJ, McKenzie JE, Bossuyt PM, Boutron I, Hoffmann TC, Mulrow CD, et al. The PRISMA 2020 statement: an updated guideline for reporting systematic reviews. BMJ 2021;372:n71. doi: 10.1136/bmj.n71

For more information, visit: http://www.prisma-statement.org/
