# Supplementary material for: Equine trypanosomiasis, a systematic review and meta‐analyses: Prevalence, morbidity and mortality
Source: Equine Vet J. 2025 Oct 23;58(2):291–319. doi: 10.1111/evj.70101 (PMC12892385; doi:10.1111/evj.70101)
Supplement: Supplementary file 6 — Table S2. Quality assessment scoring system for prevalence data. [file EVJ-58-291-s009.pdf]

**Table S2:** Quality assessment checklist for prevalence studies.

To assess risk of bias, adapted from Hoy *et al* [1] to be appropriate to an animal based study.

| | Risk of bias items | Risk of bias levels | Point scored |
|---|---|---|---|
| 1 | Was the sampling frame a true or close representation of the national population? | Yes (LOW RISK) | 0 |
| | | No (HIGH RISK) | 1 |
| 2 | Was some form of random selection used to select the sample? | Yes (LOW RISK) | 0 |
| | | No (HIGH RISK) | 1 |
| 3 | Was a sample size calculation done? | Yes (LOW RISK) | 0 |
| | | No (HIGH RISK) | 1 |
| 4 | Were the study subjects and setting described in detail? | Yes (LOW RISK) | 0 |
| | | No (HIGH RISK) | 1 |
| 5 | Was an acceptable case definition used in the study? | Yes (LOW RISK) | 0 |
| | | No (HIGH RISK) | 1 |
| 6 | Was the diagnostic method appropriate? | Yes (LOW RISK) | 0 |
| | | No (HIGH RISK) | 1 |
| 7 | Was the same mode of data collection used for all subjects? | Yes (LOW RISK) | 0 |
| | | No (HIGH RISK) | 1 |
| 8 | Was there appropriate statistical analysis? | Yes (LOW RISK) | 0 |
| | | No (HIGH RISK) | 1 |
| **Summary** | **Overall risk of study bias** | **LOW RISK** | **0-2** |
| | | **MODERATE RISK** | **3-5** |
| | | **HIGH RISK** | **6-8** |

1. Hoy, D., Brooks, P., Woolf, A., Blyth, F., March, L., Bain, C., Baker, P., Smith, E. and Buchbinder, R. (2012) Assessing risk of bias in prevalence studies: modification of an existing tool and evidence of interrater agreement. *Journal of Clinical Epidemiology* **65**, 934–939.
